# Unveiling Valuable Secondary Metabolites from the Bioconversion of Banana (*Musa balbisiana*) Peel-Derived Biomass with *Aspergillus niger*. Metabolomic Insights into the Chemical Profiles

**DOI:** 10.1021/acsomega.5c10614

**Published:** 2026-01-21

**Authors:** Jhuly Wellen Ferreira Lacerda, Giovanna Amaral Filipe, Lucas Pradi, Tatiane de Andrade Maranhão, Diogo Robl, Louis Pergaud Sandjo

**Affiliations:** † Department of Chemistry, Universidade Federal de Santa Catarina, 28117 Campus Universitário-Trindade, 88040-900 Florianópolis, SC, Brazil; ‡ Laboratory of Microorganisms and Biotechnological Processes, Department of Microbiology, Parasitology and Immunology, Universidade Federal de Santa Catarina, Campus Universitário-Trindade, 88040-900 Florianópolis, SC, Brazil

## Abstract

Santa Catarina is Brazil’s fourth-largest banana producer, generating a significant amount of waste. Banana peel conversion into useful products is uncommon. However, it produces natural products that possess benefits for human health or can serve as biomass to produce fungi-derived metabolites. Therefore, *Aspergillus niger* was isolated from banana peels and then cultured on three distinct media composed of starch, banana peels (BP), and starch-enriched banana peels (BPS) for 14, 21, and 28 days at 28 °C. Their chemical profiles were established using liquid chromatography coupled to mass spectrometry data assisted by the GNPS database and MS-FINDER software. In BP media, 76 secondary metabolites were detected; among them, 66 were produced by the fungus. Twenty-seven compounds, such as nigragillin and pyranonigrin A, are *A. niger* chemomarkers, while other substances, except for the 13 unidentified, were previously found in other organisms. Among the 99 metabolites detected in BPS media, 22 were previously identified as *A. niger*-derived compounds. BPS media also yielded 26 unidentified compounds, while 12 annotated ones were also found in BP. The remaining metabolites were previously reported in other organisms. Principal component 1 of 44.8% and principal component 2 of 22.2% indicated that 21-day fermentation for BP medium and 28-day fermentation for BPS medium were the optimal conditions to produce secondary metabolites. Variable importance plots reveal nonadecan-2-amine (score >3) and an alkylaryl amine derivative (*m*/*z* 304.3005) as the most concentrated compounds. The present results showed a cheap process to turn food residues and waste into valuable molecules.

## Graphical abstract

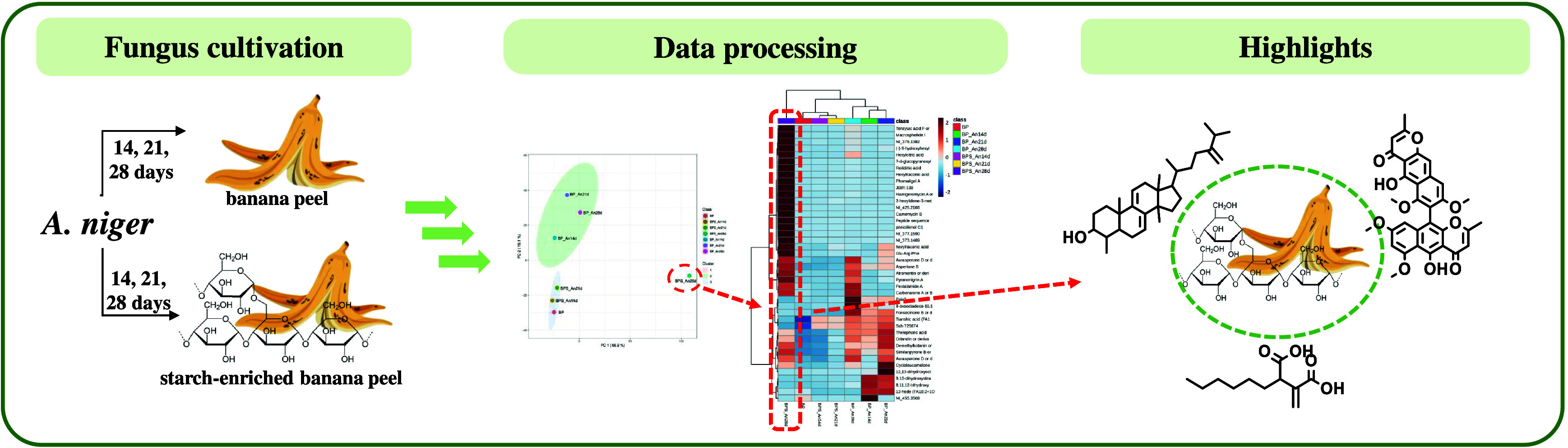

## 1 Introduction

Growing urbanization and lifestyle changes have colossally increased waste production, especially food residue and waste (FRW) from industries, agriculture, and households. According to the UN Environment Program, 1052 million tons of organic waste/residue worldwide were from food, with almost 2% produced in Brazil in 2022.^1,2^

Based on the report from the Brazilian Institute of Geography and Statistics, a unit of the Brazilian Ministry of Planning and Budget, Santa Catarina state in 2024 was the fourth largest producer of bananas in Brazil (IBGE, 2024).^3^ Banana peel residues account for 14% of waste in banana farming.^4^ Poor management of these wastes can cause environmental impacts (gas emissions, contaminated groundwater, and noxious odors) and consequences for economic income associated with natural resources such as water and land. A variety of sustainable techniques are currently used to reduce the impact of FRW. Although in some cases FRW is used to produce energy, compost, animal feed, and biofertilizers, in others, it is sent to landfills or incinerated. Other strategies, such as biotechnology processes that produce valuable bioactive compounds from FRW, contribute to the bioeconomy initiative. These bioactive compounds can be turned into food supplements or food ingredients known to possess health benefits; they can also contribute to drug research and discovery.^5^ Therefore, FRW can undergo organic solvent extraction and purification to identify these valuable substances.^6^ More effective management and wider integration into economic, research, and agricultural sectors are important to mitigate the impact of FRW, particularly banana peels. As shown in previous studies, banana peels have nutritive values and can be considered a functional food because of their content of minerals (iron, calcium, magnesium, and sodium), amino acids (valine, threonine, and phenylalanine), phenolic acids (gallic, tannic, ferulic, and caffeic acids), flavonoids (rutin and quercetin), carotenoids (β- and α-carotene), terpenoids (stigmasterol), and fatty acids (palmitic acid).^7,8^

Thus, banana peels can also be used as biomass for microbial fermentation to generate high-value products.^6^ This last process is known to produce flavoring for fermented beverages; lactic, citric, and succinic acids; enzymes (lipase, amylase, cellulase, and others); ethanol; and hydrogen.^9–13^ A previous study reported that solid- and liquid-state cultivation of *Aspergillus niger* using orange peels as a substrate produced protease.^14^ In addition, *Streptomyces* sp. grown on cassava peel, groundnut shell, or wheat bran-based solid-state cultures produced neomycin, oxytetracycline, and meroparamycin.^15,16^ Therefore, the use of FRW as biomass is fundamental for a circular bioeconomy. Based on the aforementioned information, this work aimed to investigate the chemical response of *A. niger* when grown on a biomass composed of banana peels. The species *A. niger* was selected for this study because it is an airborne fungus with wide application in industrial biotechnology and because it is prevalent in household waste used for biomass, particularly in banana peels used in this work. Experiments using *A. niger* cultivated on FRW biomasses such as wheat bran, coffee pulp, pineapple canary waste, tomato waste, and apple pomace produced citric acid, lycopene, biosorbents, vanillic acid, vanillin, and polygalacturonase.^17^ The profile of secondary metabolites varies according to the type of FRW, representing an advantage in the identification of new byproducts and valuable chemical substances with potential applications in drug discovery and as bioinoculants, fungicides, and biostimulants, among others. Previous studies used banana peels as biomass to grow *A. niger* to produce bioethanol, citric acid, and enzymes such as xylanase, amylase, and pectinase.^18–20^

In general, the use of food waste for fungal cultivation not only produces valuable substances but also represents an economical process when compared with conventional techniques that produce metabolites derived from microorganisms using purchased items, including biomass (rice, agar, and starch) and other nutrients. In this study, the hypothesis was to investigate whether organic waste, such as banana peels, can serve as biomass for microorganisms, enabling the biosynthesis of biologically relevant compounds. Furthermore, emphasis was placed on the secondary metabolites of banana peel and their impact on the chemical response of *A. niger*.

To date, secondary metabolites generated by *A. niger* grown on biomass made of banana peels have never been investigated.

We herein report a comparative study using the dereplication technique and statistical tools to analyze LC-MS chemical profiles of *A. niger* cultivated on banana peels, starch-enriched banana peel, and starch. In addition to the culture media used for *A. niger* growth, fermentation periods of 14, 21, and 28 days were also used as comparison parameters.

## 2 Materials and Methods

### 2.1 Materials

Acetonitrile, methanol, water, isopropanol (LCMS grade), and leucine entkephalin were obtained from Servylab, a Sigma-Aldrich seller (Rio Grande do Sul, Brazil). The syringe filter (0.22 μM, hydrophobic), vials (2 mL), and hydrophobic and hydrophilic filter membranes for solvent filtration were furnished by Filtrilo (Paraná, Brazil). Ethyl acetate of analytical grade was furnished by Rauter (Gravata, Rio Grande do Sul).

### 2.2 Fungus Collection and Characterization

Banana peels (*Musa balbisiana*), popularly known as “banana *figo*”, were purchased from a local market in the city of Florianópolis, Santa Catarina, Brazil. To reproduce how microorganisms colonize and consume organic matter in nature and to make sure that the selected microorganism will grow on this biomass, the peels were washed in running water to remove contaminants, and then, a quantity (5 g) was ground in 50 mL of distilled water in a beaker. The beaker containing this organic material was kept in the lab for 7 days until colonies of microorganisms appeared. The predominant fungus was isolated on Potato Dextrose Agar plates and grown for 21 days at 28 °C. Further, the strain was identified by micromorphological characterization, applying the adapted Riddell technique,^21^ and confirmed by the amplification and sequencing technique of the ITS gene (ITS region: ITS1), performed by the biotechnology company Neoprospecta in Florianópolis, Santa Catarina, Brazil. The sequence was registered in the NCBI database under GenBank number PX619933.

### 2.3 Substrate Preparation and Inoculation

The spore suspension used as inoculum was prepared by adding Tween 80 (0.01% v/v to the fungus colony previously cultivated on BDA plates at 28 °C for 7 days, following the protocol described by Robl et al. (2015).^22^ The spore counting was performed by using a Neubauer chamber. Initially, the concentrated suspension was diluted 10x in an Eppendorf tube. After homogenization using a vortex mixer, two 20 μL aliquots of the diluted solution were transferred to the chamber. By using a microscope with 40× magnification, the spores present in the five diagonal squares of the central quadrant of the chamber were counted. The spore concentration in the suspension was calculated using the following equation:

$$\mathrm{concentration}\;\left(\mathrm{spores}\cdot {\mathrm{mL}}^{-1}\right)=\frac{\mathrm{dilution factor}\cdot 2.5\times 10^{5}\cdot \mathrm{spore count}}{5}$$

The spore suspension was diluted in order to reach the desired final concentration of 10^6^ spores·mL^–1^ in the culture medium.

Three flasks received a ratio of 50 g of banana peel to 1 L of deionized water as the culture medium. These flasks were covered with cotton and autoclaved at 121 °C, 1 atm for 30 min.^23^ Soon after, at room temperature and within a laminar flow, the inoculum of *A. niger* was added to the culture media and incubated in B.O.D. at 28 °C. A similar procedure was performed with starch in a concentration of 50 g/L.

The mixture of starch and banana peels to formulate an alternative culture medium was motivated by previous works whose results indicate that varying carbohydrate sources (such as sucrose, lactose, mannitol, potato dextrose, flour, and starch) can influence fungal growth and the biosynthesis of specific biomolecules.^24,25^ Thus, the impact of supplementing the banana peel biomass medium with starch as a carbon source was assessed in relation to the profile of secondary metabolites. Starch quantity was not assessed as a culture parameter, but literature suggests 20 g/L is optimal for *A. niger* growth.^26^ Thus, a 1:1 (w/w) banana peel/starch mix totaling 50 g in 1 L of distilled water to cultivate the fungus was prepared in three different flasks. The cultivation was carried out in the periods of 14, 21, and 28 days at a constant temperature of 28 °C. Thus, three culture media containing only banana peel (**BP_An**), three containing banana peels enriched with starch (**BPS_An**), and three containing starch (**Starch**) were obtained.

### 2.4 Preparation of Crude Extracts

After each time of fungus cultivation, ethyl acetate was added to stop the fungus growth and perform the extraction. The organic extract was obtained with 200 mL of ethyl acetate assisted by sonication in an ultrasonic bath for 1 h, following the solid–liquid extraction process; these steps were repeated three times for each medium, thus obtaining a total of 6 extracts. The extracts were concentrated by rotary evaporation. As intended to obtain a blank for the extracts, this same extraction procedure was applied to samples from inoculated starch, noninoculated banana peel (**BP**), and noninoculated starch-enriched banana peels. The aim was to discriminate banana-peel- and starch-derived metabolites from those produced by the fungus.

### 2.5 Ultra-Performance Liquid Chromatography-Tandem Mass Spectrometry Analysis (UPLC-ESI-MS/MS)

The obtained extracts were prepared by dissolving 1.6 mg in 2 mL of methanol/acetonitrile (1:1 v/v, LC-MS grade), resulting in a concentration of 800 μg/mL. Before analysis, all extracts were filtered through a 0.22 μm hydrophilic PTFE filter (Analytical).

An Acquity UPLC system class H (Waters Co., USA) equipped with a photodiode array detector (PDA), sample manager, and a quaternary solvent manager as well as a BEH C18 column (50 mm, 1.0 mm, particle size 1.7 μm (Waters)) was used for the separation. The column and the sample tray were maintained at temperatures of 40 and 20 °C, respectively. Each sample (2 μL) was injected and analyzed under gradient conditions at a flow rate of 0.3 mL/min. The mobile phase was composed of H_2_O containing 0.1% formic acid, pH 3.0 (A) and LC-MS-grade acetonitrile (B). The gradient was the following: between 0 and 1 min, 90% A/10% B; between 1 and 12 min, 10% A/90% B; between 12 and 14 min, 10% A/90% B; between 14 and 15 min, 90% A/10% B; and between 15 and 20 min, the initial mixture was applied to equilibrate the column.

A mass spectrometer, Xevo G2-S QTof (Waters), bearing an electrospray ionization (ESI) probe operating in positive and negative ionization modes, coupled to the UPLC device was used to detect the chemical components of the extracts. Nitrogen was used as nebulizer gas; chromatograms were acquired with the cone gas flow of 100 L/h, the desolvation gas flow of 900 L/h, the sampling cone current of 40 V, and the source offset current of 80 V. The collision gas was argon, and the lockspray reference sample was leucine enkephalin with reference mass values at *m*/*z* 554.2615 (ESI−) and 556.2771 (ESI+). The desolvation and the ionization source were maintained during the analyses at 250 and 90 °C, respectively, while the capillary voltage was 3 kV. A range of 30–35 eV was used as the collision energy. Data were acquired over a range of 50–1500 Da in positive mode and 50–1500 Da in negative mode, at a scan time of 1.0 s over 20 min, and were processed with MassLynx V4.1 (Waters). The MS data were acquired in Fast Data-Dependent acquisition (Fast DDA) and MS^E^ (DIA) settings using argon as collision gas and applying the energy range of 25–35 eV (Supporting Information).

### 2.6 Data Processing by MS DIAL/MS FINDER

A combination of MS-DIAL (version 4.92) and MS-FINDER (version 3.52) software was used to assist in establishing the chemical profile. The raw data from the Fast DDA analyses were loaded directly into MS-DIAL, where the information was selected according to the analyses performed by UPLC-MS, and sections for data processing and alignment were selected. The values applied to the parameters were: tolerance of 0.02 and 0.03 Da for MS1 and MS2, respectively; peak detection amplitude of 10000 for the minimum peak height and 0.1 Da for the mass cutoff width; for deconvolution, a sigma window value of 0.5 and an MS/MS abundance cutoff of 30 amplitudes; the retention time tolerance was set to 0.5 min and 0.02 Da for MS^1^ tolerance. The option to remove features based on solvent blank data was also applied, thus obtaining annotations referring only to the samples.^27^

The data obtained in MS-DIAL were exported to MS-FINDER, where the identification of features was provided by comparing experimental MS/MS fragmentation spectra from several spectral databases (COCONUT, PubChem, UNPD, ChEBI, NANPDB, KNApSAcK, NPA). To select the structure as a potential candidate, a score value greater than or equal to 5 among the fragments obtained and knowledge of the possible classes of compounds that the sample type can produce were considered.^28^

The features that did not lead to structure proposals by the software were subjected to the dereplication technique using MS^E^ fragmentation data in comparison with those reported in the literature. The molecular formulas were determined by using the MassLynx elemental composition tool. Each molecular formula was chosen with a tolerance lower than or equal to ±5 ppm between the calculated and measured mass values.

### 2.7 Molecular Network by GNPS

The raw Fast-DDA data acquired by UPLC-MS for the extracts were converted into the corresponding format (mzML) using MSConvert software (ProteoWizard, Palo Alto, CA, USA) and uploaded to the GNPS online platform using Core FTP LE software v. 2.2. Molecular network analysis was performed following the online workflow (https://ccms-ucsd.github.io/GNPSDocumentation/) described on the GNPS web site.^29^ Molecular networks were obtained using a precursor ion mass tolerance of 0.02 Da and an MS/MS fragment ion tolerance of 0.03 Da. Network edges were produced only with a cosine score above 0.77 and a minimum match of 4 peaks in the fragmentation spectrum. The spectral data in the network were then compared with the GNPS spectral libraries. For matches of the GNPS library with the data, a score above 0.7 and at least 4 matching peaks were applied. The generated molecular networks were visualized using Cytoscape software, version 3.10.2.

### 2.8 Statistical Analysis

For statistical analysis, peak intensity data deconvolved by MS-DIAL were extracted and reorganized into a table in CSV format. Univariate and multivariate statistical analyses were performed on the MetaboAnalyst 6.0 online platform (https://www.metaboanalyst.ca/) to discriminate significant metabolites between the culture media (BP_An, BPS_An, noninoculated BP and starch), as well as the metabolites at different growth times (14, 21, and 28 days). Data were normalized by square root transformation and Pareto scaling. Principal component analysis (PCA) and partial least squares discriminant analysis (PLS-DA) were used to characterize the distribution of the data set and determine the main metabolites responsible for the distribution, the latter based on the thresholds of the variable importance in projection (VIP) value >1 and *p*-value <0.05. Heat plots were applied to visualize the relationship between the samples and the abundances of the compounds in each sample.

## 3 Results and Discussion

### 3.1 Fungal Isolation and Cultivation

The isolated strain was identified through micromorphological features as an *Aspergillus* species (Figure 1 and Figure S1–S3), applying an adapted Riddell technique.^21^ Using the slide microcultivation technique, it was possible to verify the microscopic characteristics of the fungal isolate under an optical microscope. Classic characteristics of the fungus *A. niger* were observed, including abundant black conidiophores and conidia and a smooth hyaline wall.

**Figure 1:**
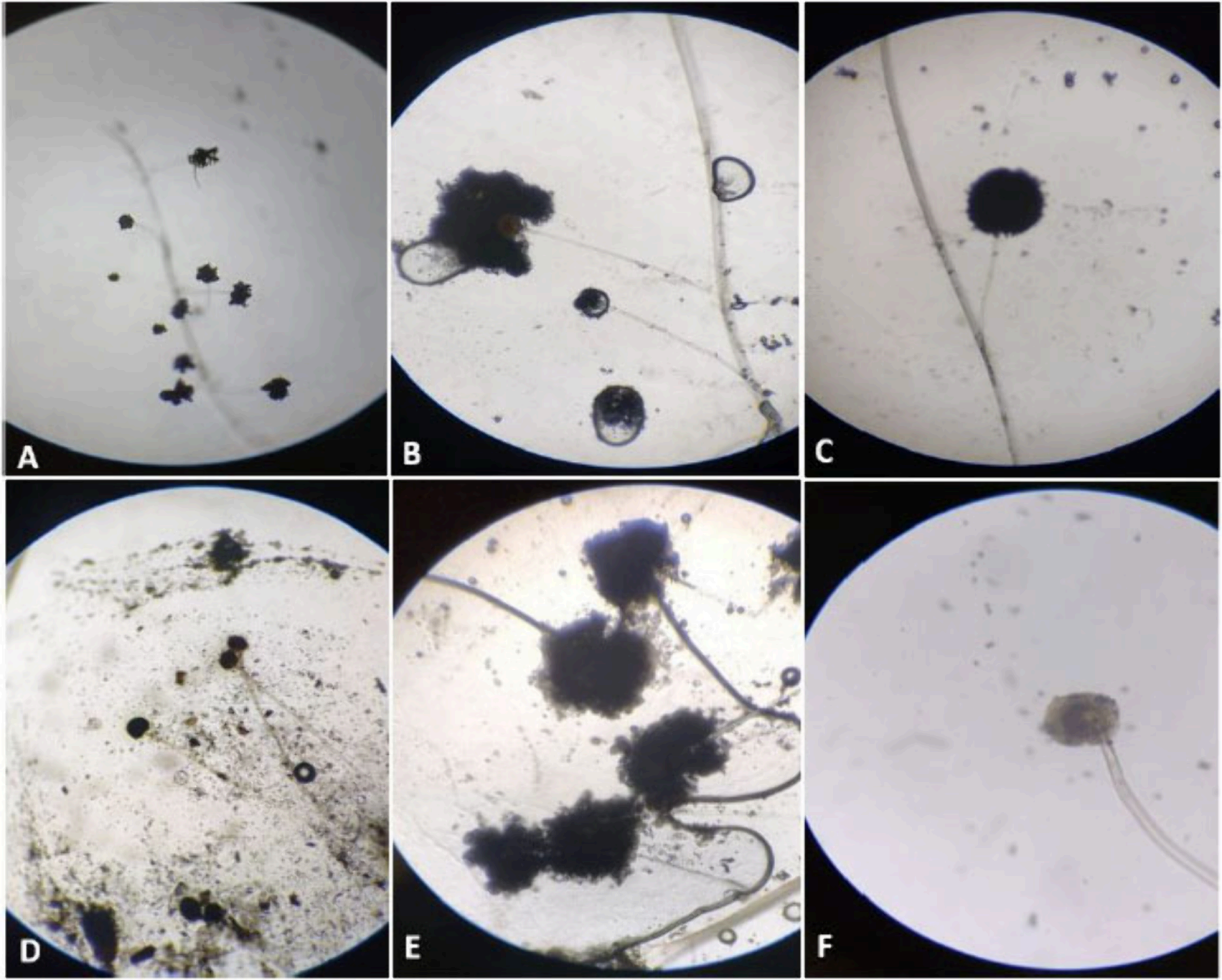
Micromorphological aspect of the fungus evaluated at 7 and 14 days of cultivation (micro cultivation). (A–C) 7 days of micro cultivation, (D–F) 14 days of micro cultivation. (A and D) 20× magnification; (B, C, E. F) 40× magnification.

Molecular identification using amplification and sequencing techniques of the ITS gene region (ITS1) matched the strain of *Aspergillus niger* with 99% homology. Sequencing of this region of the fungus, renamed *Aspergillus niger* LQPN001, resulted in the following sequence:

TTACCGAGTGCGGGTCCTTTGGGC​CCAACCTC​CCATC​CGT​GTC​TAT​TGTA​CCCTG​TTG​CTT​CGC​GGAC​CCG​CCG​CTT​GTC​GGC​CGC​CGG​GGG​GGC​GCC​TCT​GCCC​CCC​GGG​CCC​GTG​CCC​GCC​GGA​GAC​CCC​AAC​ACG​AACAC​TGT​CTG​AAA​GCGT​GCA​GTC​TGA​GTT​GA​TTGA​AT​GCA​ATC​AGT​TAA​AAC​TTT​CAA​CAA​TGG​ATCT​CTT​GGT​TCCG

The BLASTN search resulted in four species with 99.54% similarity of the ITS genetic code, all with E-value of 2–111 and 100% coverage of the searched gene: *Aspergillus fetidus* (CBS 121.28), *Aspergillus awamori* ATCC 16877 (CBS 557.65), *Aspergillus niger* ATCC 16888 (CBS 554.65), and *Aspergillus welwitschiae* (CBS:139.54).

The fungus, *A. niger*, was grown on *Musa balbisiana* banana peels, starch-enriched peels, and starch for 14, 21, and 28 days, producing nine crude extracts: BP_An14d, BP_An21d, BP_An28d, BPS_An14d, BPS_An21d, BPS_An28d, Starch_An14d, Starch_An21d, and Starch_An28d. The chemical profiles of these crude extracts, along with those obtained from banana peels, were established by using LCMS data. The LCMS profile of the starch-enriched medium without inoculation was similar to that of banana peels. However, this profile showed low sensitivity due to the low concentration of metabolites.

### 3.2 Statistical Analysis

To perform a comprehensive comparison correlating all variables and determining the differences between culture media and cultivation times, as well as identifying the main metabolites responsible for this variation, multivariate analyses were applied. Data obtained from the positive and negative ionization modes were used separately for the comparison.

Principal component analysis (PCA) revealed significant differences between the chemical profiles of the samples, forming clusters, as depicted in Figure 2. The first two principal components (PC1 and PC2) explained 65% and 76.7% of the total variance of the data obtained from the positive and negative mode analyses, respectively. These PC values best represent the correlation between the groups.

**Figure 2:**
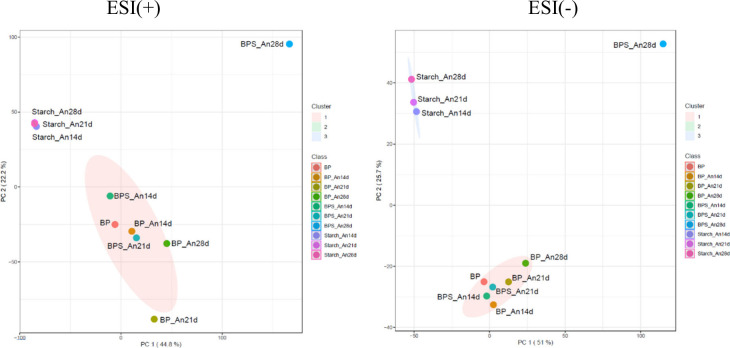
PCA plot with metabolites identified in the extracts obtained from the cultivation of the fungus *A. niger* in BP, BPS, and starch for 14, 21, and 28 days of growth in positive (ESI+) and negative (ESI−) ionization modes.

The PCA plot in positive mode analysis showed no proximity of BP_An21d to other samples. In contrast, it was grouped in a negative mode with BP and BPS samples except for BPS_An28d. This difference suggests that the BP_An21d sample contains metabolites that behave differently according to the ionization modes. Additionally, the BPS_An28d sample stood out for presenting a unique profile in both ionization modes. This sample was alone in the cluster, indicating a significantly different chemical composition profile. All samples from the starch medium formed a cluster in both ionization modes and were far from the BP and BPS samples.

The statistical analysis focused on a list of MS peaks and their corresponding areas ranked using *t* tests. From this analysis, the top 15 metabolites identified through the variable importance in projection (VIP) from partial least-squares discriminant analysis (PLS-DA) in both positive and negative modes were selected for correlation in the heatmap. The VIP plot produced by the PLS-DA models ranked metabolites based on their ability to differentiate between sample groups, including the biomass sample and those obtained from various fermentation times. The color scale (ranging from blue to red) indicates the relative amounts of each metabolite across various sample groups (red indicates a high quantity, and blue is not present). Previously identified metabolites are shown with their compound names, while additional metabolites are indicated by NI (Non Identified) followed by their precursor ion (*m*/*z*) found on the left side of the plot. The sample groups were based on fermentation media (starch and banana peels medium, BP; and banana peel medium enriched with starch, BPS) and duration (14, 21, and 28 d), are labeled at the bottom of the plot.

The VIP plot reveals nonadecan-2-amine (score >3) and the alkylaryl amine derivative, *m*/*z* 304.3005 (score >2.5) as the most concentrated compounds in the positive mode ionization (Figure 3). In contrast, it shows the fatty acid derivative (*m*/*z* 311.221) (score >1.8) and the monoglyceride disaccharide (*m*/*z* 559.3123) (score = 1.8) as the most prevalent in the negative mode ionization.

**Figure 3:**
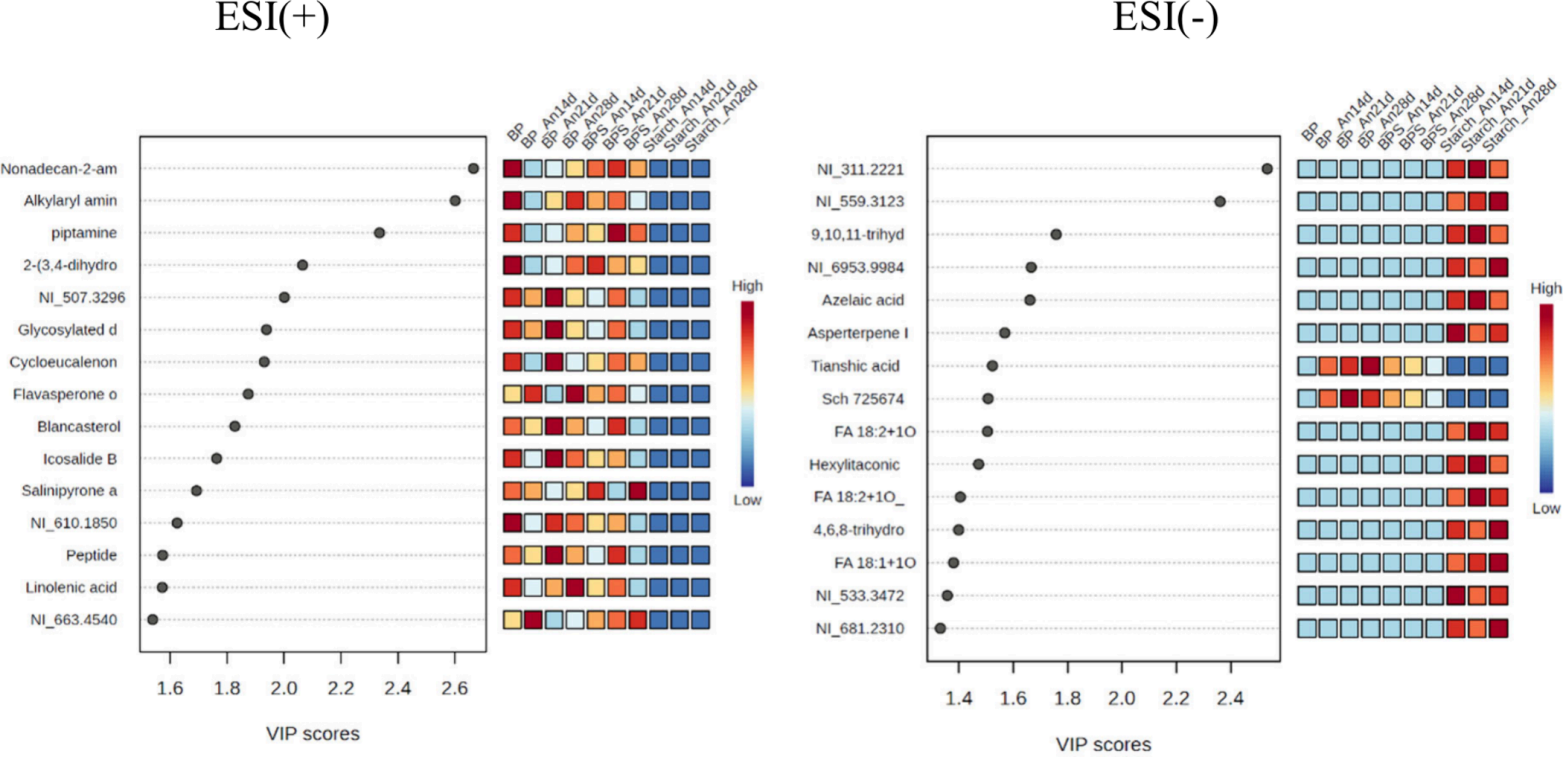
Fifteen most important metabolites from partial least squares discriminant analysis variable importance (PLS-DA VIP) from the cultivation of the fungus *A. niger* in BP, BPS, and starch at 14, 21, and 28 days of growth in positive (ESI+) and negative (ESI−) ionization modes.

The heatmap in Figure 4, displaying the top 60 metabolites based on the ANOVA *p*-value, was created using MetaboAnalyst 5.0 to forecast the distribution of metabolites during the fermentation timeline of 14, 21, and 28 days. In positive mode ionization, the chemical profile of BPS_An14d was close to that of banana peels (BP). Both were hierarchically close to BPS_An21d. The sequence of similarity in the hierarchy was followed by banana peel medium fermented for 28 days (BP_An28d), then BP_An14d, BP_An21d, BPS_An28d, Starch_An21d, Starch_An14d, and Starch_An28d. As observed in the PCA plot, fermentation periods of 21 to 28 days were suitable for *A. niger* to produce highly diverse secondary metabolites when grown on the banana peel and starch-enriched banana peel media, respectively. Moreover, a fermentation time of 28 days was the best for this fungus to produce more natural products.

**Figure 4:**
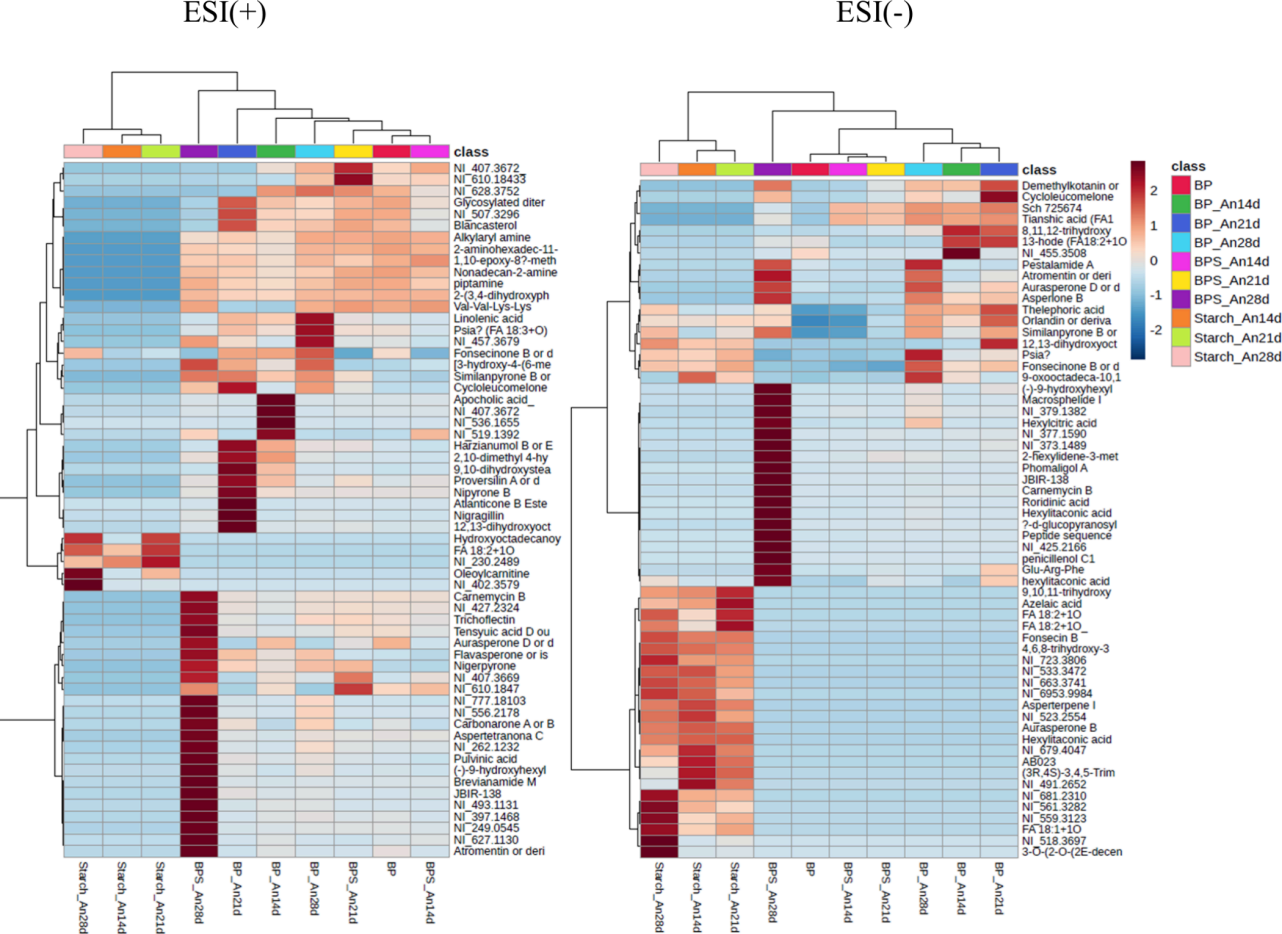
Heat map plot with 60 top metabolites identified in the extracts obtained from the cultivation of the fungus *A. niger* in BP, BPS, and starch at 14, 21, and 28 days of growth in positive (ESI+) and negative (ESI−) ionization mode.

The hierarchical distribution was different for the negative mode of ionization. Four groups were observed; among them, the first was composed of BP_An21d, BP_an14d, and BP_An28d. This group was close to BPS_An21d, BPS_An14d, and BP. Both clusters were close in hierarchy to BPS_An28d. The samples Starch_An21d, Starch_An14d, and Starch_An28d formed a group that was the end of the hierarchy. The present analysis also revealed that the fungus grown on media prepared with banana peels, produced in all periods, is a secondary metabolite sensitive to the negative ionization mode. However, a fermentation time of 28 days was optimal for significantly producing fungus-derived metabolites on the starch-enriched banana peel medium.

### 3.3 Dereplication of the Chemical Profiles

Crude extracts obtained from the fermentation, as well as those from banana peels and starch, were subjected to UPLC-ESI-QTOF-MS analysis. Generated mass spectrometry data (Figures S4–S9) were converted into a compatible format and then processed using MS-Dial, MS-FINDER, and GNPS.

The data obtained are compiled in Tables 1–3, in which the structures proposed by the aforementioned software and databases received scores from 0 to 10 (highest rate). Structure assignments without a score were possible using the dereplication technique in conjunction with previous studies reported in the literature. LCMS data from banana peels (BP) and samples from the fermentation of *A. niger* on banana peels for 14 (BP_An14d), 21 (BP_An21d), and 28 (BP_An28d) days revealed the presence of 76 secondary metabolites, among them, 10 substances were found in BP. The structures of banana peel metabolites were assigned as a polyol (*m*/*z* 261.1316), piptamine (*m*/*z* 332.3312), nonadecan-2-amine (*m*/*z* 284.3316), linolenic acid derivative (*m*/*z* 279.2327), isoorientin (*m*/*z* 471.1050), eriodyctiol-6-C-beta-d-glucopyranoside (*m*/*z* 473.1027), di­(-2-ethylhexyl)­phathalate (*m*/*z* 391.2848), icosalide B (*m*/*z* 685.4361), di­(2-ethylhexyl)­phthalate (*m*/*z* 413.2653), and cycloeucalenone (*m*/*z* 425.3781). In addition to secondary metabolites from banana peels, twenty-seven others were previously reported in *A. niger*,^30,31^ while 18 were formerly described in other *Aspergillus* species.^32,33^ Nigragillin was found at 1.63 min with a *m*/*z* of 223.1811, and the same mass value also appeared at 0.76 min, suggesting either a pyrazine or a piperazine derivative. The low fragmentation limits the consistent characterization of this secondary metabolite. However, pyrazines such as 2-methoxy-3,5-diisobutylpyrazine, 2-methoxy-3,6-di*sec*-butylpyrazine, and 2-methoxy-3,6-diisobutylpyrazine, with the same elemental composition, were obtained from the bacteria, *Carpophilus humeralis*.^34^ Pyranonigrin A (*m*/*z* 224.0557) together with other components, namely, phenoxyacetic acid (*m*/*z* 153.0553), cycloleucomelone (*m*/*z* 339.0503), atromentin (*m*/*z* 323.0550), carbonarone A (*m*/*z* 230.0819), pyrophen (*m*/*z* 310.1057), nigerpyrone (*m*/*z* 187.0759), hexylcitric acid (*m*/*z* 275.1126), orlandin (*m*/*z* 411.1079), pestalamide A (*m*/*z* 344.1132), demethylkotanin (*m*/*z* 425.1232), aspernigrin B (*m*/*z* 457.1759), hexylitaconic acid (*m*/*z* 213.1126), nigerasperone C (*m*/*z* 575.1550), kotanin and its isomer (*m*/*z* 439.1388), aurasperone D (*m*/*z* 555.1280), flavasperone (*m*/*z* 287.0920), fonsecinone B (*m*/*z* 589.1706), nipyrone (*m*/*z* 261.1464), and aurasperone A (*m*/*z* 571.1605) previously identified in *A. niger* were also found in this study.^35,36^ Most of the naphtho-γ-pyrones were detected alongside their positional or functional isomers (Table 1).

**Table 1:** Identification of Metabolites Using MS-FINDER Software and UPLC-ESI-HRMS Analysis in Positive and Negative Modes for Extracts Obtained from the Fungus *A. niger* Grown on Banana Peel (BP), at 14, 21, and 28 Days^a^

no.	*t*_R_ (min)	Formula	Adduct type	Precursor *m*/*z*	Error (ppm)	Proposed identification	Score	Class	Time (day)	Specie	Reference
1	0.76	C_13_H_22_N_2_O	[M + H]^+^	223.1811	–2.30	Nigragillin isomer	5.90	pyrazine	21	NI	
2	0.77	C_10_H_22_O_6_	[M + Na]^+^	261.1316	–0.03	NI	-	polyol	BP	NI	-
3	1.63	C_13_H_22_N_2_O	[M + H]^+^	223.1811	–0.95	Nigragillin	5.92	piperazine	14, 21	*A. niger*	^31^
4	2.21	C_10_H_9_NO_5_	[M + H]^+^	224.0557	–1.57	Pyranonigrin A	6.26	Pyranopyrrole	28	*A. niger*	^85^
			[M + H]^−^	222.0399	–1.56						
5	2.67	C_13_H_22_N_2_O_2_	[M + H]^+^	239.1758	–1.66	Ochramide B	<5	Pyrazine	14, 21	*A. ochraceus*	^86^
6	2.80	C_8_H_8_O_3_	[M + H]^+^	153.0553	–4.47	Phenoxyacetic acid or derivatives	5.58	Phenoxyacetic acid derivative	28	*A. niger*	^87^
7	4.98	C_18_H_10_O_7_	[M + H]^+^	339.0503	–1.10	Cycloleucomelone	6.19	Benzofurans	21, 28	*A. niger*	^30^
			[M-H]^−^	337.0338	4.66						
8	5.04	C_18_H_12_O_6_	[M-H]^−^	323.0550	3.43	Atromentin or derivatives	7.14	P-benzoquinones	28	*A. niger*	^36^
9	5.04	C_13_H_11_NO_3_	[M + H]^+^	230.0819	–2.75	Carbonarone A or B	6.21	Pyridine carboxaldehyde	21, 28	*A. niger*	^88,89^
10	5.05	C_11_H_10_O_4_	[M + H]^+^	207.0657	–2.50	Similanpyrone B	6.78	Isocoumarins	14, 21, 28	*A. similanensis*	^32^
			[M-H]^−^	205.0496	3.08		7.06				
11	5.05	C_16_H_17_NO_4_	[M + Na]^+^	310.1057	–2.51	Pyrophen	<5	Kavalactone	14, 21, 28	*A. niger*	^90^
12	5.06	C_12_H_10_O_2_	[M + H]^+^	187.0759	–2.92	Nigerpyrone	-	Polyketide	14, 21, 28	*A. niger*	^91^
13	5.24	C_12_H_20_O_7_	[M-H]^−^	275.1126	–1.73	Hexylcitric acid	-	alkylcitric acids	28	*A. niger*	^92^
14	5.30	C_22_H_18_O_8_	[M + H]^+^	411.1079	–1.112.17	Orlandin or derivatives	7.28	bicoumarin	14, 21, 28	*A. niger*	^36^
			[M-H]^−^	409.0920	2.17						
15	5.47	C_18_H_8_O_8_	[M-H]^−^	351.0136	2.96	Thelephoric acid	5.52	Terphenyl	14, 21, 28	mushroom *Polyozellus multiplex*	^38^
16	5.48	C_13_H_20_O_2_	[M + H]^+^	209.1541	–2.37	2,10-dimethyl 4-hydroxy-6-oxo-4-undecen-7-yne	5.44	Enone	14, 21	*A. ochraceus*	^32^
17	5.58	C_12_H_17_NO	[M + H]^+^	192.1386	–1.62	2-Methyl-N-(2-phenylethyl)propanamide	8.01	Alkamide	14, 21	NI	-
18	5.60	C_13_H_22_O_2_	[M + H]^+^	211.1697	–2.11	Harzianumol B or E	<5	Fatty alcohols	14, 21	*Trichoderma harzianum*	^40^
19	6.07	C_22_H_18_O_9_	[M + H]^+^	427.1027	–0.80	Maggiemycin	7.23	Tetracenequinones	28	*Aspergillus sydowii + Bacillus subtilis*	^93^
20	6.15	C_20_H_12_O_8_	[M + H]^+^	381.0604	0.25	Asperlone B	5.405.79	Dinaphthalenone	14, 21, 28	*Aspergillus sp.*	^94^
			[M-H]^−^	379.0450	2.48						
21	6.316.33	C_18_H_17_NO_6_	[M + H]^+^	344.1132	–0.98	Pestalamide A	7.216.88	Pyridone	28	*A. niger*	^89^
			[M-H]^−^	342.0970	3.82						
22	6.31	C_13_H_19_NO	[M + H]^+^	206.1546	–3.21	2-Methyl-N-(2-phenylethyl)butanamide	6.84	Alkamide	14, 21	Bacteria*Streptomyces sp*	^95^
23	6.33	C_23_H_20_O_8_	[M + H]^+^	425.1232	–0.25	Isokotanin B	6.59	bicoumarin	14, 28	*A. niger*	^96^
			[M-H]^−^	423.1071	–2.11						
24	6.33	C_13_H_11_NO_3_	[M-H]^−^	228.0656	4.44	Carbonarone A or B	<5	Pyranones and derivative	28	*A. niger*	^88,89^
25	6.50	C_23_H_14_O_9_	[M + H]^+^	435.0708	0.60	[3-hydroxy-4-(6-methoxy-5,8-dioxonaphthalen-2-yl)-5-oxofuran-2-ylidene](4-hydroxyphenyl)acetic acid	6.55	Naphthoquinones	21, 28	mushroom*Xerocomus badius*	^39^
26	6.62	C_18_H_32_O_5_	[M + Na]^+^	351.2143	–1.26	Sch725674	6.60	Macrolide	14, 21, 28	*Aspergillus sp.*	^97^
			[M-H]^−^	327.2163	4.26						
27	6.64	C_18_H_30_O_3_	[M+H^+^	293.2116	–0.24	PsiAalfa	-	Lacton	14, 21, 28	*A. nidulans.*	^37^
28	6.74	C_27_H_24_N_2_O_5_	[M + H]^+^	457.1759	–0.22	Aspernigrin B	6.90	Lactam	14, 21	*A. niger*	^91^
29	6.83	C_11_H_18_O_4_	[M-H]^−^	213.1126	2.95	hexylitaconic acid	7.93	Fatty acid	21, 28	*A. niger*	^92^
30	7.06	C_18_H_34_O_5_	[M + Na]^+^	353.2297	–1.96	8,11,12-trihydroxyoctadec-9-enoic acid derivative	-	Fatty acid	21, 28	*Aspergillus sp.*	^98^
31	7.06	C_14_H_20_O_3_	[M + Na]^+^	259.1312	0.72	Asperfuranone A	-	polyketide	14, 21	*Aspergillus sp.*	^99^
32	7.29	C_18_H_32_O_3_	[M + H]^+^	297.2428	–1.28	PsiAβ	5.5	lacton	21	*A. nidulans*	^36^
33	7.29	C_18_H_34_O_4_	[M + Na]^+^	337.2350	–1.423.92	12,13-dihydroxyoctadec-9-enoic acid derivative	-	Fatty acid	21	*Aspergillus sp.*	^97^
			[M-H]^−^	313.2372	3.92						
**3**4	7.35	C_31_H_26_O_11_	[M + H]^+^	575.1550	–0.37	Isoaurasperone F or Nigerasperone C	6.16	Naphtho-γ-pyrone	14, 21, 28	*A. niger*	^36^
35	7.53	C_14_H_25_NO_3_	[M + H]^+^	256.1912	–1.88	NI	5.91	Alkaloid	21	NI	-
36	7.57	C_13_H_18_O	[M + H]^+^	191.1437	–3.46	β-Damascenone	5.05	Enone	21, 28	*Musa sp.*	^100^
37	7.57	C_24_H_22_O_8_	[M + H]^+^	439.1388	–0.13	Kotanin	7.29	Bicoumarin	14, 21, 28	*A. niger*	^36^
38	7.63	C_24_H_22_O_8_	[M + H]^+^	439.1393	–0.36	Kotanin isomer	7.33	Bicoumarin	14, 21, 28	*A. niger*	^36^
39	7.64	C_18_H_34_O_4_	[M-H]^−^	313.2373	3.61	8,11,12-trihydroxyoctadec-9-enoic acid derivative	6.17	Fatty acid	14, 21	*Aspergillus sp.*	^98^
40	7.71	C_31_H_24_O_10_	[M-H]^−^	555.1280	3.0	Aurasperone D or derivative	-	Naphtho-γ-pyrone	14, 21, 28	*A. niger*	^36^
41	7.80	C_15_H_22_O_3_	[M + Na]^+^	273.1468	0.50	Proversilin A	-	Sesquiterpene	14, 21	*Aspergillus sp.*	^101^
42	7.80	C_16_H_14_O_5_	[M + H]^+^	287.0920	–2.10	Flavasperone	7.25	Naphtho-γ-pyrone	14, 21, 28	*A. niger*	^36^
43	7.98	C_18_H_36_O_4_	[M + Na]^+^	339.2511	1.64	9,10-dihydroxystearic acid derivative	7.55	Fatty acid	14, 21	*Aspergillus sp.*	^97^
			[M-H]^−^	315.2531	3.11		7.21				
44	8.00	C_18_H_30_O	[M + H]^+^	263.2376	–2.51	Norditerpene	-	Norditerpene	14, 21	*NI*	^–^
45	8.00	C_46_H_64_O_2_	[M + Na]^+^	671.4774	–4.47	Menaquinone 7	-	Meroterpenoid	21	*Bacteria Bacillus sp.*	^43^
46	8.00	C_18_H_34_O_3_	[M + H]^+^	299.2588	–2.44	6-keto stearic acid derivative	5.21	Fatty acid	14, 21	*Aspergillus sp.*	^102^
47	8.13	C_16_H_14_O_5_	[M + H]^+^	287.0922	–2.80	Flavasperone isomer	7.79	Naphtho-γ-pyrone	14	*A. niger*	^36^
48	8.20	C_32_H_28_O_11_	[M + H]^+^	589.1706	–0.28	Fonsecinone B	6.36	Naphtho-γ-pyrone	14, 21, 28	*A. niger*	^36^
49	8.20	C_31_H_24_O_10_	[M + Na]^+^	579.1260	0.30	Aurasperone d isomer	5.16	Naphtho-γ-pyrone	14, 21, 28	*A. niger*	^36^
			[M-H]^−^	555.1282	–1.66						
50	8.27	C_14_H_22_O_3_	[M + Na]^+^	261.1464	–1.20	Nipyrone B	5.14	α-pyrone derivative	21	*A. niger*	^103^
51	8.27	C_32_H_28_O_11_	[M + H]^+^	589.1703	0.24	Fonsecinone B isomers	6.06.63	Naphtho-γ-pyrone	14, 21, 28	*A. niger*	^36^
			[M-H]^−^	587.1542	2.87						
52	8.86	C_32_H_26_O_10_	[M + H]^+^	571.1605	–1.10	Aurasperone A	5.99	Naphtho-γ-pyrone	21, 28	*A. niger*	^36^
53	9.10	C_18_H_30_O_3_	[M+H–H_2_O]^+^	277.2167	–1.684.14	Psiaα	6.52	Fatty alcohol	14, 21, 28	*A. nidulans*	^37^
			[M-H]^−^	293.2110	4.14						
54	9.34	C_32_H_26_O_10_	[M + H]^+^	571.1608	–1.98	Aurasperone A isomer	5.6	Naphtho-γ-pyrone	21, 28	*A. niger*	^36^
55	9.35	C_18_H_28_O_3_	[M-H]^−^	291.1952	4.68	9-oxooctadeca-10,12,15-trienoic acid derivative	5.17	Fatty acid	14, 28	*Aspergillus sp.*	^97^
56	9.74	C_18_H_30_O_2_	[M + H]^+^	279.2324	–1.95	Linolenic acid	7.39	Fatty acid	14, 21, 28	*Aspergillus sp.*	^97^
57	10.23	C_23_H_41_N	[M + H]^+^	332.3312	–0.07	Piptamine derivative	8.15	alkylamine	BP, 14, 21, 28	NI	^–^
58	10.24	C_19_H_41_N	[M + H]^+^	284.3316	–1.49	Nonadecan-2-amine derivative	6.51	alkylamine	BP, 14, 21, 28	NI	^–^
59	10.38	C_30_H_48_O_3_	[M + H]^+^	457.3679	–0.61	Triterpene	**7.53**	Triterpenoid	28	NI	^–^
60	10.50	C_30_H_48_O	[M + H]^+^	425.3776	0.45	4α,14α-dimethyl-24-methylene-cholest-7,9(11)-dien-3β-ol	7.15	Triterpene	14	NI	^–^
61	10.90	C_17_H_24_O_4_	[M + H]^+^	315.1574	0.54	Salinipyrone A	5.40	polyketides	14, 28	Bacteria*Salinispora pacifica*	^42^
62	11.29	C_20_H_34_O_8_	[M + H]^+^	403.2326	0.11	Botcinic acid derivative	7.12	Polyketide	28	*Botrytis cinerea*	^40^
			[M + Na]^+^	425.2150							
63	11.87	C_18_H_30_O_2_	[M + H]^+^	279.2327	–1.23	Linolenic acid derivative	6.89	Fatty acid	BP, 21, 28	*Musa sp.*	^8^
64	12.28	C_25_H_20_O_11_	[M + Na]^+^	471.1050	–1.25	Isoorientin	-	Flavonoid	BP, 14, 21, 28	*Pueraria tuberosa*	^104^
65	12.28	C_21_H_22_O_11_	[M + Na]^+^	473.1027	–1.3	Eriodictyol 6-C-β-d-glucopyranoside	-	Flavonoid	BP, 14, 21, 28	*Aspalathus linearis*	^105^
66	14.38	C_30_H_48_O_3_	[M + H]^+^	457.3662	3.33	Triterpene	5.05	Triterpenoid	14, 21	NI	^–^
			[M-H]^−^	455.3508	–3.78						
67	14.44	C_33_H_54_O_5_	[M + H]^+^	531.4075	4.80	Related to Atlanticone B Esters	-	sesquitepene	21	*Lactarius atlanticus*	^106^
68	14.44	C_35_H_71_NO_3_	[M + H]^+^	554.5506	0.13	*N-*[2-Hydroxy-1-(hydroxymethyl)octadecyl]hexadecanamide	5.82	ceramide	21	*Aspergillus sp.*	^107^
69	14.96	C_24_H_38_O_4_	[M + H]^+^	391.2849	–1.57–0.43	Apocholic acid	5.64	Steroid	14	NI	^–^
			[M + Na]^+^	413.2664	–0.43						
70	15.12	C_22_H_43_NO	[M + H]^+^	338.3422	–1.34	Erucamide	5.84	Alkamide	28	*Bacillus megaterium L2*	^108^
71	15.12	C_30_H_48_O	[M + H]^+^	425.3781	–0.49	4α,14α-dimethyl-24-methylene-cholest-7,9(11)-dien-3β-ol isomer	7.67	Triterpenoid	28	*NI*	^–^
72	15.18	C_24_H_38_O_4_	[M + H]^+^	391.2848	–0.09	di-(2-ethylhexyl)phthalate	5.39	Phthalate	BP, 14	*Bacillus subtilis, Penicillium olsonii and Capparis spinosa*	^108–111^
73	15.29	C_34_H_60_N_4_O_10_	[M + H]^+^	685.4361	3.10	Icosalide B	6.45	Cyclic depsipeptide	BP, 21, 28	*Aureobasidium sp*	^112^
74	15.29	C_22_H_43_NO	[M + H]^+^	338.3421	–1.06	Erucamide isomer	5.28	Alkamide	14	*Bacillus megaterium*	^108^
75	15.29	C_24_H_38_O_4_	[M + Na]^+^	413.2653	–3.95	di-(2-ethylhexyl)phathalate isomer	-	Phthalate	BP, 21	*Bacillus subtilis, Penicillium olsonii and Capparis spinosa*	^109–111^
76	15.29	C_30_H_48_O	[M + H]^+^	425.3781	–0.49	Cycloeucalenone	8.01	Triterpenoid	BP, 21	*Musa sp*	^113^

a BP: compound identified in the banana peel biomass; NI: not identified.

Part of the other metabolites produced by *A. niger* in this study were previously reported in other *Aspergillus* species. Ochramide B, 2,10-dimethyl 4-hydroxy-6-oxo-4-undecen-7-yne (*m*/*z* 209.1541), similanpyrone B (*m*/*z* 207.0657), a pyrazine, a β-hydroxyenone, and an isocoumarin, produced respectively by *A. ochraceus* ^31,32^ and *A. similanensis*,^33^ were detected in the present study. Moreover, PsiAβ (*m*/*z* 297.2428) and PsiAα (*m*/*z* 293.2116), previously obtained from *A. nidulans,* were also detected in this study.^37^ Other secondary metabolites, including asperlone B, Sch725674 (*m*/*z* 351.2143), asperfuranone A (*m*/*z* 259.1312), a 12,13-dihydroxyoctadec-9-enoic acid derivative (*m*/*z* 337.2350), 8,11,12-trihydroxyoctadec-9-enoic acid derivative (*m*/*z* 313.2373), proversilin A (*m*/*z* 273.1468), 9,10-dihydroxystearic acid derivative (*m*/*z* 339.2511), 6-keto stearic acid derivative (*m*/*z* 299.2588), 9-oxooctadeca-10,12,15-trienoic acid derivative (*m*/*z* 291.1952), linolenic acid (*m*/*z* 279.2324), and *N*-[2-Hydroxy-1-(hydroxymethyl)­octadecyl]­hexadecanamide (*m*/*z* 554.5506) formerly identified in a partially sequenced *Aspergillus* were also characterized.

Based on literature searches, two of the annotated compounds, thelephoric acid and 3-hydroxy-4-(6-methoxy-5,8-dioxonaphthalen-2-yl)-5-oxofuran-2-ylidene]­(4-hydroxyphenyl)­acetic acid, were previously identified in the mushrooms, *Polyozellus multiplex* ^38^ and *Xerocomus badius*,^39^ respectively. This study also detected botcinic acid and a harzianumol derivative, previously identified in the fungi *Botrytis cinerea* and *Trichoderma harzianum*, respectively.^40,41^ In addition, menaquinone 7 and salinipyrone A, two bacteria-derived metabolites,^42,43^ were detected in the *A. niger* profile.

Table 1 also displays metabolites that were not described in any microorganisms, which include 2-methyl-*N*-(2-phenylethyl)­propanamide (*m*/*z* 192.1386), 2-methyl-*N*-(2-phenylethyl)­butanamide (*m*/*z* 206.1546), 8,11,12-trihydroxyoctadec-9-enoic acid derivative (*m*/*z* 353.2297), a nor-diterpene (*m*/*z* 263.2376), two isomers of 4α,14α-dimethyl-24-methylene-cholest-7,9(11)-dien-3β-ol (*m*/*z* 425.3776), two isomeric triterpenes (*m*/*z* 457.3662), apocholic acid (*m*/*z* 391.2849), and *cis*/*trans*-erucamide derivatives (*m*/*z* 338.3422).

The structure assignment of part of the proposed compounds was checked to sustain the MS-FINDER proposal by using the reported fragmentation pattern^44^ of ions listed in Tables S1 and S2. Schemes S1–S6 display the structure of fragment ions that led us to the chemical profile in Tables 1 and 2.

**Table 2:** Identification of Metabolites Using MS-FINDER Software and UPLC-ESI-HRMS Analysis in Positive and Negative Modes for Extracts Obtained from the Fungus *A. niger* Grown on Starch-Enriched Banana Peel Medium (BPS), at 14, 21, and 28 Days

no.	*t*_R_ (min)	Formula	Adduct type	Precursor*m*/*z*	Mass error	Metabolite name	Score	Class	Time (day)	Species	Reference
1	0.53	C_14_H_19_NO_5_	[M + H]^+^	282.1344	–2.85	Harzianopyridone	6.44	pyridone	28	*Trichoderma harzianum*	^52^
2	0.73	C_16_H_23_NO_5_	[M + H]^+^	310.1651	–0.65	NI	–	alkaloid	28	NI	–
3[Table-fn t2fn1]	0.77	C_10_H_22_O_6_	[M + Na]^+^	261.1316	–0.03	NI	–	polyol	BP	NI	–
4[Table-fn t2fn1]	2.23	C_10_H_9_NO_5_	[M + H]^+^	224.0561	–3.37	Pyranonigrin A	6.26	Pyranones and derivative	28	*A. niger*	^85^
			[M-H]^−^	222.0398	4.47		5.23				
5	3.68	C_11_H_16_O_6_	[M-H]^−^	243.0867	2.92	Tensyuic acid A or F	6.6	Fatty diacid	28	*A. niger*	^114^
6	3.76	C_11_H_18_O_5_	[M + Na]^+^	253.1052	–2.41	9-hydroxyhexylitaconic acid	7.47	Fatty diacid	28	*A. aculeatus*	^46^
			[M-H]^−^	229.1070	2.30						
7	3.77	C_11_H_16_O_4_	[M + H]^+^	213.1128	–3.13	Aspergilactone A	6.29	Butenolide	28	*Aspergillus sp.*	^36^
8	4.43	C_16_H_25_NO_4_	[M-H]^−^	294.1698	4.34	Penicillenol C1	5.0	*N*-alkylpyrrolidine	28	*A. restrictum*	^50^
9	4.56	C_16_H_23_NO_4_	[M + H]^+^	294.1708	–2.44	NI	–	Alkaloid	28	NI	–
10	4.60	C_22_H_16_O_9_	[M + H]^+^	425.0866	0.26	1,2-diacetoxy-3-(4′-hydroxyphenyl)-4,7,8-trihydroxydibenzofuran	5.81	Terphenyl derivative	28	mushroom*Sarcodon laevigatum*	^53^
11	4.60	C_21_H_20_O_10_	[M + Na]^+^	455.0969	3.26	purpurquinone A	–	polyketide	28	*Aspergillus sp.*	^90^
12	4.864.95	C_14_H_22_O_7_	[M + Na]^+^	325.1264	–2.07	Roridinic acid	5.58	Fatty acid	28	*Cordyceps sp*	^54^
			[M-H]^−^	301.1281	3.89						
13	4.86	C_26_H_38_N_6_O_12_	[M-H]^−^	625.2447	–3.59	Peptide	–	Peptide	28	*A. niger*	^115^
14	4.95	C_18_H_15_N_3_O_3_	[M + H]^+^	322.1180	1.92	Brevianamide M	5.19	Quinazoline	28	*A. versicolor*	^48^
15[Table-fn t2fn1]	5.04	C_18_H_12_O_6_	[M-H]^−^	323.0550	3.43	Atromentin	7.38	P-benzoquinone	28	*A. niger*	^36^
16[Table-fn t2fn1]	5.06	C_12_H_10_O_2_	[M + H]^+^	187.0762	–4.54	Nigerpyrone	–	polyketide	21, 28	*A. niger*	^91^
17[Table-fn t2fn1]	5.09	C_11_H_10_O_4_	[M + H]^+^	207.0657	–0.16	Similanpyrone B or derivatives	6.95	Isocoumarins	21,28	*A. similanensis*	^33^
			[M-H]^−^	205.0501	3.07						
18	5.09	C_14_H_20_O_6_	[M+HCO_2_]^−^	329.1231	–1.65	Phomaligol A	–	polyketide	28	*Aspergillus sp.*	^116^
19[Table-fn t2fn1]	5.11	C_13_H_11_NO_3_	[M + H]^+^	230.0820	–3.62	Carbonarone A or B	6.99	g-pyrone	28	*A. niger*	^88,89^
20	5.11	C_16_H_8_O_3_	[M + H]^+^	249.0545	0.49	NI	–	Polyketide	28	NI	–
21[Table-fn t2fn1]	5.11	C_16_H_17_NO_4_	[M + Na]^+^	310.1048	–2.35	Pyrophen		Pyrone	21, 28	*A. niger*	^90^
22[Table-fn t2fn1]	5.11	C_18_H_12_O_6_	[M + H]^+^	325.0709	–0.96	Atromentin derivatives	–	P-benzoquinones	28	*A. niger*	^36^
23	5.11	C_15_H_22_O_8_	[M + Na]^+^	353.1208	–1.24	NI	–	polyketide	28	NI	–
24	5.11	C_31_H_24_O_13_	[M + Na]^+^	627.1130	2.46	NI	–	Polyketide	28	NI	–
25[Table-fn t2fn1]	5.21	C_12_H_20_O_7_	[M-H]^−^	275.1128	2.99	Hexylcitric acid	–	Acids	28	*A. niger*	^92^
26[Table-fn t2fn1]	5.30	C_22_H_18_O_8_	[M + H]^+^	411.1076	–0.38	Orlandin or isomer	5.365.96	coumarin	21, 28	*A. niger*	^36^
			[M-H]^−^	409.0917	2.90						
27	5.36	C_19_H_24_O_8_	[M-H]^−^	379.1382	–2.88	NI	–	polyketide	28	NI	–
28	5.48	C_20_H_26_O_8_	[M-H]^−^	393.1539	4.04	Hamigeromycin A or C	7.20	Zearalenone	28	Fungus *Hamigera avellanea*	^55^
29	5.54	C_16_H_24_O_8_	[M + Na]^+^	367.1366	–0.76	Macrosphelide I	7.07	macrolide	28	Fungus *Periconia* *byssoides*	^56^
			[M-H]^−^	343.1387	3.32						
30[Table-fn t2fn1]	5.58	C_12_H_17_NO	[M + H]^+^	192.1386	–1.62	2-Methyl-N-(2-phenylethyl)propanamide	8.08	N-acyl amine	21	NI	–
31	5.64	C_17_H_26_O_9_	[M + Na]^+^	397.1468	–1.644.03	NI	–	NI	28	NI	–
			[M-H]^−^	373.1489							
32	5.78	C_18_H_15_NO	[M + H]^+^	262.1232	–2.14	NI		NI	28	NI	–
33	5.78	C_18_H_12_O_5_	[M + H]^+^	309.0762	–1.46	Pulvinic acid	5.61	Pulvinone	28	*A. neoniveu*	^47^
34	5.78	C_18_H_12_O_7_	[M + H]^+^	341.0660	–0.94	Atromentic acid	–	Pulvinone	28	*Boletus permagnificus*	^57^
35[Table-fn t2fn1]	6.05	C_22_H_18_O_9_	[M + H]^+^	427.1028	–1.04	Maggiemycin	7.23	Tetracenequinones	28	*Aspergillus sp. + Bacillus subtilis*	^93^
36	6.05	C_24_H_20_O_8_	[M + H]^+^	437.1229	0.45	inoscavin B	–	Styrylpyrone	28	Mushroom *Inonotus xeranticus*	^117^
37	6.07	C_21_H_32_O_7_	[M-HCO_2_]^−^	441.2113	–2.62	β-d-glucopyranosyl aspergillusene A	–	Sesquiterpene	28	*Aspergillus sp.*	^118^
38[Table-fn t2fn1]	6.19	C_20_H_12_O_8_	[M-H]^−^	379.0445	3.79	Asperlone B	5.93	Dinaphthalenone	28	*Aspergillus sp.*	^94^
39	6.29	C_17_H_17_NO_4_	[M + Na]^+^	322.1054	–1.41	NI	–	Alkaloid	28	NI	–
40	6.296.54	C_19_H_24_O_7_	[M + Na]^+^	387.1415	–0.213.09	JBIR-138	6.80	Sesquiterpene esters	28	*Aspergillus sp.*	^119^
			[M-H]^−^	363.1438							
41	6.29	C_22_H_28_O_8_	[M + H]^+^	421.1856	0.46	Aspertetranone C	5.11	Naphthopyranones	28	*Aspergillus sp.*	^89^
42	6.29	C_27_H_40_O_10_	[M + H]^+^	525.2720	–4.91	NI	–	–	28	NI	–
43[Table-fn t2fn1]	6.31	C_13_H_19_NO	[M + H]^+^	206.1547	–3.70	2-Methyl-N-(2-phenylethyl)butanamide	6.80	Alkamide	21, 28	*Bacteria Streptomyces sp*	^95^
44[Table-fn t2fn1]	6.33	C_23_H_20_O_8_	[M + H]^+^	425.1232	–0.25	Demethylkotanin	6.59	bicoumarin	21, 28	*A. niger*	^96^
			[M-H]^−^	423.1071	–2.11						
45[Table-fn t2fn1]	6.38	C_13_H_11_NO_3_	[M-H]^−^	228.0661	2.26	Carbonarone A or B	5.29	g-pyrone	28	*A. niger*	^88,89^
46[Table-fn t2fn1]	6.38	C_18_H_17_NO_6_	[M-H]^−^	342.0973	2.95	Pestalamide A	6.58	Pyranone	28	*A. niger*	^89^
47[Table-fn t2fn1]	6.50	C_23_H_14_O_9_	[M + H]^+^	435.0708	0.60	[3-hydroxy-4-(6-methoxy-5,8-dioxonaphthalen-2-yl)-5-oxofuran-2-ylidene](4-hydroxyphenyl)acetic acid	6.55	pulvinone	28	mushroom*Xerocomus badius*	^39^
48	6.58	C_19_H_24_O_5_	[M-HCO_2_]^−^	377.1590	4.17	Asperfuranone	–	Polyketide	28	*A. nidulans*	^49^
49[Table-fn t2fn1]	6.61	C_18_H_32_O_5_	[M + Na]^+^	351.2143	–1.26	Sch725674	–	Macrolide	14, 21, 28	*Aspergillus sp.*	^97^
50[Table-fn t2fn1]	6.61	C_18_H_28_O_3_	[M + H]^+^	293.2114	–0.90	NI	–	NI	14, 21, 28	NI	–
51	6.68	C_13_H_20_O_6_	[M + Na]^+^	295.1158	–2.17	Tensyuic acid D or C	<5	Fatty diacid	28	*A. niger*	^114^
52	6.68	C_14_H_20_O_6_	[M + Na]^+^	307.1158	0.14	Pyrenocine L	–	Pyrone	28	Fungus*Phomopsis sp*	^58^
53[Table-fn t2fn1]	6.68	C_26_H_34_N_4_O_7_	[M + H]^+^	515.2485	2.96	Related to tyr-tryp-val-Ala	–	Peptide	28	NI	–
54	6.70	C_12_H_20_O_4_	[M-H]^−^	227.1280	3.87	2-hexylidene-3-methylsuccinic acid 4-methyl ester	5.76	Fatty diacid	28	Fungus*Halorosellinia oceanica*	^59^
55	6.81	C_20_H_30_N_6_O_6_	[M-H]^−^	449.2135	2.33	Related to Glu-Arg-Phe	–	Peptide	28	NI	–
56[Table-fn t2fn1]	6.83	C_11_H_18_O_4_	[M + Na]^+^	237.1102	–0.33	Hexylitaconic acid	7.98	Fatty diacid	28	*A. niger*	^92^
			[M-H]^−^	213.1127	2.49						
57	6.95	C_17_H_24_O_7_	[M + H]^+^	341.1616	–3.25	Aspergilloid E	–	Sesquiterpene	28	*A. flavus*	^51^
58	6.95	C_21_H_16_O_9_	[M + Na]^+^	435.0713	4.82	Mitorubrinic acid	–	Azaphilone	28	*Aspergillus sp*	^94^
59[Table-fn t2fn1]	7.08	C_18_H_32_O_4_	[M+H–H_2_O]^+^	295.2270	–0.73	10,11-dihydroxyoctadeca-8,12-dienoic acid	5.33	Fatty acid	14, 21	*A. flavus*	^120^
60[Table-fn t2fn1]	7.08	C_18_H_34_O_5_	[M + Na]^+^	353.2298	–1.68	8,11,12-trihydroxyoctadec-9-enoic acid derivative	–	Fatty acid	14, 21	*Aspergillus sp.*	^98^
61	7.31	C_33_H_34_O_13_	[M + H]^+^	639.2077	–0.75	NI	–	NI	28	NI	–
62	7.42	C_32_H_30_N_2_O_8_	[M + H]^+^	571.2076	–0.19	Aspernigrin C	6.69	Kavalactones	28	*A. niger*	^36^
63	7.49	C_16_H_35_NO_2_	[M + H]^+^	274.2744	–1.26	Hexadecasphinganine	<5	Sphingolipids	BP, 14, 21, 28	*Algae* *Ulva lactuca*	^121^
64[Table-fn t2fn1]	7.63	C_24_H_22_O_8_	[M + H]^+^	439.1391	–0.81	Kotanin	7.13	bicoumarin	28	*A. niger*	^36^
65[Table-fn t2fn1]	7.68	C_31_H_24_O_10_	[M-H]^−^	555.1273	–0.02	Aurasperone D or derivative	–	Naphtho-γ-pyrone	28	*A. niger*	^35^
66	7.97	C_29_H_33_NO_10_	[M + H]^+^	556.2178	–0.14	NI	–	NI	28	NI	–
67[Table-fn t2fn1]	7.80	C_16_H_14_O_5_	[M + H]^+^	287.0920	–2.10	Flavasperone or isomer	7.25	Naphtho-γ-pyrone	28	*A. niger*	^36^
68[Table-fn t2fn1]	8.13	C_16_H_14_O_5_	[M + H]^+^	287.0922	–2.80	Flavasperone or isomer	7.79	Naphtho-γ-pyrone	21	*A. niger*	^36^
69[Table-fn t2fn1]	8.13	C_31_H_24_O_10_	[M + H]^+^	557.1451	–1.58	Aurasperone D or derivative	5.8	Naphtho-γ-pyrone	21, 28	*A. niger*	^36^
70	8.19	C_23_H_32_O_9_	[M + H]^+^	453.2122	–0.64	Carnemycin B	5.26	Polyketides	28	*Aspergillus sp.*	^122^
			[M-H]^−^	451.1954	4.33		5.35				
71	8.37	C_13_H_10_O_5_	[2M+Na]^+^	493.1131	–0.36	myxotrichin C	–	Chromone	28	*Myxotrichum spp.*	^60^
72	8.48	C_22_H_34_O_8_	[M + H]^+^	427.2324	0.57	Colletobredin C	–	Polyketide	28	*Colletotrichum aotearoa*	^61^
			[M-H]^−^	425.2166	3.50						
72	8.48	C_33_H_60_O_6_	[M + Na]^+^	575.4262	–4.45	NI	–	NI	28	NI	–
74	8.48	C_39_H_32_O_15_	[M + Na]^+^	763.1649	1.32	NI	–	NI	28	NI	–
75	8.76	C_20_H_20_O_6_	[M + H]^+^	357.1336	–0.94	Herqueichrysin	–	phenalenone	28	*Penicillium herquei*	^62^
76[Table-fn t2fn1]	8.87	C_32_H_26_O_10_	[M + H]^+^	571.1599	–0.046	Aurasperone A or derivatives	5.44	Naphtho-γ-pyrone	21, 28	*A. niger*	^35^
77	8.99	C_40_H_34_O_15_	[M + Na]^+^	777.1810	1.88	NI	–		28	NI	–
78	9.24	C_21_H_37_N	[M + H]^+^	304.3005	–2.05	Alkylaryl amine	7.61	Amine	BP, 14, 21, 28	NI	–
79	9.54	C_35_H_30_O_11_	[M + H]^+^	627.1865	–0.66	Talaroketal A	6.98	phenalenone	28	Fungus*Talaromyces stipitatus*	^63^
80	10.27	C_16_H_22_O_4_	[M + H]^+^	279.1597	–2.21	1,10-epoxy-8α-methoxyermophilanolide	–	sesquiterpene	BP, 14, 21, 28	Plant*Senecio mairetianus*	^123^
81[Table-fn t2fn1]	10.51	C_30_H_48_O	[M + H]^+^	425.3783	–1.19	4α,14α-dimethyl-24-methylene-cholest-7,9(11)-dien-3β-ol	7.51	triterpene	21, 28	NI	–
82	10.58	C_17_H_14_O_5_	[M + H]^+^	299.0920	–2.01	Trichoflectin	6.23	Azaphilone	21, 28	fungus*Trichopezizella nidulus*	^64^
83[Table-fn t2fn1]	10.89	C_17_H_24_O_4_	[M + Na]^+^	315.1571	–1.44	Salinipyrone A	5.40	polyketides	28	bacteria*Salinispora pacifica*	^45^
84[Table-fn t2fn1]	11.29	C_20_H_34_O_8_	[M + Na]^+^	425.2150	–1.02	Botcinic acid	7.12	Polyketide	14, 21, 28	Fungus*Botrytis cinerea*	^41^
85	11.29	C_24_H_48_N_6_O_6_	[M + H]^+^	517.3712	–0.75	NI	–	Peptide	BP, 14, 21, 28	NI	–
86	11.30	C_22_H_44_N_6_O_5_	[M + H]^+^	473.3453	0.20	Related to Val-Val-Lys-Lys	–	Peptide	BP, 14, 21, 28	NI	–
87[Table-fn t2fn1]	11.86	C_18_H_30_O_2_	[M + H]^+^	279.2322	–1.23	Linolenic acid	7.39	Fatty acid	BP, 21	*Musa sp.*	^8^
88	12.14	C_16_H_33_NO	[M + H]^+^	256.2641	–1.21	2-aminohexadec-11-en-3-ol	–	Sphingolipid	BP, 14, 21, 28	*Aspergillus sp.*	^107^
89[Table-fn t2fn1]	12.32	C_25_H_20_O_11_	[M + Na]^+^	471.1051	–1.03	isoorientin	–	Flavonoid	BP, 28	*Pueraria tuberosa*	^104^
90[Table-fn t2fn1]	12.32	C_21_H_22_O_11_	[M + Na]^+^	473.1027	–1.30	Eriodictyol 6-C-β-d-glucopyranoside		Flavonoid	BP, 14, 21, 28	*Aspalathus linearis*	^105^
91	15.09	C_16_H_26_	[M + H]^+^	219.2113	–2.62	NI	–	Sesquiterpene	21	NI	–
92	15.09	C_21_H_10_N_2_O_4_	[M + H]^+^	355.0702	3.20	13-Docosenamide	–	Fatty amide	21	*Penicillium chrysogenum*	^124^
93	15.09	C_10_H_16_O10	[2M+H]^+^	593.1578	2.16	veracylglucan A	–	Glycoside	21	*Aloe vera*	^125^
94[Table-fn t2fn1]	15.11	C_22_H_43_NO	[M + H]^+^	338.3420	–0.77	Erucamide	5.65	Alkamide	21	*Bacillus megaterium L2*	^108^
95[Table-fn t2fn1]	15.12	C_30_H_48_O	[M + H]^+^	425.3781	–0.49	4α,14α-dimethyl-24-methylene-cholest-7,9(11)-dien-3β-ol isomer	7.67	Triterpenoid	21	NI	–
96	15.18	C_21_H_32_O	[M + H]^+^	301.2529	–1.03	NI	–	Diterpene	14, 28	NI	–
97[Table-fn t2fn1]	15.18	C_24_H_38_O_4_	[M + H]^+^	391.2848	–0.09	di(2-ethylhexyl)phthalate	–	Phthalate	BP, 14, 21, 28	*Bacillus subtilis, Penicillium olsonii and Capparis spinosa*	^109–111^
98[Table-fn t2fn1]	15.18	C_22_H_43_NO	[M + H]^+^	338.3417	0.12	Isomer of erucamide	5.61	Alkamide	14, 28	*Bacillus megaterium L2*	^108^
99[Table-fn t2fn1]	15.29	C_30_H_48_O	[M + H]^+^	425.3781	–0.72	Cycloeucalenone	7.39	Triterpene	BP	*Musa sp.*	^113^

b Metabolites identified in both substrates, NI: not identified.

The peak at a retention time (tR) of 1.63 min with *m*/*z* 223.1811 [C_13_H_22_N_2_O + H]^+^ was assigned to the metabolite nigragilin. Its tandem mass spectrum displays *m*/*z* 129.1386 [C_7_H_17_N_2_ + H]^+^ corresponding to *N*-methylpiperazinium. This ion resulted from the loss of the side chain (94 Da). Another fragment at *m*/*z* 95.0494 [C_6_H_7_O]^+^ was assigned as an acylium ion, formed through α-cleavage of the C–N bond of the amide functionality and elimination of the *N*-methylpiperazine moiety.

The ion at tR 5.04 min with *m*/*z* 323.0550 [C_18_H_12_O_6_ – H]^−^ present only in BP_An28d and BPS_An28d was annotated as atromentin. Its MS^2^ spectrum showed the fragment ion *m*/*z* 295.0615 [C_17_H_12_O_5_ – H]^−^ yielded after the precursor lost CO (28 Da).

The peak at tR 6.15 min with *m*/*z* 379.0450 [C_20_H_12_O_8_ – H]^−^, present in the three cultivation times, was assigned as asperlone B. In its tandem mass spectrum, *m*/*z* 361.0362 [C_20_H_10_O_7_ – H]^−^ and 333.0400 [C_19_H_10_O_6_ – H]^−^ were observed, consistent with the loss of H_2_O (18 Da) and CO (28 Da), respectively.

The metabolite pestalamide A, known as an *A. niger-*derived compound, was annotated in positive mode with *m*/*z* 344.1132 [C_18_H_18_NO_6_ + H]^+^. Its MS^2^ spectrum showed a fragment *m*/*z* = 230.0826 [C_13_H_10_NO_3_ + H]^+^. This ion was produced after the precursor lost the 2-methylbutane-dioic acid moiety. The ion *m*/*z* 230.0826 in turn produced the lighter ions *m*/*z* 213.0548, 185.0512, and 157.0647 after eliminating NH_3_ (17 Da), NH_3_/CO (45 Da), and NH_3_/2CO (73 Da), respectively. In negative mode, the metabolite pestalamide A (*m*/*z* 342.0970) gave fragment ions *m*/*z* 228.0634 [C_13_H_11_NO_3_ – H]^−^ and *m*/*z* 185.0620 [C_12_H_10_O_2_ – H]^−^ in its MS/MS spectrum. These fragments corresponded to sequential elimination of the 2-methylbutane-dioic acid moiety and NH_3_ (17 Da), respectively.

The peak at tR 6.38 min with *m*/*z* 213.1126 [C_11_H_18_O_8_ – H]^−^ in negative mode was attributed to hexylitaconic acid. In its MS^2^ spectrum, the observed fragment *m*/*z* 169.1219 [C_10_H_18_O_2_ – H]^−^ differs from the precursor by CO_2_ (44 Da).

The peak at tR 7.98 min with *m*/*z* 339.2511 [C_18_H_36_O_4_ + Na]^+^ and 315.2531 [C_18_H_36_O_4_ – H]^−^, in positive and negative mode, respectively, was annotated as 9,10-dihydroxystearic acid. In the negative mode, the obtained fragment *m*/*z* 297.2421 [C_18_H_34_O_3_ – H]^−^ corresponded to the loss of H_2_O (18 Da).

Starch was added to the culture media as a carbohydrate source to enhance the fungal catabolism and metabolism. Out of this process, 99 secondary metabolites were detected and are reported in Table 2. Part of these compounds were plant metabolites and were annotated as hexadecasphinganine (*m*/*z* 274.2744), an alkylarylamine (*m*/*z* 304.3005), 1,10-epoxy-8alfa-methoxyvermophilanolide (*m*/*z* 279.1597), an isomer of Val-Val-Lys-Lys (*m*/*z* 473.3453), a peptide (*m*/*z* 517.3712), linolenic acid (*m*/*z* 279.2322), 2-aminohexadec-11-en-3-ol (*m*/*z* 256.2641) alongside isoorientin (*m*/*z* 471.1050), eriodyctiol-6-C-beta-d-glucopyranoside (*m*/*z* 473.1027), etalon (*m*/*z* 391.2848), and cycloeucalenone (*m*/*z* 425.3781). Part of the metabolites found exclusively in banana peel-derived media were not detected in those composed of starch-enriched banana peel. On the other hand, these starch-enriched media also yielded compounds that were not detected in media without starch. This discrepancy can be associated with microorganism biochemical activities that change according to the chemical properties of the culture medium.^45^ In this way, the medium chemical composition modulates different metabolism activities. Growing *A. niger* on starch-enriched banana peel media afforded 22 secondary metabolites previously identified in the same species; 19 additional compounds herein reported were already found in other *Aspergillus* species (with genomes either fully or partially identified). In addition, 25 of these fungus-derived metabolites were already described in mushrooms, bacteria, fungi, association fungi/bacteria, and algae species. Among the previously reported *A. niger* secondary metabolites, 18 were found in both media. However, tensyuic acid A (*m*/*z* 243.0867), the peptide (*m*/*z* 625.2447), aspernigrin C (*m*/*z* 571.2076), and tensyuic acid D (*m*/*z* 295.1158) were generated only in starch-enriched banana peel media.

In banana peel media, the biosynthesis of previously identified metabolites also occurred. These compounds included 9-hydroxyhexylitaconic acid (*m*/*z* 229.1070), penicillenol C1 (*m*/*z* 294.1698), brevianamide M (*m*/*z* 322.1180), pulvinic acid (*m*/*z* 309.0762), asperfuranone (*m*/*z* 377.1590), and aspergilloid E (*m*/*z* 341.1616) previously reported in *Aspergillus* species with the fully identified genome.^46–51^ Table 2 also displays substances previously found in *Aspergillus* species with partially identified genomes. Two of these compounds, namely, asperlone B (*m*/*z* 379.0445) and sch725674 (*m*/*z* 351.2143), were biosynthesized in both cultures, whereas aspergilactone A (*m*/*z* 213.1128), purpurquinone A (*m*/*z* 455.0969), phomoligol A (*m*/*z* 329.1231), β-d-glucopyranosyl aspergillusense A (*m*/*z* 441.2113), asperlone B (*m*/*z* 379.0445), JBIR-138 (*m*/*z* 363.1438), aspertetranone C (*m*/*z* 421.1856), mitorubrinic acid (*m*/*z* 435.0713), carnemycin B (*m*/*z* 451.1954), and 2-aminohexadec-11-en-3-ol (*m*/*z* 256.2641) were found only in starch-enriched media. These culture media also produced harzianopyridone (*m*/*z* 282.1344), which was previously identified in *Trichoderma harzianum*.^52^ In addition, 1,2-diacetoxy-3-(4′hydroxyphenyl)-4,7,8-trihydroxydibenzofuran (*m*/*z* 425.0866), roridinic acid (*m*/*z* 301.1281), hamigeromycin A (*m*/*z* 393.1539), and macrosphelide I (*m*/*z* 343.1387) were formerly identified in the mushroom *Sarcodon laevigatum* ^53^ and the fungi *Cordyceps* sp.,^54^ *Hamigera avellamea*,^55^ and *Periconia byssoides*.^56^ Atromentic acid (*m*/*z* 341.0660) from *Boletus permagnificus*,^57^ inoscavin B (*m*/*z* 437.1229) from *Innonotus xeranticus*, [3-hydroxy-4-(6-methoxy-5,8- dioxonaphthalen-2-yl)-5-oxofuran-2-ylidene]­(4-hydroxy phenyl)­acetic acid (*m*/*z* 435.0708) from *Xerocomus badius*,^39^ pyrenocine L (*m*/*z* 307.1158),^58^ 2-hexylidene-3-methylsuccinic acid – methyl ester (*m*/*z* 227.1280) from *Halorosellinia oceanica*,^59^ myxotrichin C (*m*/*z* 493.1131) from *Myxotrichum spp* ^60^ colletobredin C (*m*/*z* 427.2324) from *Colletotrichum Aotearoa*,^61^ herqueichrysin (*m*/*z* 357.1336) from *Penicillium herquei*,^62^ talaroketal A (*m*/*z* 627.1865) from *Talaromyces stipitatus*,^63^ trichoflectin (*m*/*z* 299.0920) from *Trichopezizella nidulus*,^64^ salinipyrone A (*m*/*z* 315.1571) from *Salinispora pacifica*,^42^ and botcinic acid (*m*/*z* 425.2150) from *Botrytis cinerea* ^41^ were also annotated in this second medium. The structures of 26 detected compounds could not be characterized.

The GNPS database was also used to annotate the metabolites and correlate those of the same classes using molecular networks. In this technique, each compound is represented by a node and each node contains a pizza diagram according to its proportion in different analyzed samples. The network connects substances that are stereoisomers, positional, and functional isomers.^28^

GNPS analysis of BP_An14d, BP_An21d, and BP_An28d detected 45 nodes; among them, 28 match substances in its database (Figure 5). In the positive mode ionization, the plant secondary metabolites isoorientin and eriodictyol-6-C-β-d-glucopyranoside form a cluster as both differ by one unsaturation. Although six bicoumarins were detected and identified (Tables 1 and 2), only kotanin, isokotanin B, and orlandin were detected in GNPS. These metabolites formed a cluster as they are structurally related and differ from one another by the number of methoxy groups. Two alkamides, including 2-methyl-*N*-(2-phenylethyl)­propanamide and 2-methyl-*N*-(2-phenylethyl)­butanamide, also formed a cluster, as the difference between both compounds is 14 Da (methyl group). Other compounds detected by GNPS did not form clusters, including those of the negative mode ionization. The MS-FINDER database proposed the structure of aurasperone A for *m*/*z* 571.1605, which was found in 21 and 28 days of fermentation. However, GNPS identified for the same mass value, a biflavonoid known as 4′,4′′′-di-O-methylisochamaejasmin. This database also revealed that *m*/*z* 571.1605 is present in banana peels.

**Figure 5:**
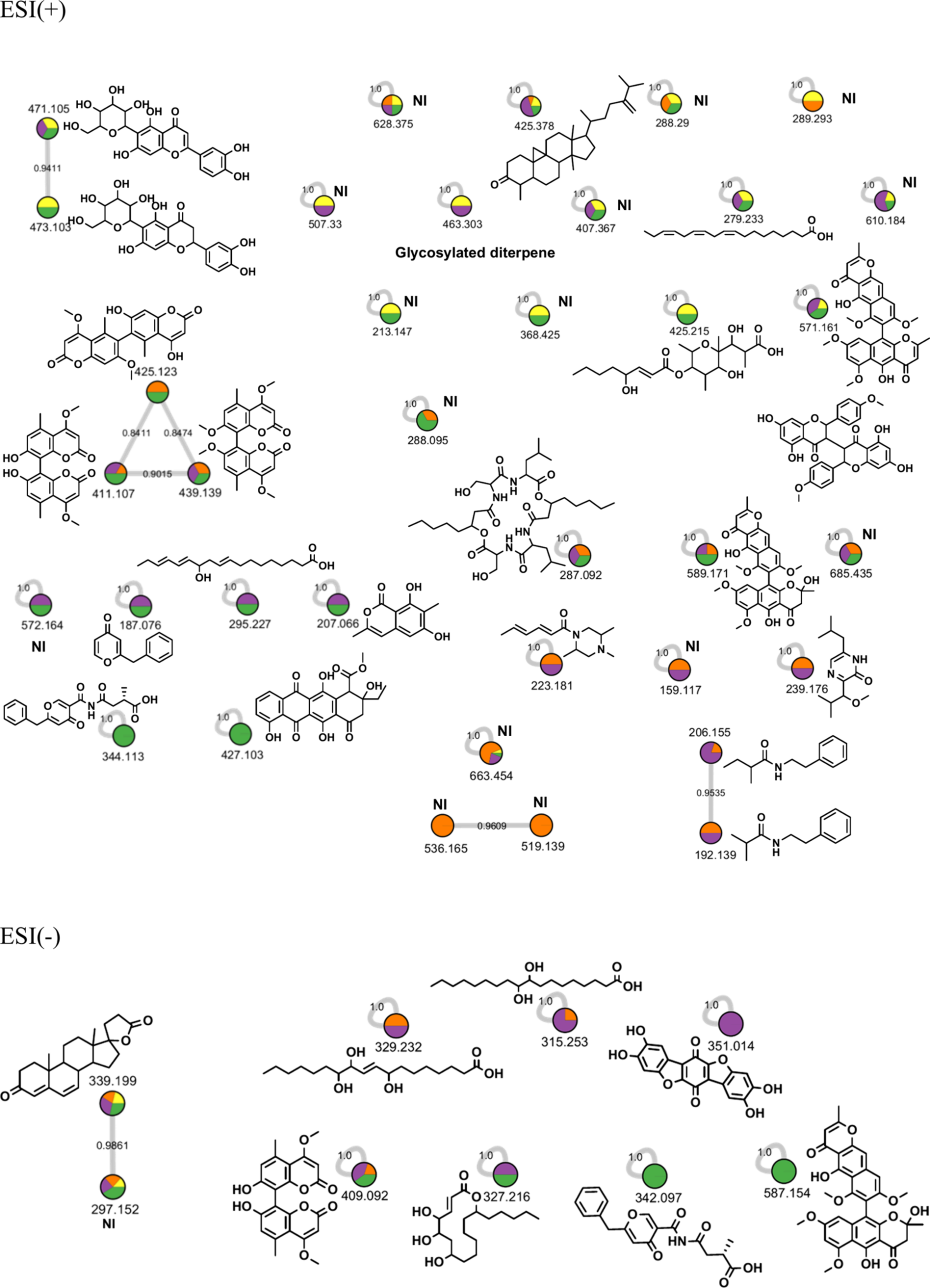
Molecular network from positive (ESI+) and negative (ESI−) ionization modes of extracts from *A. niger* grown on banana peel (BP), for 14, 21, and 28 days. Where yellow = BP, orange = 14 days, purple = 21 days, and green = 28 days.

Analysis of BPS_An14d, BPS_An21d, and BPS_An28d led to the detection of 45 nodes; among them, 20 were matched secondary metabolites in the GNPS database (Figure 6). Like the above-mentioned analysis, a cluster was formed among the two flavonoids, isoorientin and eriodictyol-6-C-β-d-glucopyranoside. A group was also formed by cylcoeucalenone, botcinic acid, and an unidentified compound. However, the combination might be due to the misinterpretation of the database, since one is a triterpene and the other is a polyketide. Part of the metabolites composed of flavasperone (*m*/*z* 287.0920), hexylcitric acid (*m*/*z* 275.1126), JBIR-138 (*m*/*z* 363.1438), β-d-glucopyranosyl aspergillusene A (*m*/*z* 441.2113), and the peptide related to Glu-Arg-Phe (*m*/*z* 449.2135) was found only in this analysis.

**Figure 6:**
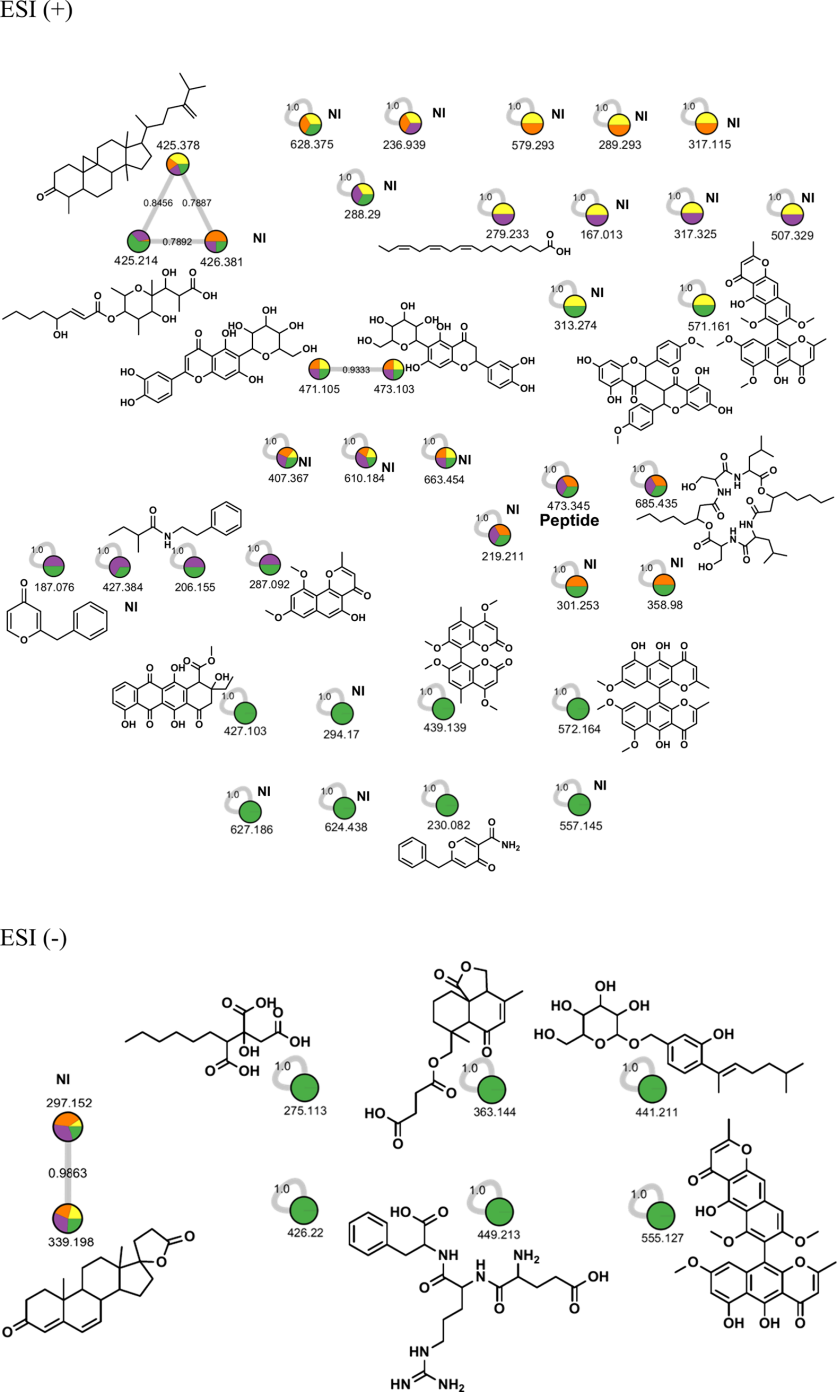
Molecular network from positive (ESI+) and negative (ESI−) ionization modes of extracts from *A. niger* grown on banana peel with starch (BPS), for 14, 21, and 28 days. Where in yellow = BP, orange = 14 days, purple = 21 days, and green = 28 days.

The studied fungus was also cultured on starch for 14, 21, and 28 days, affording Starch_An14d, Starch_An21d, and Starch_An28d, respectively (Table 3). LCMS analyses led to the detection of 41 compounds; among them, 29 were characterized. The fungus on starch medium produced fewer secondary metabolites than when grown on BP and BPS. Fifteen of these metabolites were already identified from other *Aspergillus* species, while eight were unique to *A. niger* and four to other species (Figure 7). The GNPS analyses depicted a node between the unidentified metabolites *m*/*z* 430.3890, 428.3730, 416.3730, and 402.3579, a sphingolipid identified as *N*-(propionyl)-4-hydroxyeicosasphinganine. The first metabolite (*m*/*z* = 430.3890) differs from the sphingolipid by 18 Da, indicating the presence of an OH group. Then, the metabolite *m*/*z* 428.3730 has a double-bond equivalence less than that of the first metabolite. The last metabolite (*m*/*z* 416.3730) is 14 Da higher than the sphingolipid, indicating a longer side chain by an additional CH_2_ group. GNPS obtained from ESI­(+) identified kotanin (*m*/*z* = 439.1380) and two related bicoumarins at *m*/*z* = 425.1230 (demethylkotanin) and 411.1380 (orlandin) in the same cluster. This analysis also depicted three steroids *m*/*z* 509.2750 (asperterpene I), 525.271, and 493.2800 forming a cluster the same as the sphingolipids at *m*/*z* 274.2750, 318.3010, and 362.3260. Aurasperone A (*m*/*z* 571.159) and fonsecinone B (*m*/*z* 589.171) differ by 18 Da, despite no node being observed between them.

**Table 3:** Identification of Metabolites Using MS-FINDER Software and UPLC-ESI-HRMS Analysis in Positive and Negative Modes for Extracts (Starch_An14d, Starch_An21d, and Starch_An28d) Obtained from the Fungus *A. niger* Grown on Starch at 14, 21, and 28 Days

no.	*t*_R_ (min)	Formula	Adduct type	Precursor*m*/*z*	Error (ppm)	Proposed identification	Score	Class	Time (day)	Specie	ref.
1	3.64	C_12_H_14_O_6_	[M-H]^−^	253.0716	0.64	4,6,8-trihydroxy-3-methoxy-3,7-dimethyl-4h-2-benzopyran-1-one	7.53	Hydroxybenzoic acid derivatives	14, 21, 28	*Aspergillus sp.*	^126^
2	4.62	C_9_H_16_O_4_	[M-H]^−^	187.0971	2.57	Aspinonene	5.82	Pentaketide	14, 21	*A. ochraceus*	^127^
3	5.05	C_11_H_10_O_4_	[M-H]^−^	205.0503	1.61	Similanpyrone B	7.72	Isocoumarins	14, 21, 28	*A. similanensis*	^33^
4	5.30	C_22_H_18_O_8_	[M-H]^−^	409.0921	1.93	Orlandin	6.26	Bicoumarin	14, 21, 28	*A. niger*	^36^
5	5.47	C_18_H_8_O_8_	[M-H]^−^	351.0138	2.39	Thelephoric acid	5.92	Terphenyl	14, 21, 28	mushroom *Polyozellus multiplex*	^38^
6	5.79	C_12_H_16_O_3_	[M-H]^−^	207.1024	1.29	(3R,4S)-3,4,5-Trimethylisochroman-6,8-diol	5.30	Chromanes	14, 21, 28	*A. sydowii*	^128^
7	5.88	C_31_H_50_O_8_	[M-H]^−^	549.3428	0.89	AB023	7.69	Macrolides	14, 21, 28	*Streptomyces sp.*	^129^
8	6.12	C_36_H_58_O_10_	[M+HCO_2_]^−^	695.3998	–	NI	–	–	14, 21, 28		–
9	6.32	C_35_H_54_O_9_	[M+HCO_2_]^−^	663.3741	–	NI	–	–	14, 21, 28	*–*	–
10	6.48	C_26_H_38_O_8_	[M+HCO_2_]^−^	523.2554	–	NI	–	–	14, 21, 28	*–*	–
11	6.52	C_18_H_34_O_5_	[M-H]^−^	329.2325	2.26	9,10,11-trihydroxyoctadec-12-enoic acid	8.21	Fatty acid	14, 21, 28	*Aspergillus sp.*	^98^
12	6.67	C_16_H_16_O_6_	[M-H]^−^	303.0862	3.98	Fonsecin B	6.73	Naphthopyranones	14, 21, 28	*A. niger*	^36^
13	6.71	C_14_H_20_O_3_	[M-H]^−^	235.1330	4.10	Terrefuranone	5.09	Furanones	14, 21, 28	*Aspergillus terreus*	^130^
14	6.72	C_12_H_20_O_4_	[M-H]^−^	227.1283	2.55	Hexylitaconic acid H	5.13	Fatty acid	14, 21, 28	*A. niger*	^92^
15	6.83	C_11_H_18_O_4_	[M-H]^−^	213.1129	1.55	Hexylitaconic acid	5.14	Fatty acid	14, 21, 28	*A. niger*	^92^
16	7.19	C_36_H_58_O_9_	[M+HCO_2_]^−^	679.4047	–	NI	–	–	14, 21, 28	*–*	–
17	7.50	C_27_H_40_O_9_	[M-H]^−^	507.2604	–0.87	Asperterpene I	<57.11	Steroids	14, 21, 28	*A. terreus*	^131^
			[M+HCO_2_]^−^	553.2660	–1.11						
18	7.74	C_30_H_48_O_5_	[M-HCO_2_]^−^	533.3472	–	NI		Steroid	14, 21, 28	***–***	–
19	7.84	C_32_H_30_O_12_	[M-H]^−^	605.1658	1.07	Aurasperone B	6.67	Naphtho-γ-pyrone	14, 21, 28	*A. niger*	^36^
20	8.22	C_14_H_31_NO	[M + H]^+^	230.2489	–4.62	Related to Xestoaminol c	–	Sphingolipid	14, 21, 28	*–*	–
21	8.31	C_18_H_34_O_4_	[M-H]^−^	313.2379	2.65	(Z)-12,13-dihydroxyoctadec-9-enoic acid	7.52	Fatty acid	14, 21, 28	*Aspergillus sp.*	^98^
22	8.33	C_25_H_49_NO_5_	[M + H]^+^	444.3679	1.02	Hydroxyoctadecanoylcarnitine	5.13	Acyl carnitines	21,28	*A. oryzae*	^132^
23	8.48	C_32_H_28_O_11_	[M + H]^+^	589.1703	0.24	Fonsecinone B or derivatives	5.94	Naphtho-γ-pyrone	14, 21, 28	*A. niger*	^36^
			[M-H]^−^	587.1562	–0.53						
24	8.82	C_26_H_38_O_6_	[M-HCO_2_]^−^	491.2652	2.40	NI		NI	14, 21	*–*	–
25	8.96	C_32_H_28_O_11_	[M + H]^+^	589.1707	–0.45	Fonsecinone B or derivatives	–	Naphtho-γ-pyrone	14, 21, 28	*A. niger*	^36^
26	9.09	C_32_H_26_O_10_	[M + H]^+^	571.1610	–1.80	Aurasperone A or derivatives	–	Naphtho-γ-pyrone	14, 21, 28	*A. niger*	^36^
27	9.10	C_18_H_28_O_3_	[M-H]^−^	291.1960	1.94	Related to 9-oxooctadeca-10,12,15-trienoic acid	5.53	Fatty acid	14, 21	*Aspergillus sp.*	^98^
28	9.14	C_18_H_32_O_4_	[M-H]^−^	311.2221	2.50	NI	–	Fatty acids	14, 21, 28	*–*	–
29	9.30	C_18_H_36_O_4_	[M-H]^−^	315.2530	3.11	9,10-dihydroxystearic acid	7.23	Fatty acid	14, 21, 28	*Aspergillus sp.*	^98^
30	9.34	C_33_H_58_O_14_	[M+HCO_2_]^−^	723.3806	0.36	NI		Monoglyceride disaccharide	14, 21, 28	*–*	–
31	9.43	C_18_H_30_O_3_	[M-H]^−^	293.2112	3.46	Psiaα	–	Fatty alcohol	14, 21, 28	*A. nidulans*	^37^
32	9.54	C_46_H_34_O_6_	[M-H]^−^	681.2310	–4.01	NI	–	NI	14, 21, 28	*–*	–
33	9.60	C_27_H_46_O_9_	[M+HCOO]^−^	559.3123	0.17	NI	–	Monoglyceride disaccharide	28	*–*	–
34	9.77	C_18_H_32_O_3_	[M-H]^−^	295.2273	1.92	FA 18:2 + 1O	–	Fatty acid	21, 28	*Aspergillus sp.*	^98^
35	9.78	C_25_H_47_NO_4_	[M + H]^+^	426.3576	0.44	Oleoylcarnitine	5.53	Acyl carnitines	28	*A. oryzae*	^132^
36	10.20	C_23_H_47_NO_4_	[M + H]^+^	402.3579	–0.29	Related to *N*-(propionyl)-4-hydroxyeicosasphinganine	–	Ceramide	21, 28	*–*	–
37	10.48	C_18_H_34_O_3_	[M-H]^−^	297.2431	1.40	FA 18:1 + 1O	6.55	Fatty acid	28	*Aspergillus sp.*	^98^
38	10.48	C_27_H_53_NO_8_	[M-H]^−^	518.3697	0.27	Related to *N-*(propionyl)-1-O-beta-d-glucosyl-sphinganine	–	Cerebroside	28	*–*	–
39	10.48	C_27_H_48_O_9_	[M+HCO_2_]^−^	561.3282	–0.29	NI	–	Monoglyceride glycoside	28	*–*	–
40	11.03	C_18_H_32_O_3_	[M + H]^+^	297.2426	–0.60	FA 18:2 + 1O	6.80	Fatty acid	14, 21, 28	*Aspergillus sp.*	^98^
			[M-H]^−^	295.2275	1.24						
41	12.55	C_35_H_62_O_8_	[M-H]^−^	655.4420	1.02	NI	<5	–	28	*–*	–

**Figure 7:**
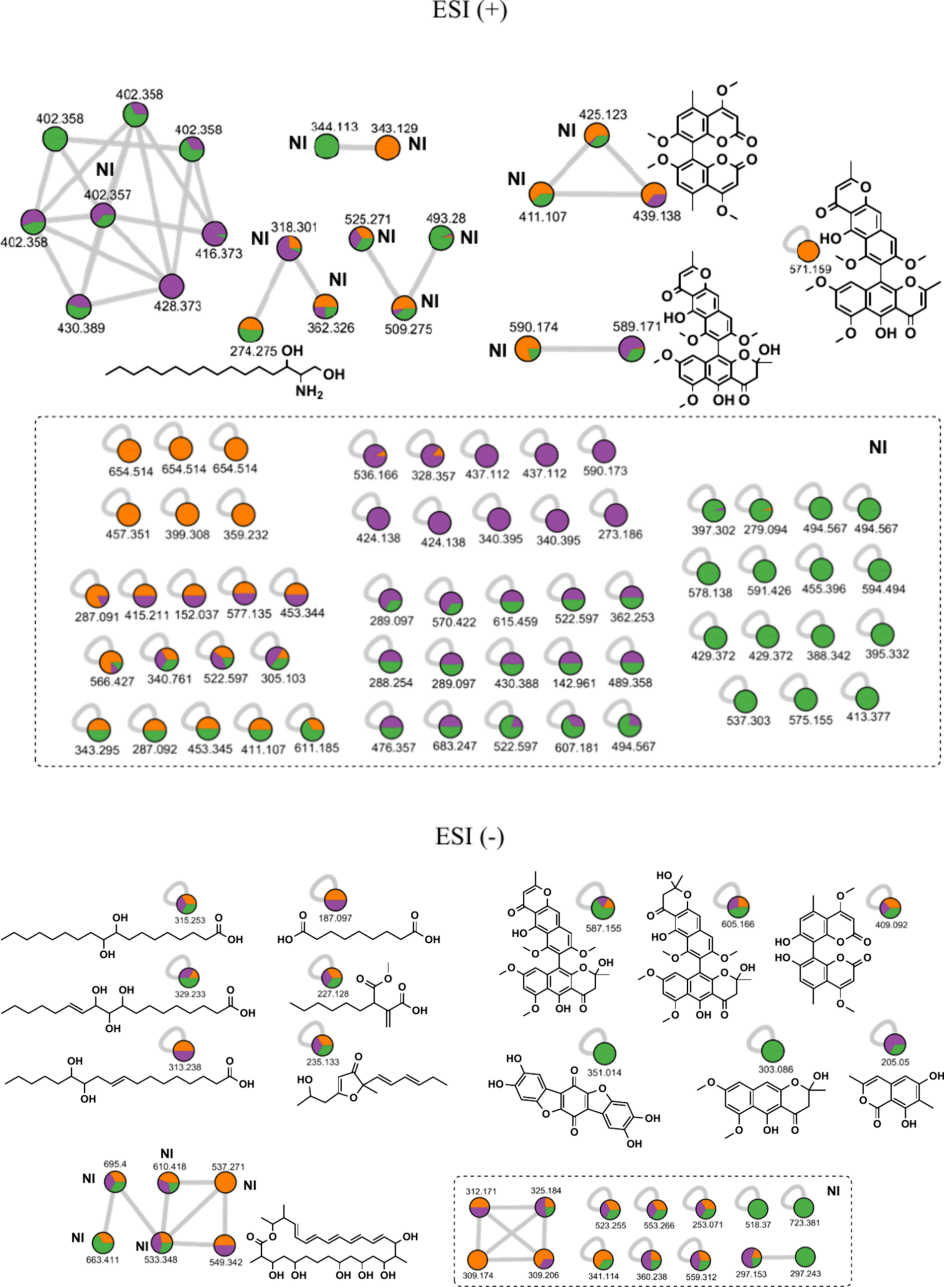
Molecular network extracts in positive (ESI+) and negative (ESI−) ionization modes of *A. niger* grown on starch for 14, 21, and 28 days, where orange = 14 days, purple = 21 days, and green = 28 days.

GNPS obtained from ESI(−) also revealed the presence of polyhydroxylated fatty acids, a fatty diacid, and two aliphatic polyketides. In addition to two naphthopyrones, a naphthopyranone, a bicoumarin, an isocoumarin, and a terphenyl derivative were also characterized. A macrolide with a structure related to the five others was found.

The growth of a microorganism depends on the nutritional value of the culture medium, alongside other factors, such as pH, temperature, time of cultivation, and solid/liquid conditions. Therefore, the modification of one of these parameters can affect or promote growth with variation in metabolite profiles.^65^

Banana peels and starch media yielded seven identical compounds, namely, similanpyrone B (*m*/*z* 205.0503), orlandin (*m*/*z* 409.0921), thelephoric acid (*m*/*z* 351.0138), hexylitaconic acid (*m*/*z* 213.1129), fonsecinone B (*m*/*z* 589.1703), aurasperone A (*m*/*z* 571.1610), and Psiaα (*m*/*z* 293.2112). Only three compounds (similanpyrone B, orlandin, and hexylitaconic acid) were simultaneously found in starch-enriched banana peel and starch media.

Comparison of secondary metabolite profiles from both culture media revealed that BP_An medium produces more chemomarkers (27 compounds) from *A. niger* than the starch-enriched medium (22 compounds) (Figures 8 and 9). This latter promoted significantly the production of chemo-markers (19 compounds) already reported in the *Aspergillus* genus than BP_An medium (14 compounds). The increase in the carbohydrate amount was beneficial to produce more secondary metabolites (87 compounds in BPS_An against 66 in BP). The BPS_An medium generated 26 nonidentified (NI) compounds, while BP_An produced half of this number.

**Figure 8:**
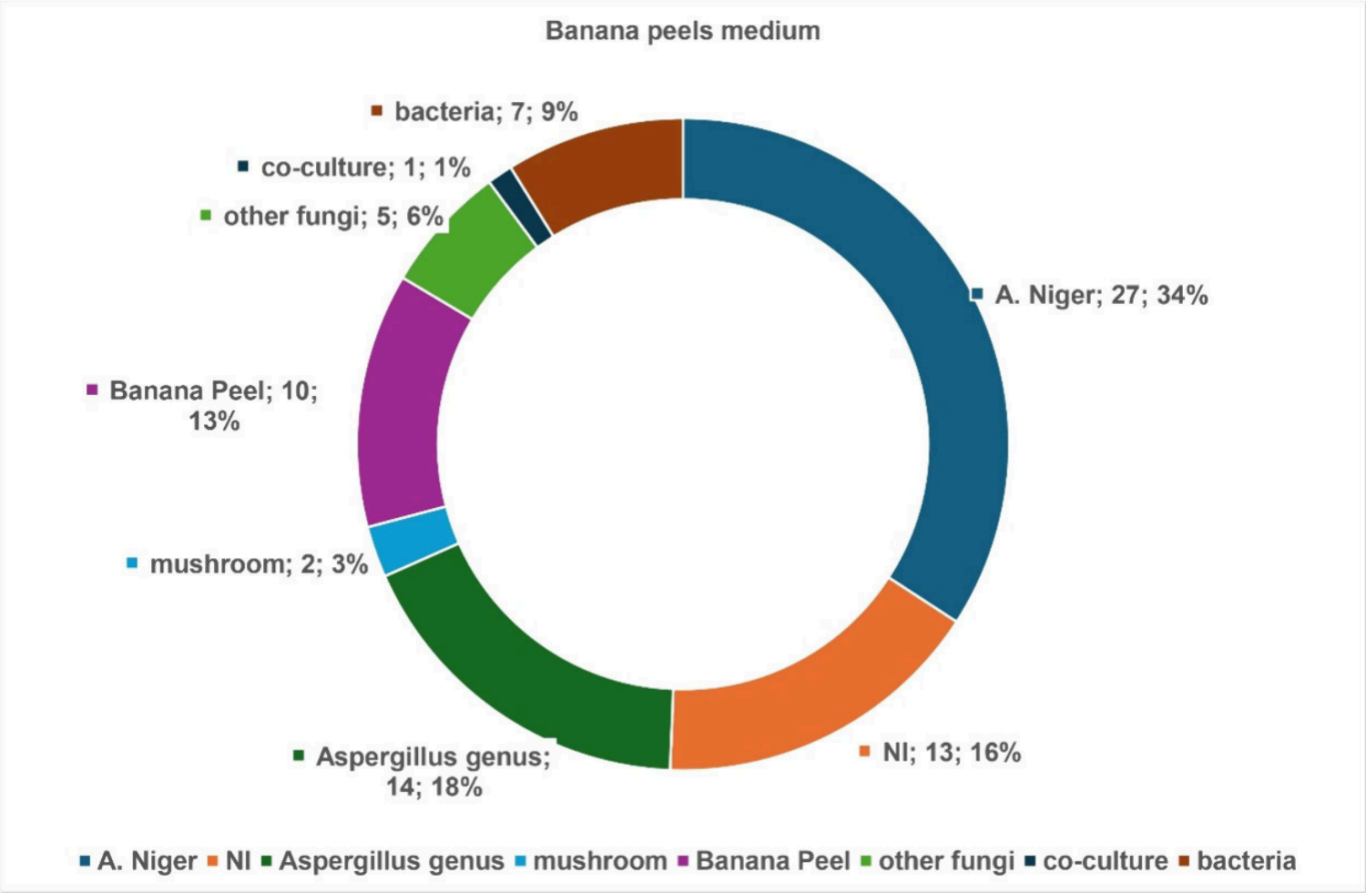
Illustration of the origin distribution across various species for the secondary metabolites produced in BP media (BP_An14d, BP_An21d, and BP_An28d).

**Figure 9:**
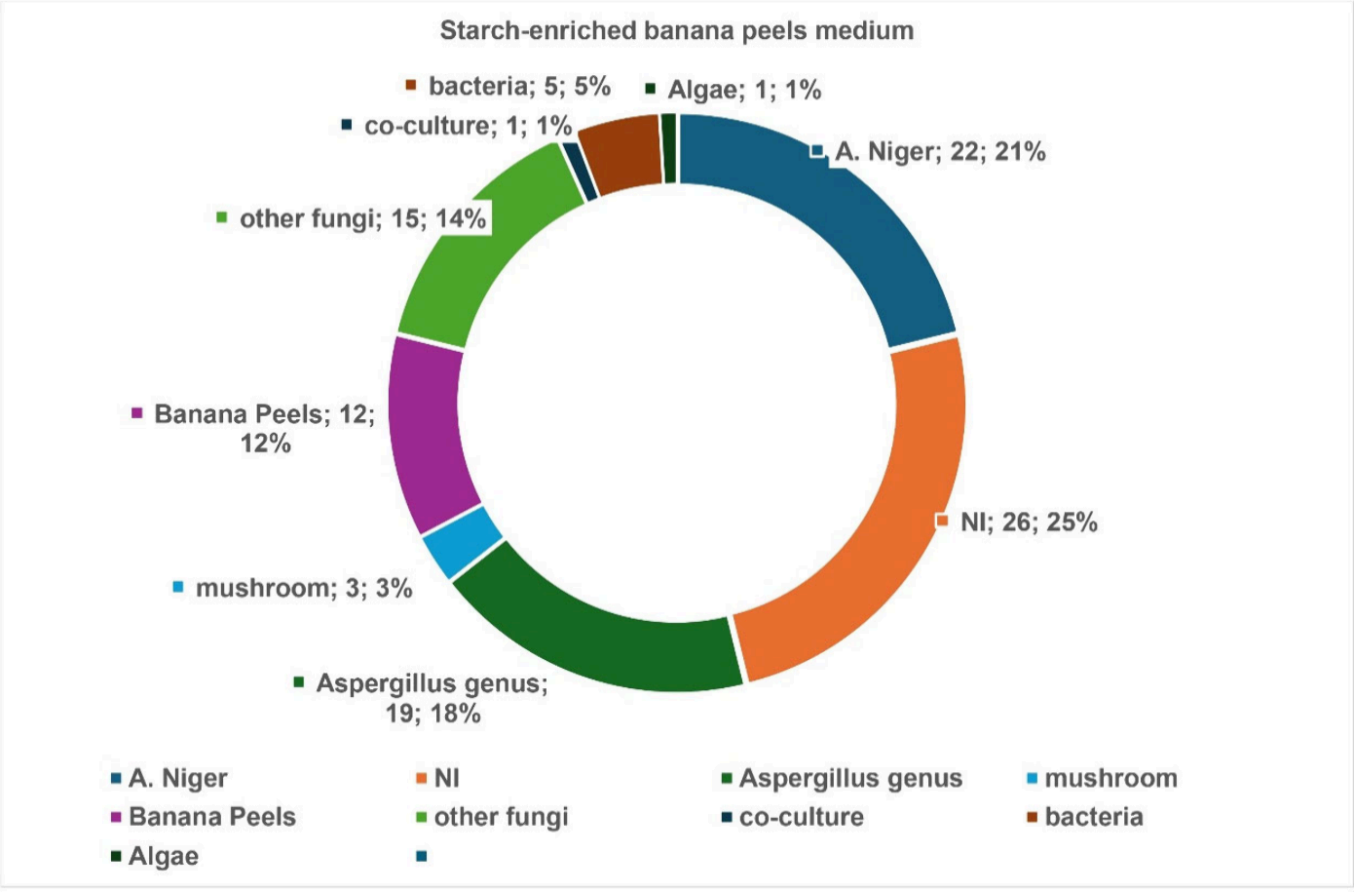
Distribution of the secondary metabolites produced in BPS media (BPS_An14d, BPS_An21d, and BPS_An28d) among various species.

Part of the NI metabolites did not produce sufficient fragment ions, enabling their characterization, and others could not be matched either with a known compound or a class of compounds. NI compounds might be produced after the activation of silent gene expression. Also, during the study, four triterpenes were detected, among them two with *m*/*z* 457.3679 (14, 21, and 28 days) and two with *m*/*z* 425.3776 (14 and 28 days). These compounds could be biotransformation products of the triterpene cycloeucalenone found in banana peels.

As shown in Tables 1–3, some of the compounds from the cultures are produced by *A. niger* for the first time, although already identified in other microorganisms. The variation in these chemical profiles suggests that the studied *A. niger* may have undergone gene alteration. Fungi generate an array of natural compounds, and these products can change if the fungi undergo mutations caused by stress. An earlier study showed that sodium azide induced mutagenic changes in *A. niger*, which were monitored using RAPD-PCR analysis.^66^ One of the strategies to mutate these genes and promote one-strain-many-compounds (OSMAC) is to systematically change the culture conditions.^67,68^ It is also noteworthy to point out that banana peel secondary metabolites could interfere with gene expression or deletion. Isoorientin, a flavonoid from banana peels, possesses antimicrobial activity and is known to cause mitochondrial dysfunction in cancer cells.^69,70^ Thus, its presence in the culture medium could compel the fungus to develop adaptive mechanisms that include gene expression. The second flavonoid, eriodictyol-6-*C*-β-d-glucopyranoside, was not studied for its antifungal activity. However, previous work suggested that flavonoids are part of the plant metabolites produced in response to fungal infection.^71^ In addition, banana peel extract from an incompletely identified *Musa* species showed antifungal activity by inhibiting 72% of *A. niger* growth.^72^

The present work used a food residue (banana peels) as a biomass to culture *A. niger*. During the investigation, some metabolites were produced, as depicted in Tables 1–3. Part of these metabolites showed pharmacological properties in previous studies. Aurasperone displayed IC_50_ of 12.25 μM and antiviral activity against SARS-CoV-2.^73^ It also presents a high selectivity index of 2641.5. This compound also proved to be an antioxidant and was shown to possess a protective effect on CHO cell lines against H_2_O_2_.^74^ Hexylitaconic acid inhibited with IC_50_ of 50 μg/mL the human double minute 2 (HDM2), a protein overexpressed in many human cancers that interferes with the tumor-suppressing functionality of p53.^75^ This compound also promoted up to 13 and 66.3% of the germination and biomass in cauliflower seedlings.^76^ Pyranonigrin A showed anti-inflammatory activity by reducing the level of NO production.^77,78^

Banana peel is rich in nutrients, including minerals, carbohydrates (xylose, pectin, cellulose), amino acids, and fatty acids^7,8^ that could favor the growth of *A. niger*. However, it also contains secondary metabolites that possess antimicrobial activity, causing a strong stress to the fungus growth.^7,8^ The chemical profile of the peel’s crude extract showed the presence of secondary metabolites, namely, isoorientin, eriodictyol 6-C-β-d-glucopyranoside, hexadecasphinganine, 1,10-epoxy-8α-methoxyermophilanolide, and di­(2-ethylhexyl)­phthalate (Tables 1 and 2). Isoorientin was found in an ethanolic extract of *Mentha arvensis* that displayed strong antibacterial activity against bacteria from the ESKAPE consortium.^79^ In addition, this metabolite showed strong antimicrobial and antibiofilm activity against *E. coli* by interacting with the extracellular polymeric substances.^69^ Flavonoids with an antibiofilm property also affected the *A. flavus* cell wall by causing osmotic stress as shown in a previous report. In response to this stress, the fungus increased its production of ergosterol.^80^ In addition, *A. flavus* exposure to d-glucal led to the suppression of aflatoxin biosynthesis and greatly increased kojic acid production.^81^ The stress induced by banana peels’ secondary metabolites might explain the presence of wall cell metabolites such as cholesterol, polyhydroxylated fatty acid derivatives, and compounds not previously found in the studied fungus.

The starch-enriched banana peel medium yielded more metabolites, with 28 days of fermentation proving to be the optimal condition. Sabat and co-workers similarly found antifungal profiles against *Verticillium wilt* of *Penicillium steckii* extracts produced on different carbohydrate-enriched media.^82^ Vandermolen and co-workers also demonstrated that three fungal strains presented different secondary metabolite profiles after growing them separately on rice, grits, oatmeal, and wheat germ flour.^65^ Other previous studies revealed that to overcome stress caused by pH, nutrients, temperature, osmosis, and oxidative agents, fungi can increase, suppress, and promote the synthesis of some secondary metabolites.^83,84^

Overall, the presence of banana peels in BP and BPS media seemed to increase the production of fungal secondary metabolites. Banana peels, secondary metabolites, seem to play a key role in the expression of genes that regulate several biosynthesis pathways.

## 4 Conclusion

Suitable management of food residue and waste can bring numerous beneficial outcomes, such as renewable energy and valuable biomolecules with applications in drug discovery, veterinary medicine, and agriculture. Thus, the present study aimed to unveil biomolecules produced by *A. niger* when grown on banana peels (BP) used as biomass. The study also enriched BP (BPS) with starch to understand the chemical changes that can occur when the fungus grows in this medium. The combination of in silico statistical tools (metaboanalyst) and databases for dereplication (GNPS, MS-FINDER, and MS-DIAL) enabled us to establish and compare the chemical profile according to cultivation periods (14, 21, and 28 days) and culture medium. This unprecedented study reveals that the chemical profiles of *A. niger* grown on BP and BPS were composed of 76 and 99 substances, respectively. Alongside banana peel metabolites, polyketides, cyclopeptides, citric acid derivatives, alkamides, and sesquiterpenes were characterized, among others. Statistical analysis revealed that banana peel medium can produce secondary metabolites with pharmacological potential independent of the fermentation period. However, the starch-enriched banana peel medium optimally produced fungal compounds during the 28-day fermentation.

The present study provides a cheap process to induce and modulate fungal valuable molecule production and a method to turn waste into valuable products.

## Supplementary Material



## Data Availability

The data supporting the findings of this study are available from the corresponding author upon reasonable request.

## Notes

TThe Supporting Information is available free of charge at https://pubs.acs.org/doi/10.1021/acsomega.5c10614.

Characterization and identification of the fungus *Aspergillus niger*, chromatograms obtained from MS^E^ UPLC-ESI-QTOF-MS analysis, Tables S1 and S2, and Schemes S1–S6 (PDF)

## Abbreviations

GNPS: Global Natural Products Social Molecular Networking
PC: Principal component
UN: United Nations
UPLC-ESI-MS: Ultra-Performance Liquid Chromatography coupled to Electrospray Mass Spectrometry
MS/MS: Tandem mass
ITS: Internal transcribed spacer
B.O.D: Biological Oxygen Demand

## References

[1] United Nations Environment Programme (2024) An inside look Brazil’s push to end food waste. https://www.unep.org/news-and-stories/story/inside-look-brazils-push-end-food-waste. Accessed in April 2025.

[2] United Nations Environment Programme (2024) Food Waste Index Report 2024 . Think Eat Save: Tracking Progress to Halve Global Food Waste. Food Waste Index Report 2024 | UNEP - UN Environment Programme . Accessed in April 2025.

[3] Instituto Brasileiro de Geografia e Estatística (IBGE), Produção de banana 2024 . Online access: https://www.ibge.gov.br/explica/producao-agropecuaria/banana/br. Accessed in November 2025.

[4] Ribeiro G. R., Steinmetz R. L. R., Wisbeck E. (2025). Biogas production from solid residues
generated in bioethanol production using banana biomass. Rev. Bras. Eng. Agríc. Ambient..

[5] Schieber A. (2017). Side Streams
of Plant Food Processing As a Source of Valuable Compounds: Selected
Examples. Annu. Rev. Food Sci. Technol..

[6] Parra-Pacheco B., Cruz-Moreno B. A., Aguirre-Becerra H., García-Trejo J.
F., Feregrino-Pérez A. A. (2024). Bioactive
Compounds from Organic
Waste. Molecules.

[7] Hikal W. M., Said-Al Ahl H. A. H., Bratovcic A., Tkachenko K. G., Sharifi-Rad J., Kačániová M., Elhourri M., Atanassova M. (2022). Banana Peels:
A Waste Treasure for
Human Being. Evidence-based complementary and
alternative medicine: eCAM.

[8] Wani K. M., Dhanya M. (2025). Unlocking the potential of banana peel bioactives:
extraction methods, benefits, and industrial applications. Discover Food.

[9] Benjamin S., Pandey A. (1997). Coconut cake – a potent substrate
for the production
of lipase by Candida rugosa in solid-state fermentation. Acta Biotechnol.

[10] Melini F., Melini V. (2024). Role of Microbial Fermentation in the Bio-Production
of Food Aroma Compounds from Vegetable Waste. Fermentation.

[11] Panda S. K., Mishra S. S., Kayitesi E., Ray R. C. (2016). Microbial-processing
of fruit and vegetable wastes for production of vital enzymes and
organic acids: Biotechnology and scopes. Environ.
Res..

[12] Pinela J., Añibarro-Ortega M., Barros L. (2024). Food Waste Biotransformation
into Food Ingredients: A Brief Overview of Challenges and Opportunities. Foods.

[13] Sabu A., Sarita S., Pandey A., Bogar B., Szakacs G., Soccol C. R. (2002). Solid-state fermentation for production of phytase
by Rhizopus oligosporus. Appl. Biochem. Biotechnol..

[14] de
Castro R. J. S., Ohara A., Nishide T. G., Bagagli M. P., Dias F. F. G., Sato H. H. (2015). A versatile system based on substrate
formulation using agroindustrial wastes for protease production by
Aspergillus niger under solid state fermentation. Biocatal Agric. Biotechnol..

[15] El-Naggar M. Y., El-Assar S. A., Abdul-Gawad S. M. (2009). Solid-state
fermentation for the
production of meroparamycin by *Streptomyces* sp. strain
MAR01. J. Microbiol Biotechnol.

[16] Vastrad B., Neelagund S. (2011). Optimization
and production of neomycin from different
agro industrial wastes in solid state fermentation. Int. J. Pharm. Sci. Drug Res..

[17] Sadh P. K., Kumar S., Chawla P., Duhan J. S. (2018). Fermentation: A
boon for production of bioactive compounds by processing of food industries
wastes (By-Products). Molecules.

[18] Laothanachareon T., Bunterngsook B., Champreda V. (2022). Profiling multi-enzyme activities
of *Aspergillus niger* strains growing on various agro-industrial
residues. 3 Biotech.

[19] Khairan K., Makstum A., Yulvizar C. (2019). Utilization
of banana peel waste
for citric acid production by *Aspergillus niger*. IOP Conf Ser. Earth Environ. Sci..

[20] Barman S., Sit N., Badwaik L. S., Deka S. C. (2014). Pectinase production by *Aspergillus
niger* using banana (*Musa balbisiana*) peel
as substrate and its effect on clarification of banana juice. J. Food Sci. Technol..

[21] Riddell R. W. (1950). Permanent
Stained Mycological Preparations Obtained by Slide Culture. Mycologia.

[22] Robl D., da Silva Delabona P., dos Santos Costa P., da Silva Lima D. J., Rabelo S. C., Pimentel I. C. (2015). Xylanase production
by endophytic *Aspergillus niger* using pentose-rich
hydrothermal liquor from sugarcane bagasse. Biocatal. Biotransform..

[23] Cavalcante S. B., da Silva A. F., Pradi L. (2024). Antarctic fungi produce
pigment with antimicrobial and antiparasitic activities. Braz. J. Microbiol..

[24] Ceccaroli P., Saltarelli R., Cesari P. (2001). Effects
of different
carboyhydrate sources on the growth of Tuber *borchii Vittad*. mycelium strains in pure culture. Mol. Cell.
Biochem..

[25] Maseko K. H., Regnier T., Wokadala O. C., Bartels P., Meiring B. (2024). Effect of
Culture Media on the Yield and Protein Content of *Pleurotus
ostreatus* (Jacq.) Kumm Mycelia. Int.
J. Food Sci..

[26] Ubalua A. (2014). Sweet Potato
Starch as a Carbon Source for Growth and Glucoamylase Production from *Aspergillus niger*. Advances in Microbiology.

[27] Tsugawa H., Cajka T., Kind T., Ma Y., Higgins B., Ikeda K., Kanazawa M., VanderGheynst J., Fiehn O., Arita M. (2015). MS-DIAL: data-independent MS/MS deconvolution
for comprehensive metabolome analysis. Nat.
Methods.

[28] Wang X., Li N., Chen S., Ge Y. H., Xiao Y., Zhao M., Wu J. L. (2022). MS-FINDER Assisted in Understanding the Profile of Flavonoids in
Temporal Dimension during the Fermentation of Pu-erh Tea. J. Agric. Food. Chem..

[29] Wang M., Carver J. J., Phelan V. V. (2016). Sharing and community
curation of mass spectrometry data with Global Natural Products Social
Molecular Networking. Nat. Biotechnol..

[30] Hiort J., Maksimenka K., Reichert M., Perović-Ottstadt S., Lin W. H., Wray V., Steube K., Schaumann K., Weber H., Proksch P., Ebel R., Müller W. E., Bringmann G. (2004). Ne1w natural products from the sponge-derived fungus *Aspergillus niger*. J. Nat. Prod..

[31] Yu R., Liu J., Wang Y., Wang H., Zhang H. (2021). *Aspergillus
niger* as a Secondary Metabolite Factory. Front. Chem..

[32] Awad G., Mathieu F., Coppel Y., Lebrihi A. (2005). Characterization and
regulation of new secondary metabolites from *Aspergillus ochraceus* M18 obtained by UV mutagenesis. Can. J. Microbiol..

[33] Prompanya C., Dethoup T., Bessa L. J., Pinto M. M., Gales L., Costa P. M., Silva A. M., Kijjoa A. (2014). New isocoumarin derivatives
and meroterpenoids from the marine sponge-associated fungus *Aspergillus similanensis* sp. nov. KUFA 0013. Mar Drugs.

[34] Dickschat J. S., Reichenbach H., Wagner-Döbler I., Schulz S. (2005). Novel Pyrazines
from the Myxobacterium Chondromyces crocatus and Marine Bacteria. Eur. J. Org. Chem..

[35] Akiyama K., Teraguchi S., Hamasaki Y., Mori M., Tatsumi K., Ohnishi K., Hayashi H. (2003). New dimeric naphthopyrones from *Aspergillus niger*. J. Nat. Prod..

[36] Lima M. A.
S., De Oliveira M. D. C. F., Pimenta A. T. Á., Uchôa P. K. S. (2019). *Aspergillus niger*: A hundred years
of contribution to the natural products chemistry. J. Braz. Chem. Soc..

[37] Mazur P., Meyers H., Nakanishi K. A. A., Champe S. P. (1990). Structural elucidation
of sporogenic fatty acid metabolites from *Aspergillus nidulans*. Tetrahedron Lett..

[38] Nagasawa I., Kaneko A., Suzuki T., Nishio K., Kinoshita K., Shiro M., Koyama K. (2014). Potential
anti-angiogenesis effects
of p-terphenyl compounds from Polyozellus multiplex. J. Nat. Prod..

[39] Steffan B., Steglich W. (1984). Die Hutfarbstoffe des
Maronenröhrlings (*Xerocomus badius*). Angew. Chem., Int.
Ed..

[40] Li B., Huang Q. X., Gao D., Liu D., Ji Y. B., Liu H. G., Lin W. H. (2015). New C13
lipids from the marine-derived
fungus *Trichoderma harzianum*. J. Asian Nat. Prod. Res..

[41] Pinto A. A., Barúa J. E., Almeida M. O., Viaud M., Zorrilla D., Collado I. G., Macías-Sánchez A. J., Durán-Patrón R. (2022). Structural
and biosynthetic studies
of botrycinereic acid, a new cryptic metabolite from the fungus *Botrytis cinerea*. Bioorg. Chem..

[42] Oh D. C., Gontang E. A., Kauffman C. A., Jensen P. R., Fenical W. (2008). Salinipyrones
and pacificanones, mixed-precursor polyketides from the marine actinomycete *Salinispora pacifica*. J. Nat. Prod..

[43] Tishler M., Sampson W. L. (1948). Isolation of vitamin
K2 from cultures of a spore-forming
soil bacillus. Proc. Soc. Exp. Biol. Med..

[44] Demarque D. P., Crotti A. E., Vessecchi R., Lopes J. L., Lopes N. P. (2016). Fragmentation
reactions using electrospray ionization mass spectrometry: an important
tool for the structural elucidation and characterization of synthetic
and natural products. Nat. Prod. Rep..

[45] Geris R., Teles de Jesus V. E., Ferreira da Silva A., Malta M. (2024). Exploring Culture Media
Diversity to Produce Fungal Secondary Metabolites and Cyborg Cells. Chem. Biodivers..

[46] Antia B. S., Aree T., Kasettrathat C., Wiyakrutta S., Ekpa O. D., Ekpe U. J., Mahidol C., Ruchirawat S., Kittakoop P. (2011). Itaconic acid derivatives and diketopiperazine
from
the marine-derived fungus *Aspergillus aculeatus* CRI322–03. Phytochemistry.

[47] Fayek M., Ebrahim H. Y., Elsayed H. E., Abdel-Aziz M. S., Kariuki B. M., Moharram F. A. (2022). Anti-prostate cancer
metabolites
from the soil-derived *Aspergillus neoniveus*. Front. Pharmacol..

[48] Li G. Y., Yang T., Luo Y. G., Chen X. Z., Fang D. M., Zhang G. L. (2009). Brevianamide J, a new indole alkaloid
dimer from fungus
Aspergillus versicolor. Org. Lett..

[49] Wang C. C., Chiang Y. M., Praseuth M. B., Kuo P. L., Liang H. L., Hsu Y. L. (2010). Asperfuranone from *Aspergillus nidulans* inhibits proliferation of human non-small
cell lung cancer A549
cells via blocking cell cycle progression and inducing apoptosis. Basic Clin. Physiol. Pharmacol..

[50] Wang J., Yao Q. F., Amin M., Nong X. H., Zhang X. Y., Qi S. H. (2017). Penicillenols from
a deep-sea fungus *Aspergillus restrictus* inhibit
Candida albicans biofilm formation and hyphal growth. J. Antibiot..

[51] Liu Z., Zhao J. Y., Sun S. F., Li Y., Qu J., Liu H. T., Liu Y. B. (2019). Sesquiterpenes from an Endophytic. Aspergillus flavus. J. Nat. Prod.

[52] Ahluwalia V., Kumar J., Rana V. S., Sati O. P., Walia S. (2015). Comparative
evaluation of two *Trichoderma harzianum* strains for
major secondary metabolite production and antifungal activity. Nat. Prod. Res..

[53] Ma B. J., Hu Q., Liu J. K. (2006). A new p
-terphenyl derivative from fruiting bodies
of the basidiomycete *Sarcodon laevigatum*. J. Basic Microbiol..

[54] Ojima K. I., Yangchum A., Laksanacharoen P., Tasanathai K., Thanakitpipattana D., Tokuyama H., Isaka M. (2018). Cordybislactone, a
stereoisomer of the 14-membered bislactone clonostachydiol, from the
hopper pathogenic fungus Cordyceps sp. BCC 49294: revision of the
absolute configuration of clonostachydiol. J.
Antibiot..

[55] Isaka M., Chinthanom P., Kongthong S., Supothina S., Ittiworapong P. (2010). Hamigeromycins C–G, 14-membered
macrolides from
the fungus *Hamigera avellanea* BCC 17816. Tetrahedron.

[56] Yamada T., Iritani M., Doi M., Minoura K., Ito T., Numata A. (2001). Absolute stereostructures of cell-adhesion inhibitors,
macrosphelides C, E–G and I, produced by a Periconia species
separated from an Aplysia sea hare. J. chem.
Soc. Trans..

[57] Davoli P., Weber R. W. S. (2002). Simple method
for reversed-phase high-performance liquid
chromatographic analysis of fungal pigments in fruit-bodies of Boletales
(Fungi*)*. J. Chromatogr A.

[58] Hussain H., Ahmed I., Schulz B., Draeger S., Krohn K. (2012). Pyrenocines
J-M: four new pyrenocines from the endophytic fungus. Phomopsis sp. Fitoterapia.

[59] Chinworrungsee M., Kittakoop P., Isaka M., Rungrod A., Tanticharoen M., Thebtaranonth Y. (2001). Antimalarial halorosellinic acid from the marine fungus
Halorosellinia oceanica. Bioorg. Med. Chem.
Lett..

[60] Yuan C., Wang H., Wu C., Jiao Y., Li M. R., Wang Y., Wang S., Zhao Z., Lou H. (2013). Austdiol,
fulvic acid and citromycetin derivatives from an endolichenic fungus. Myxotrichum sp. Phytochem. Lett..

[61] Hsiao Y., Cheng M. J., Chang H. S., Wu M. D., Hsieh S. Y., Liu T. W., Lin C. H., Yuan G. F., Chen I. S. (2016). Six new
metabolites produced by *Colletotrichum aotearoa* 09F0161,
an endophytic fungus isolated from Bredia oldhamii. Nat. Prod. Res..

[62] Narasimhachari N., Vining L. C. (1972). Herqueichrysin,
a new phenalenone antibiotic from *Penicillium herquei*. J. Antibiot..

[63] Zang Y., Genta-Jouve G., Retailleau P., Escargueil A., Mann S., Nay B., Prado S. (2016). Talaroketals A and
B, unusual bis­(oxaphenalenone) spiro and fused ketals from the soil
fungus Talaromyces stipitatus ATCC 10500. Org.
Biomol. Chem..

[64] Thines E., Anke H., Sterner O. (1998). Trichoflectin, a bioactive azaphilone
from the ascomycete *Trichopezizella nidulus*. J. Nat. Prod..

[65] Vandermolen K. M., Raja H. A., El-Elimat T., Oberlies N. H. (2013). Evaluation of culture
media for the production of secondary metabolites in a natural products
screening program. AMB Express.

[66] Tawfik E., Alqurashi M., Aloufi S., Alyamani A., Baz L., Fayad E. (2022). Characterization
of Mutant *Aspergillus niger* and
the Impact on Certain Plants. Sustainability.

[67] Xu Q., Zhu C. Y., Wang M. S., Sun X. P., Li H. Y. (2014). Improvement
of a gene targeting system for genetic manipulation in *Penicillium
digitatum*. J. Zhejiang Univ. Sci. B.

[68] Cheng J. T., Yu J. H., Sun C. F., Cao F., Ying Y. M., Zhan Z. J., Li W. J., Chen X. A., Zhao Q. W., Li Y. Q., Gan L. S., Mao X. M. (2021). A Cell Factory of
a Fungicolous Fungus *Calcarisporium arbuscula* for
Efficient Production of Natural Products. ACS
Synth. Biol..

[69] Zuo J., Liu L., Hou S., Liu X., Teng J., Li P., Liu X. (2023). Antimicrobial and antibiofilm activity of isoorientin
against carbapenem
non-sensitive *Escherichia coli* from raw milk of goats. J. Anim. Sci..

[70] Yuan L., Wang J., Xiao H., Xiao C., Wang Y., Liu X. (2012). Isoorientin induces apoptosis through mitochondrial dysfunction and
inhibition of PI3K/Akt signaling pathway in HepG2 cancer cells. Toxicol. Appl. Pharmacol..

[71] Wang L., Chen M., Lam P. Y., Dini-Andreote F., Dai L., Wei Z. (2022). Multifaceted roles
of flavonoids mediating plant-microbe
interactions. Microbiome.

[72] Hashim M., Akbar A., Gul Z., Bilal Sadiq M., Khan Achakzai J., Ahmad Khan N. (2024). Fermentation
impact: A comparative
study on the functional and biological properties of Banana peel waste. Heliyon.

[73] ElNaggar M. H., Abdelwahab G. M., Kutkat O., GabAllah M., Ali M. A., El-Metwally M. E. A., Sayed A. M., Abdelmohsen U. R., Khalil A. T. (2022). Aurasperone A Inhibits SARS CoV-2 In Vitro: An Integrated
In Vitro and In Silico Study. Mar. Drugs.

[74] Carboué Q., Maresca M., Herbette G., Roussos S., Hamrouni R., Bombarda I. (2020). Naphtho-Gamma-Pyrones
Produced by *Aspergillus
tubingensis* G131: New Source of Natural Nontoxic Antioxidants. Biomolecules.

[75] Tsukamoto S., Yoshida T., Hosono H., Ohta T., Yokosawa H. (2006). Hexylitaconic
acid: a new inhibitor of p53-HDM2 interaction isolated from a marine-derived
fungus. Arthrinium sp. Bioorg. Med. Chem. Lett..

[76] Mondal G., Dureja P., Sen B. (2000). Fungal metabolites from Aspergillus
niger AN27 related to plant growth promotion. Indian J. Exp. Biol..

[77] Quang T. H., Phong N. V., Anh L. N., Hanh T. T. H., Cuong N. X., Ngan N. T. T., Trung N. Q., Nam N. H., Minh C. V. (2022). Secondary
metabolites from a peanut-associated fungus *Aspergillus niger* IMBC-NMTP01 with cytotoxic, anti-inflammatory, and antimicrobial
activities. Nat. Prod. Res..

[78] Mohamed H., Ebrahim W., El-Neketi M., Awad M. F., Zhang H., Zhang Y., Song Y. (2022). In Vitro Phytobiological
Investigation
of Bioactive Secondary Metabolites from the *Malus domestica*-Derived Endophytic Fungus *Aspergillus tubingensis* Strain AN103. Molecules.

[79] Faisal S., Tariq M. H., Ullah R. (2023). Exploring the antibacterial,
antidiabetic, and anticancer potential of *Mentha arvensis* extract through in-silico and in-vitro analysis. BMC Complement Med. Ther..

[80] Castano-Duque L., Lebar M. D., Mack B. M., Lohmar J. M., Carter-Wientjes C. (2024). Investigating
the Impact of Flavonoids on *Aspergillus flavus*: Insights
into Cell Wall Damage and Biofilms. J. Fungi..

[81] Zhang J. D., Han L., Yan S. (2014). The non-metabolizable glucose analog D-glucal
inhibits aflatoxin biosynthesis and promotes kojic acid production
in *Aspergillus flavus*. BMC
Microbiol.

[82] Sabat J., Gupta N. (2009). Development of modified
medium for the enhancement in antifungal
activity of *P. steckii* (MF1 mangrove fungi) against *Verticillium wilt* pathogenic fungi of rose. Braz Arch. Biol. technol..

[83] Medina A., Schmidt-Heydt M., Rodríguez A. (2015). Impacts of environmental
stress on growth, secondary metabolite biosynthetic gene clusters
and metabolite production of xerotolerant/xerophilic fungi. Curr. Genet..

[84] Jia X., Song J., Wu Y., Feng S., Sun Z., Hu Y., Yu M., Han R., Zeng B. (2024). Strategies for the
Enhancement of Secondary Metabolite Production via Biosynthesis Gene
Cluster Regulation in *Aspergillus oryzae*. J. Fungi.

[85] Schlingmann G., Taniguchi T., He H., Bigelis R., Yang H. Y., Koehn F. E., Carter G. T., Berova N. (2007). Reassessing the structure
of pyranonigrin. J. Nat. Prod..

[86] Peng X., Wang Y., Zhu T., Zhu W. (2018). Pyrazinone derivatives
from the coral-derived *Aspergillus ochraceus* LCJ11–102
under high iodide salt. Arch Pharm. Res..

[87] Clifford D. R., Woodcock D. (1964). Metabolism of Phenoxyacetic
Acid by *Aspergillus
niger* van Tiegh. Nature.

[88] Padhi S., Masi M., Panda S. K., Luyten W., Cimmino A., Tayung K., Evidente A. (2020). Antimicrobial
secondary metabolites
of an endolichenic *Aspergillus niger* isolated from
lichen thallus of *Parmotrema ravum*. Nat. Prod. Res..

[89] Wang B., Li X., Yu D., Chen X., Tabudravu J., Deng H., Pan L. (2018). Deletion of
the epigenetic regulator
GcnE in *Aspergillus niger* FGSC A1279 activates the
production of multiple polyketide metabolites. Microbiol. Res..

[90] Barnes C. L., Steiner J. R., Torres E., Pacheco R., Marquez H. (1990). Structure
and absolute configuration of pyrophen, a novel pryrone derivative
of L-phenylalanine from *Aspergillus niger*. Int. J. Pept. Protein Res..

[91] Ma S., Ge L., Lu H., Yan J., Yang K. (2025). Two undescribed compounds
from *Aspergillus niger*, an endophytic fungus isolated
from *Camellia flavida*. Nat.
Prod. Res..

[92] Palys S., Pham T. T. M., Tsang A. (2020). Biosynthesis of Alkylcitric
Acids
in *Aspergillus niger* Involves Both Co-localized and
Unlinked Genes. Front. Microbiol..

[93] Sun Y., Liu W. C., Shi X., Zheng H. Z., Zheng Z. H., Lu X. H., Xing Y., Ji K., Liu M., Dong Y. S. (2021). Inducing secondary metabolite production
of *Aspergillus sydowii* through microbial co-culture
with *Bacillus subtilis*. Microb.
Cell Fact..

[94] Xiao Z., Lin S., Tan C., Lu Y., He L., Huang X., She Z. (2015). Asperlones A and B, dinaphthalenone derivatives from a mangrove endophytic
fungus *Aspergillus* sp. 16–5C. Mar. Drugs.

[95] Betancur L. A., Forero A. M., Vinchira-Villarraga D.
M., Cárdenas J. D., Romero-Otero A., Chagas F. O., Pupo M. T., Castellanos L., Ramos F. A. (2020). NMR-based metabolic profiling to follow the production
of anti-phytopathogenic compounds in the culture of the marine strain *Streptomyces* sp. PNM-9. Microbiol.
Res..

[96] Ovenden S. P., Sberna G., Tait R. M., Wildman H. G., Patel R., Li B., Steffy K., Nguyen N., Meurer-Grimes B. M. (2004). A diketopiperazine
dimer from a marine-derived isolate of *Aspergillus niger*. J. Nat. Prod..

[97] Yang S. W., Chan T. M., Terracciano J., Loebenberg D., Patel M., Chu M. (2005). Structure elucidation
of Sch 725674
from *Aspergillus* sp. J. Antibiot..

[98] Fraga M. E., Santana D. M., Gatti M. J., Direito G. M., Cavaglieri L. R., Rosa C. A. (2008). Characterization
of *Aspergillus* species
based on fatty acid profiles. Mem. Inst. Oswaldo
Cruz.

[99] Wu Y. R., Yin G. P., Gao H. L., Wang X. B., Yang M. H., Kong L. Y. (2019). Asperfuranones A-C,
3­(2H)-furanone derivatives from
the fungus *Aspergillus* sp. and the configuration
reassignment of their eighteen analogues. Fitoterapia.

[100] Nogueira J. M. F., Fernandes P. J. P., Nascimento A. M. D. (2003). Composition
of volatiles of banana cultivars from Madeira Island. Phytochem. Anal..

[101] Li H., Zhang R., Cao F., Wang J., Hu Z., Zhang Y. (2020). Proversilins A-E, Drimane-Type Sesquiterpenoids from the Endophytic *Aspergillus versicolor*. J. Nat. Prod..

[102] Nemec T., Jernejc K., Cimerman A. (1997). Sterols and fatty acids
of different Aspergillus species. FEMS Microbiol.
Lett..

[103] Ding L., Ren L., Li S., Song J., Han Z., He S., Xu S. (2019). Production of New Antibacterial 4-Hydroxy-*α*-Pyrones by a Marine Fungus *Aspergillus niger* Cultivated
in Solid Medium. Mar. drugs.

[104] Sumalatha M., Munikishore R., Rammohan A., Gunasekar D., Kumar K. A., Reddy K. K., Azad R., Reddanna P., Bodo B. (2015). Isoorientin, a Selective
Inhibitor of Cyclooxygenase-2 (COX-2) from
the Tubers of *Pueraria tuberosa*. Nat. Prod. Commun..

[105] Yuyama K., Nakamura Y., Tateyama R., Arakaki R., Tsutsui T., Ishimaru N. (2020). Study of the pharmacokinetics
of
eriodictyol-6-C-β-d-glucoside, a flavonoid of rooibos (Aspalathus
linearis) extract, after its oral administration in mice. J. Chromatogr. B.

[106] Clericuzio M., Mella M., Toma L., Finzi P. V., Vidari G. (2002). Atlanticones, New Protoilludane Sesquiterpenes from
the Mushroom *Lactarius atlanticus* (Basidiomycetes). Eur. J. Org. Chem..

[107] Fontaine T. (2017). Sphingolipids from the human fungal
pathogen *Aspergillus fumigatus*. Biochim..

[108] Zhao H., Xie Y., Li Z., Wei L., Ai R. (2025). Optimization of Fermentation
Conditions for Increasing Erucamide
Content in *Bacillus megaterium* Using Several Accelerants. Microorganisms.

[109] Kdimy A., Kim S. J., Ali Z., Khan M. I. H., Tripathi S. K., El Hajjaji S., Le H. V. (2023). Isolation of Two
Plasticizers, Bis­(2-ethylhexyl) Terephthalate and Bis­(2-ethylhexyl)
Phthalate, from *Capparis spinosa* L. Leaves. Chem. Biodivers..

[110] Amade P., Mallea M., Bouaïcha N. (1994). Isolation,
structural identification and biological activity of two metabolites
produced by *Penicillium olsonii* Bainier and Sartory. J. Antibiot..

[111] Lotfy W. A., Hassan S. W., Abd El-aal A. A., Ghanem K. M. (2019). Enhanced production of di-(2-ethylhexyl) phthalate
(DEHP) by *Bacillus subtilis* AD35 using response surface
methodology (RSM). Biotechnol. Biotechnol. Equip..

[112] Boros C., Smith C. J., Vasina Y., Che Y., Dix A. B., Darveaux B., Pearce C. (2006). Isolation and identification
of the icosalides--cyclic peptolides with selective antibiotic and
cytotoxic activities. J. Antibiot..

[113] Shang C., Gu Y., Koyama T. (2021). Major triterpenes,
cycloeucalenone and 31-norcyclolaudenone as inhibitors against both
α-glucosidase and α-amylase in banana peel. Int. J. Food Sci. Technol..

[114] Hasegawa Y., Fukuda T., Hagimori K., Tomoda H., O̅mura S. (2007). Tensyuic acids, new antibiotics produced
by *Aspergillus niger* FKI-2342. Chem.
Pharm. Bull..

[115] Gun Lee D., Yub Shin S., Maeng C.-Y., Zhu Jin Z., Lyong Kim K., Hahm K.-S. (1999). Isolation and characterization of
a novel antifungal peptide from *Aspergillus niger*. Biochem. Biophys. Res. Commun..

[116] Yao F. H., Liang X., Qi S. H. (2021). Eight new
cyclopentenone
and cyclohexenone derivatives from the marine-derived fungus *Aspergillus* sp. SCSIO 41501 by OSMAC strategy. Nat. Prod. Res..

[117] Lee I. K., Seok S. J., Kim W. K., Yun B. S. (2006). Hispidin
derivatives from the mushroom *Inonotus xeranticus* and their antioxidant activity. J. Nat. Prod..

[118] Liu S., Wang H., Su M., Hwang G. J., Hong J., Jung J. H. (2017). New metabolites
from the sponge-derived fungus *Aspergillus sydowii* J05B-7F-4. Nat.
Prod. Res..

[119] Kawahara T., Nagai A., Takagi M., Shin-Ya K. (2012). JBIR-137 and
JBIR-138, new secondary metabolites from *Aspergillus* sp. fA75. J. Antibiot..

[120] Qiao M. F., Ji N. Y., Miao F. P., Yin X. L. (2011). Steroids
and an oxylipin from an algicolous isolate of *Aspergillus
flavus*. Magn. Reson. Chem..

[121] Mutavski Z., Jerković I., Nikolić N. Ć., Radman S., Flanjak I., Aladić K., Šubarić D., Vulić J., Jokić S. (2024). Comprehensive Phytochemical Profiling of *Ulva
Lactuca* from the Adriatic Sea. Int.
J. Mol. Sci..

[122] Wen H., Chen C., Sun W., Zang Y., Li Q., Wang W., Zeng F., Liu J., Zhou Y., Zhou Q., Wang J., Luo Z., Zhu H., Zhang Y. (2019). Phenolic C-Glycosides and Aglycones from Marine-Derived Aspergillus
sp. and Their Anti-Inflammatory Activities. J. Nat. Prod..

[123] Pérez-Castorena A. L., Arciniegas A., Guzman S. L., Villaseñor J.
L., Romo de Vivar A. (2006). Eremophilanes
from Senecio mairetianus and some reaction products. J. Nat. Prod..

[124] El-Gazzar N., Said L., Al-Otibi F. O., AbdelGawwad M. R., Rabie G. (2025). Antimicrobial and cytotoxic activities
of natural (Z)-13-docosenamide
derived from *Penicillium chrysogenum*. Front. Cell. Infect. Microbiol..

[125] Esua M. F., Rauwald J. W. (2006). Novel bioactive
maloyl glucans from
aloe vera gel: isolation, structure elucidation and in vitro bioassays. Carbohydr. Res..

[126] Liu Y., Chen S., Liu Z., Lu Y., Xia G., Liu H., He L., She Z. (2015). Bioactive Metabolites
from Mangrove
Endophytic Fungus *Aspergillus* sp. 16–5B. Mar. Drugs.

[127] Fuchser J., Grabley S., Noltemeyer M., Philipps S., Thiericke R., Zeeck A. (1994). Secondary Metabolites
by Chemical Screening, 28. Aspinonene, a New Multifunctional Fungal
Metabolite. Liebigs Ann. Chem..

[128] Ibrahim S. R. M., Mohamed S. G. A., Alsaadi B. H., Althubyani M. M., Awari Z. I., Hussein H. G. A., Aljohani A. A., Albasri J. F., Faraj S. A., Mohamed G. A. (2023). Secondary Metabolites,
Biological
Activities, and Industrial and Biotechnological Importance of *Aspergillus sydowii*. Mar. Drugs.

[129] Kim Y. P., Tomoda H., Iizima K., Fukuda T., Matsumoto A., Takahashi Y., Omura S. (2003). Takanawaenes, novel
antifungal antibiotics produced by *Streptomyces* sp.
K99–5278. I. Taxonomy, fermentation, isolation and biological
properties. J. Antibiot..

[130] He J., Wijeratne E. M. K., Bashyal B. P., Zhan J., Seliga C. J., Liu M. X., Pierson E. E., Pierson L. S., VanEtten H. D., Gunatilaka A. A. L. (2004). Cytotoxic
and Other Metabolites of
Aspergillus Inhabiting the Rhizosphere of Sonoran Desert Plants1. J. Nat. Prod..

[131] Qi C., Liu M., Zhou Q., Gao W., Chen C., Lai Y., Hu Z., Xue Y., Zhang J., Li D., Li X. N., Zhang Q., Wang J., Zhu H., Zhang Y. (2018). BACE1 Inhibitory Meroterpenoids
from *Aspergillus terreus*. J.
Nat. Prod..

[132] Rousta N., Ferreira J. A., Taherzadeh M. J. (2021). Production
of L-carnitine-enriched edible filamentous fungal biomass through
submerged cultivation. Bioengineered.

