# Supplementary material for: Unveiling Valuable Secondary Metabolites from the Bioconversion of Banana (Musa balbisiana) Peel-Derived Biomass with Aspergillus niger. Metabolomic Insights into the Chemical Profiles
Source: ACS Omega. 2026 Jan 21;11(4):6084–109. doi: 10.1021/acsomega.5c10614 (PMC12878763; doi:10.1021/acsomega.5c10614)
Supplement: Supplementary file 1 [file ao5c10614_si_001.pdf]

Supplementary material

Unveiling valuable secondary metabolites from the bioconversion of banana (*Musa balbisiana*) peels-derived biomass with *Aspergillus niger*. Metabolomic insights into the chemical profiles.

Jhuly Wellen Ferreira Lacerda[1], Giovanna Amaral Filipe[2], Lucas Pradi[1], Tatiane de Andrade Maranhão[1], Diogo Robl[2], Louis Pergaud Sandjo[1, *]

[1]Department of Chemistry, Federal University of Santa Catarina, Campus Universitário-Trindade, 88040-900 Florianópolis, SC, Brazil;

[2]Laboratory of Microorganisms and Biotechnological Processes, Department of Microbiology, Parasitology and Immunology, Federal University of Santa Catarina, Florianópolis 88040-900, Brazil

*Correspondence: LPS: Phone: +5548 37213624, E-mail: p.l.sandjo@ufsc.br

1. Characterization and identification of the fungus *Aspergillus niger*

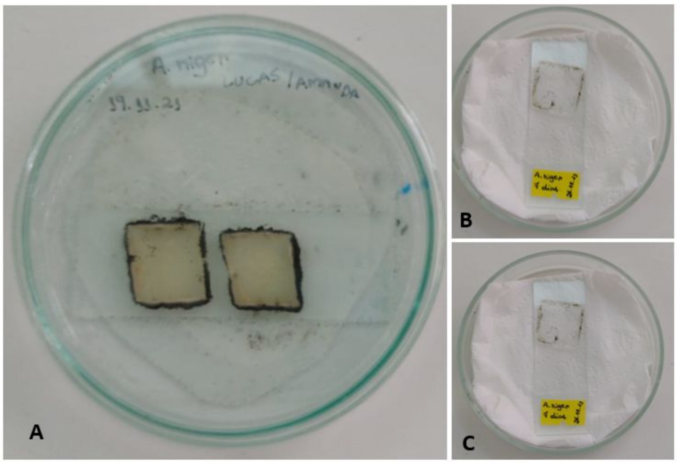

Figure S1. Microculture and slides for observation of fungal material under a microscope. **A:** microculture, **B:** slide at 7 days, **C:** slide at 14 days.

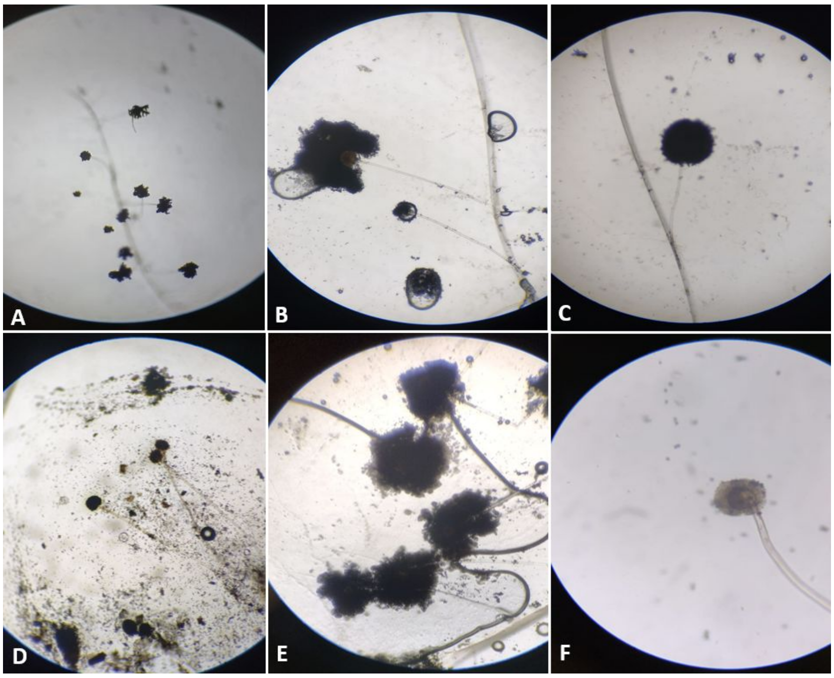

Figure S2. Micromorphological aspect of the fungus evaluated after 7 and 14 days of cultivation (microculture). **A-C:** 7 days of microculture, **D-F:** 14 days of microculture. In A and D: 20x magnificated; B, C, E and F: 40x magnificated.

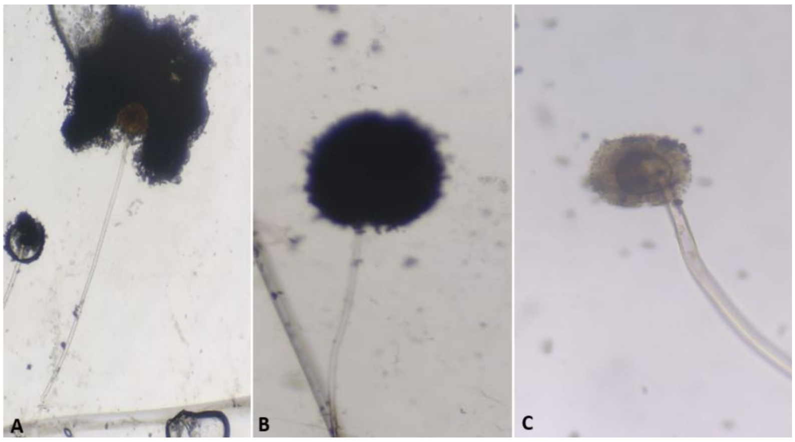

Figure S3. Details of the microscopic structures (conidiophore) of the fungal isolate evaluated. **A** and **B**: 7 days of microculture, **C:** 14 days of microculture

2. Chromatograms obtained from MS^E UPLC-ESI-QTOF-MS analysis

Banana peel (+)

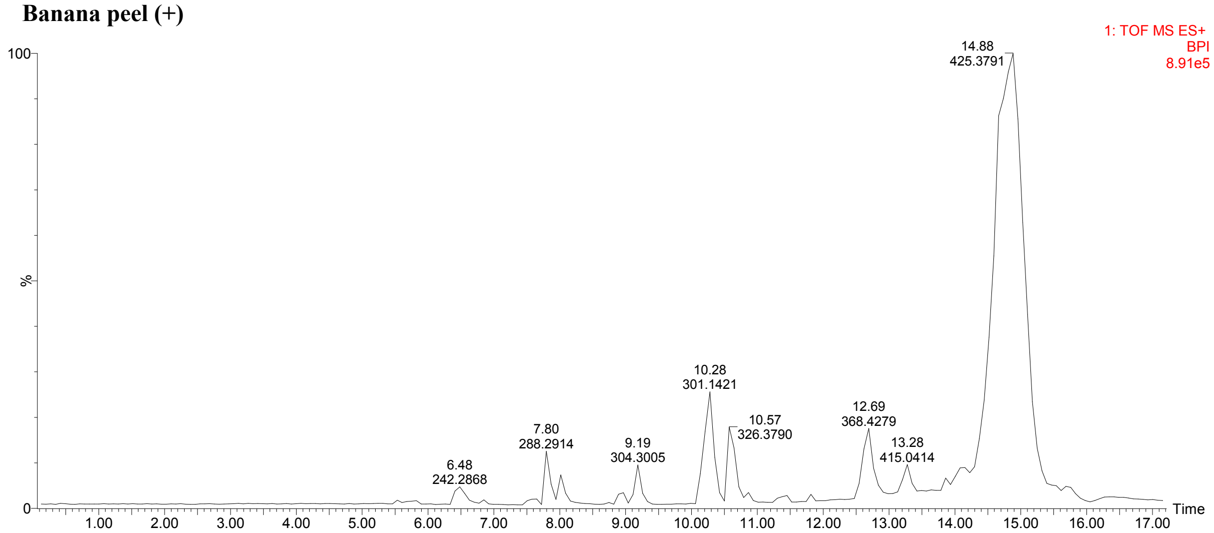

Banana peel (-)

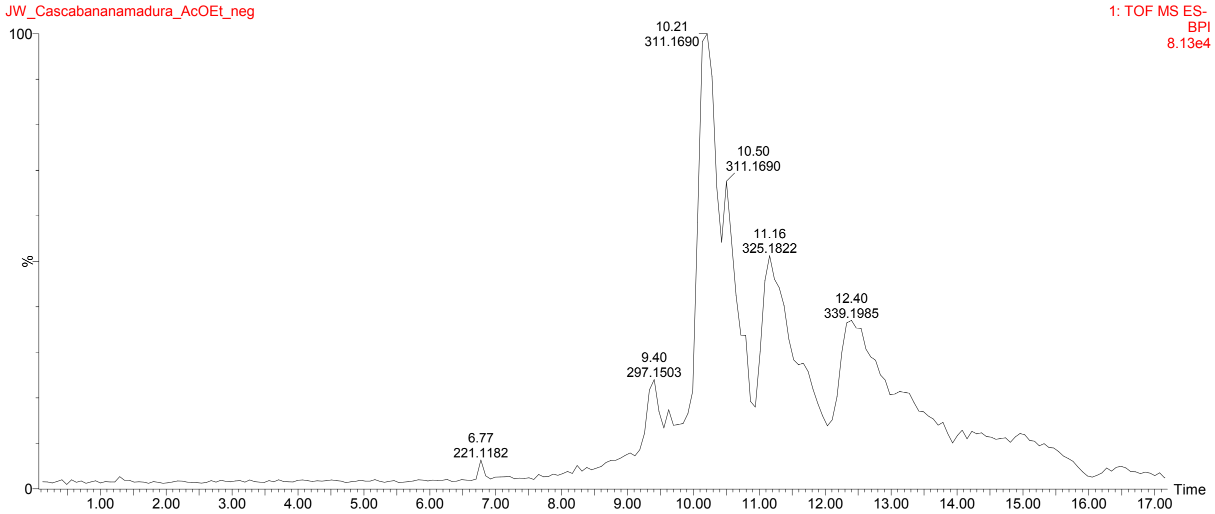

Figure S4: Chromatograms obtained in positive and negative mode by UPLC-ESI-MS/MS for non-inoculated banana peel (**BP**).

BPS_An_14 days (+)

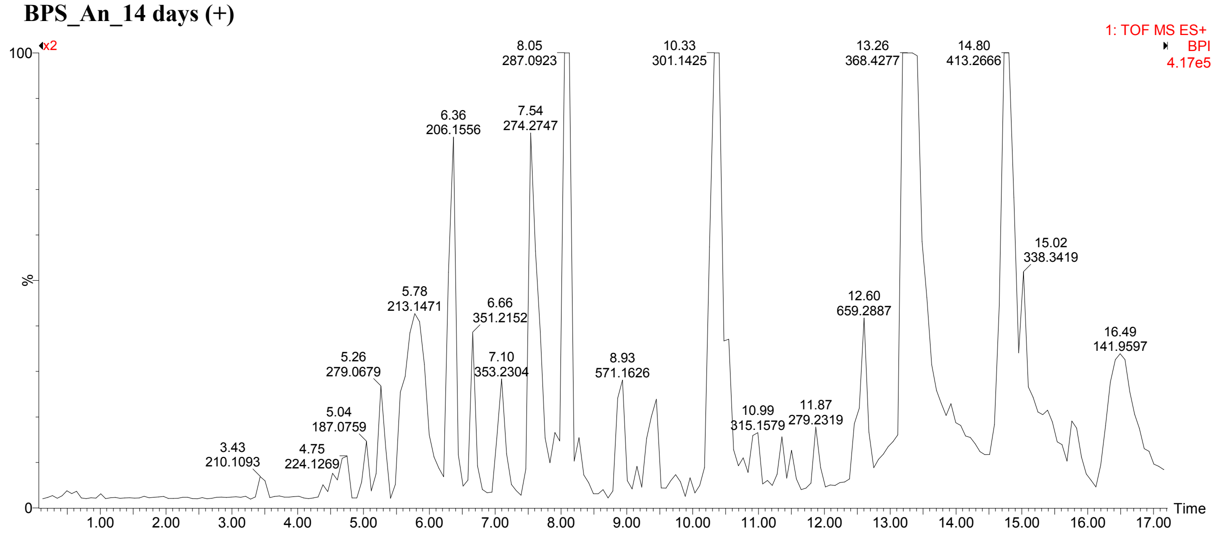

BPS_An_21 days (+)

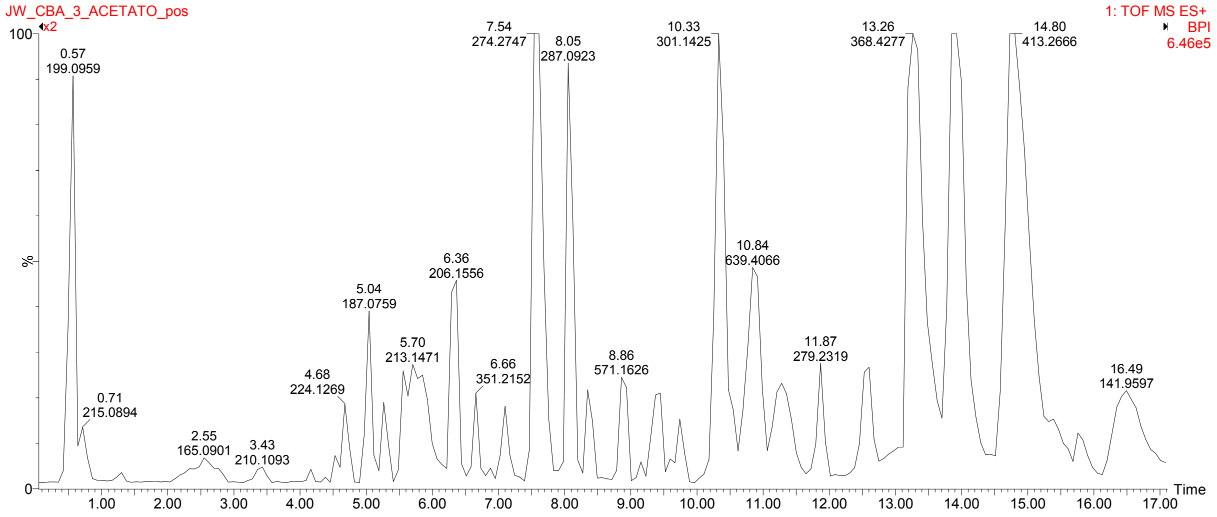

BPS_An_28 days (+)

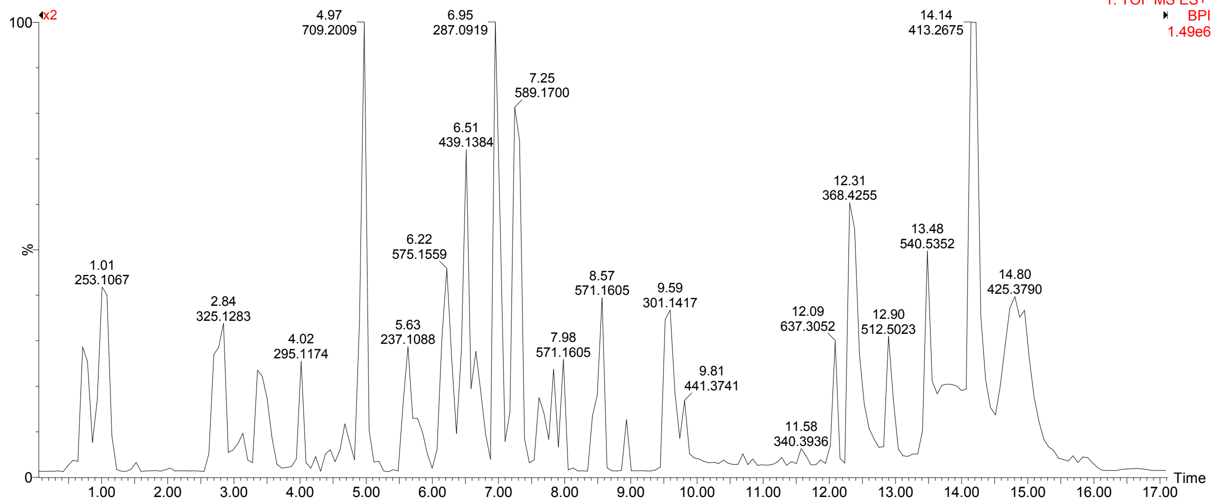

Figure S5: Chromatograms obtained in positive mode by UPLC-ESI-MS/MS for the extract of the fungus *Aspergillus niger* grown on banana peel with starch (**BPS_An**) in 14, 21 and 28 days.

BPS_An_14 days (-)

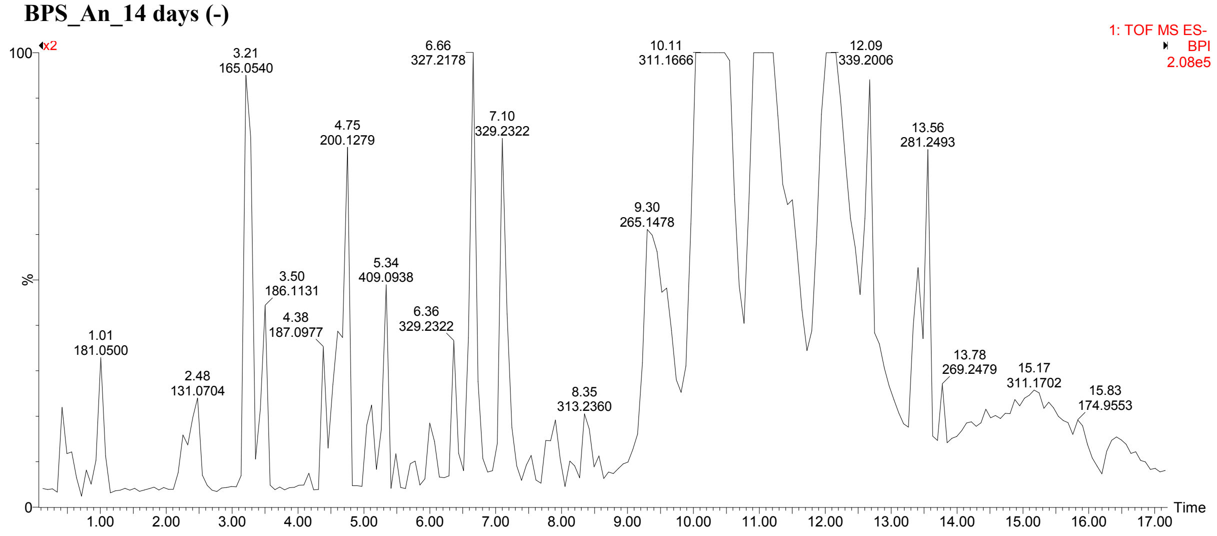

BPS_An_21 days (-)

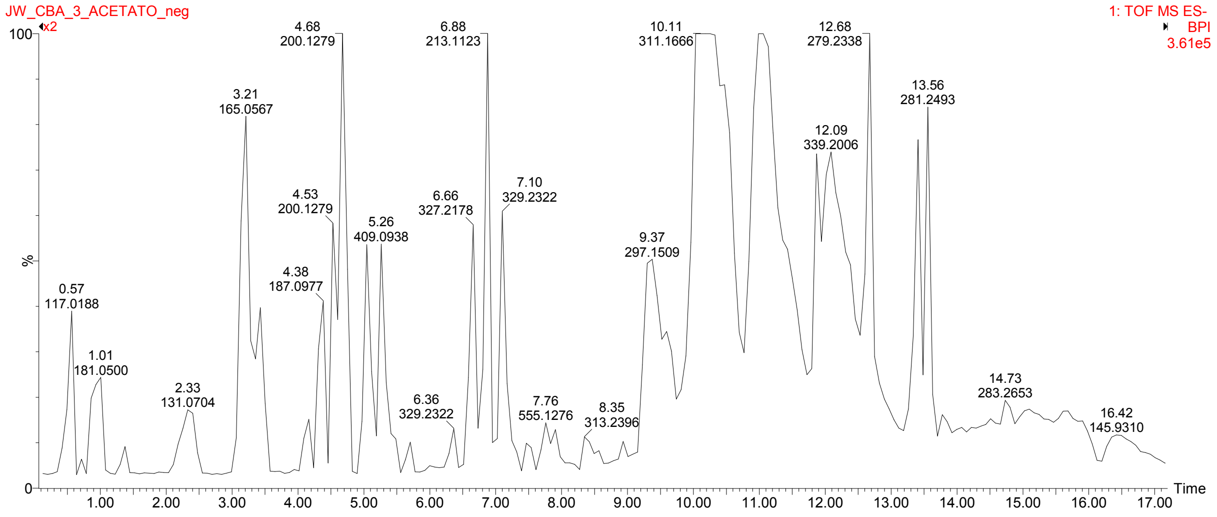

BPS_An_28 days (-)

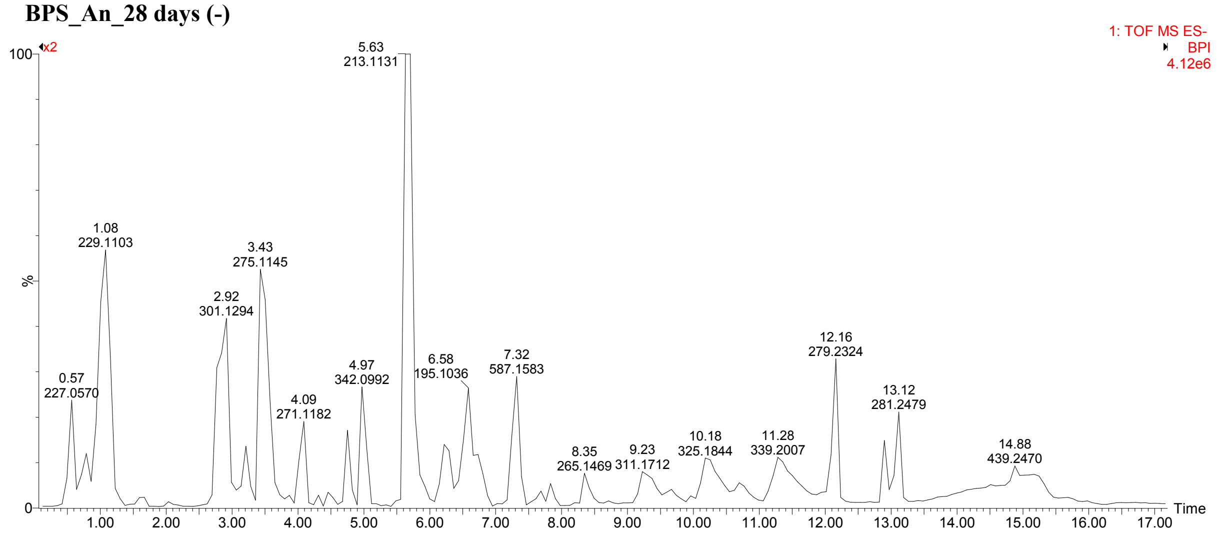

Figure S6: Chromatograms obtained in negative mode by UPLC-ESI-MS/MS for the extract of the fungus *Aspergillus niger* grown on banana peel with starch (**BPS_An**) in 14, 21 and 28 days.

BP_An_14 days (+)

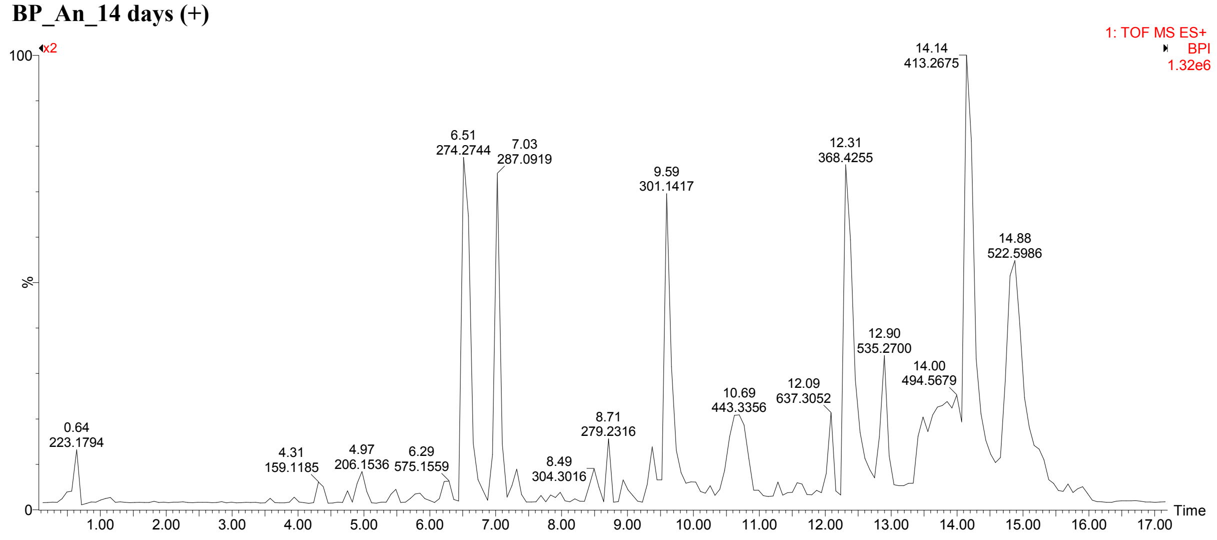

BP_An_21 days (+)

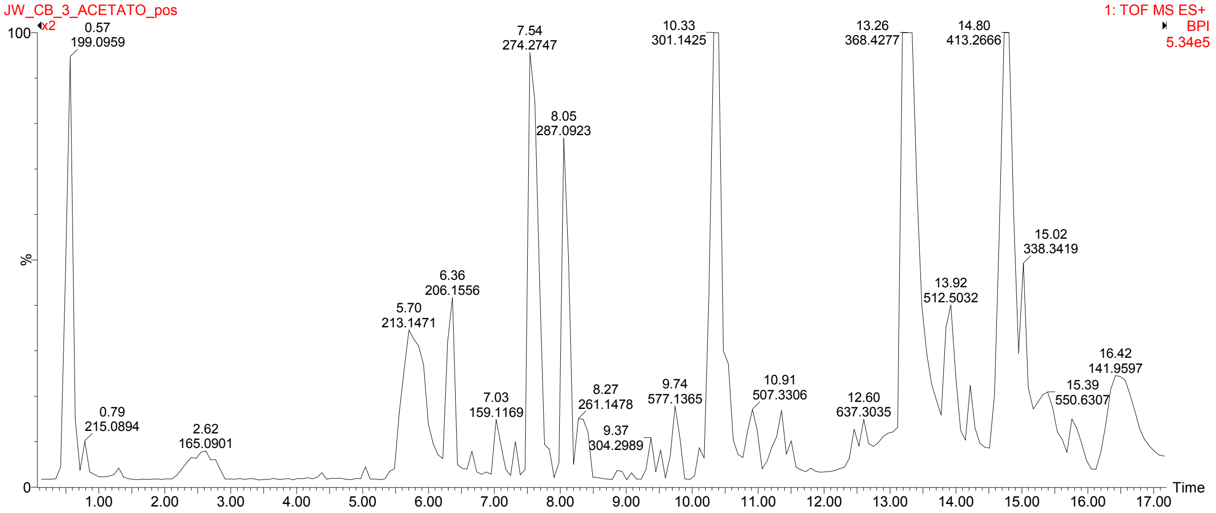

BP_An_28 days (+)

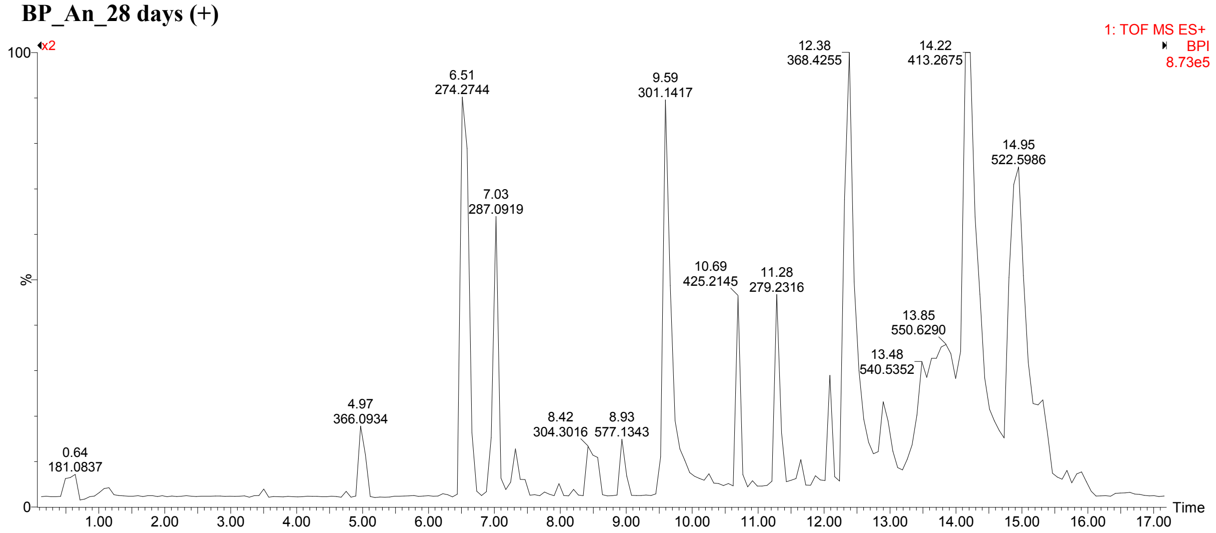

Figure S7: Chromatograms obtained in positive mode by UPLC-ESI-MS/MS for the extract of the fungus *Aspergillus niger* grown on banana peel (**BP_An**) in 14, 21 and 28 days.

BP_An_14 days (-)

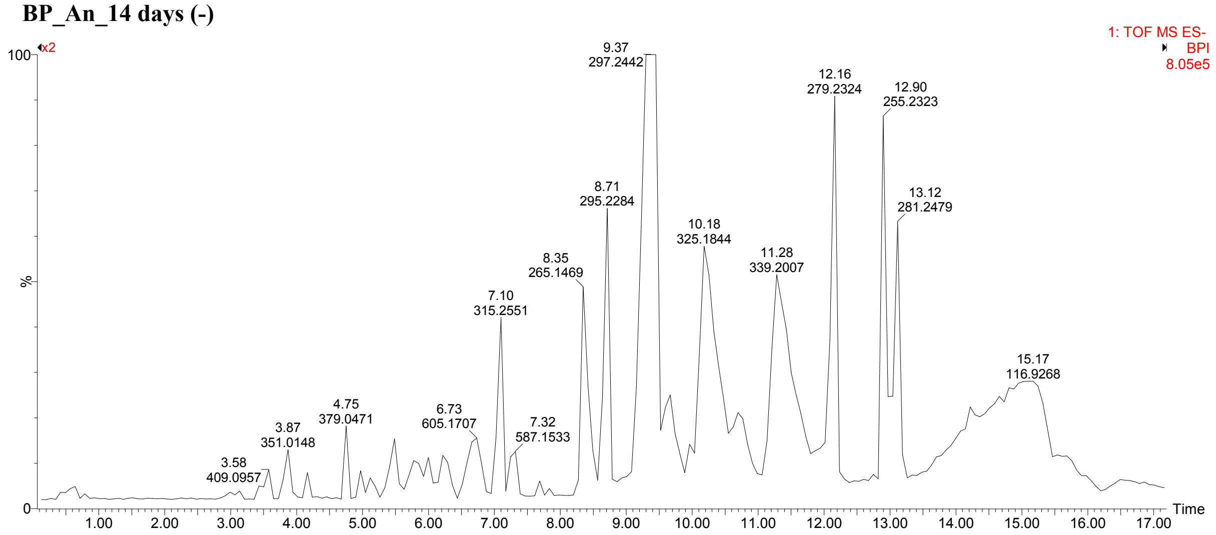

BP_An_21 days (-)

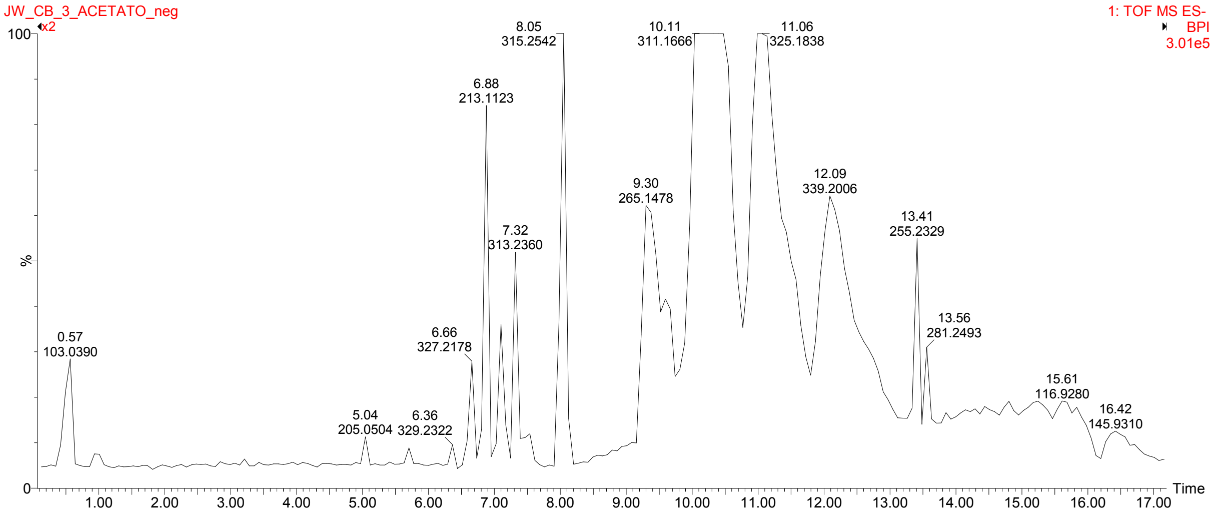

BP_An_28 days (-)

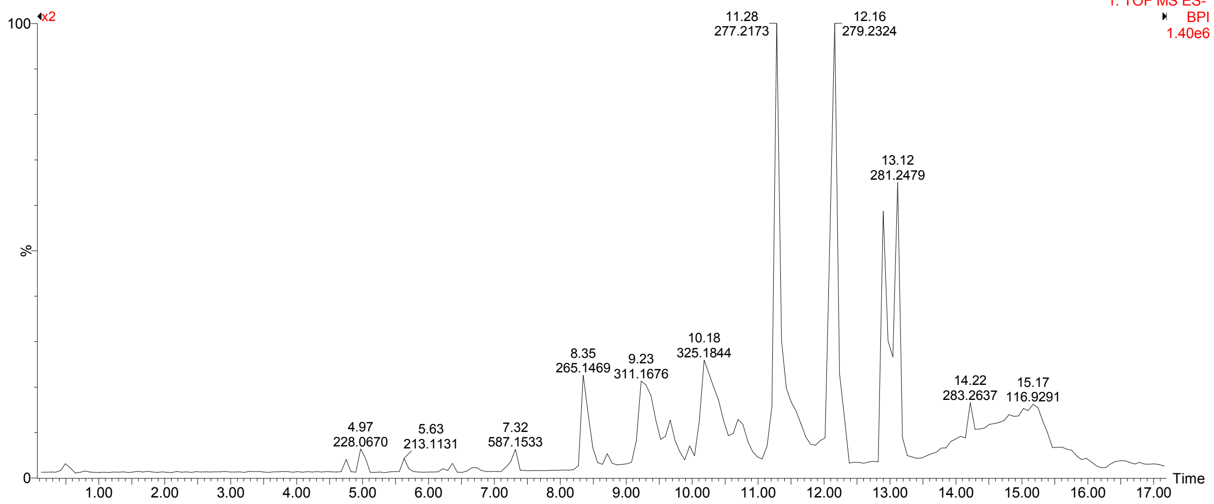

Figure S8: Chromatograms obtained in negative mode by UPLC-ESI-MS/MS for the extract of the fungus *Aspergillus niger* grown on banana peel (**BP_An**) in 14, 21 and 28 days.

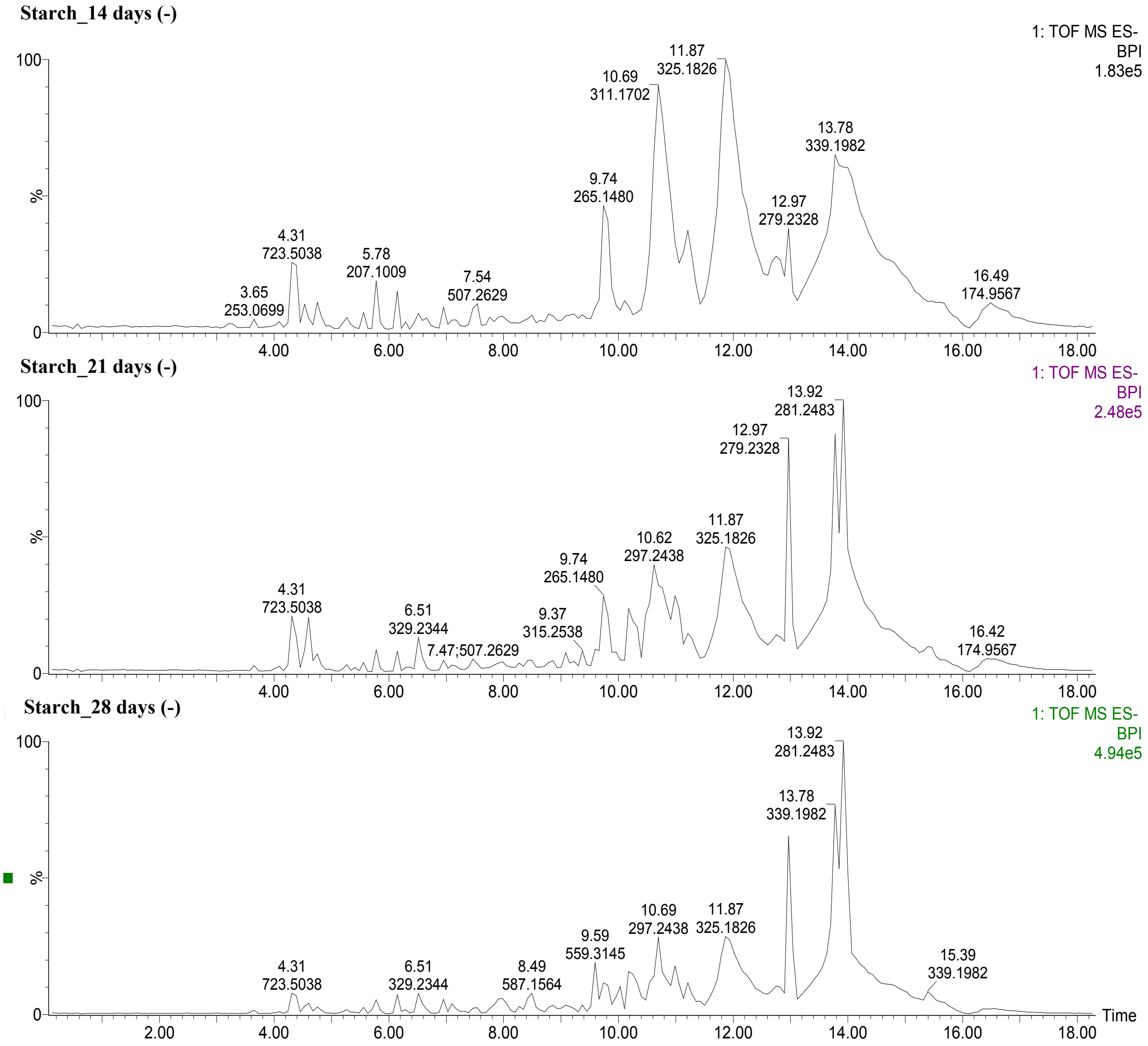

Figure S9: Chromatograms obtained in negative mode by UPLC-ESI-MS/MS for the extract of the fungus *Aspergillus niger* grown on starch in 14, 21 and 28 days

Table S1**:** Identification of metabolites using UPLC-ESI-HRMS analysis in positive and negative modes for extracts obtained from the fungus *A. niger* grown on banana peel (BP), at 14, 21 and 28 days.

nº	t_R (min)	Molecular formula	Adduct type	Precursor *m/z*	Error (ppm)	MS/MS fragmentationions ESI+	MS/MS fragmentationions ESI-	Proposed identification	Time (day)
3	1.63	$C_{13}H_{22}N_2O$	$[M+H]^+$	223.1811	-0.95	151.0390; 129.1396; 95.0494		-	14, **21**
5	2.67	$C_{13}H_{22}N_2O_2$	$[M+H]^+$	239.1758	-1.66	192.1411; 136.0764; 127.1219; 103.0764		Ochramide B	14, 21
8	5.04	$C_{18}H_{12}O_6$	$[M-H]^-$	323.0550	3.43		295.0615; 283.0654; 271.0587; 256.0394; 183.0181; 133.0284; 123.0448	Atromentin or derivatives	28
14	5.30	$C_{22}H_{18}O_8$	$[M+H]^+$ $[M-H]^-$	411.1079 409.0920	-1.11 2.17	379.0824; 369.0992; 337.0717; 278.0613; 266.0918; 235.1395; 193.0858; 181.0838;	362.0715; 345.0407; 333.0774; 318.0530; 303.0332; 289.0515; 274.0631	Orlandin or derivatives	14, 21, 28
15	5.47	$C_{18}H_8O_8$	$[M-H]^-$	351.0136	2.96		323.0521; 295.0580; 266.0203; 249.0164;	Thelephoric acid	14, 21, **28**

							223.0400;<br>211.0415		
20	6.15	$C_{20}H_{12}O_8$	[M+H] +<br>[M-H] -	381.0604<br>379.0450	0.25<br>2.48	367.0833;<br>353.1073;<br>329.0656;<br>311.0558;<br>299.0916;<br>285.0794;<br>258.0537; 242.0577	361.0362;<br>349.0333;<br>333.0400;<br>317.0462;<br>291.0316;<br>281.0439;<br>245.0463;<br>230.0224	Asperlone B	14, 21,<br>28
21	6.31<br>6.33	$C_{18}H_{17}NO_6$	[M+H] +<br>[M-H] -	344.1132<br>342.0970	-0.98<br>3.82	324.1006;<br>283.0837;<br>274.0501;<br>268.0294;<br>252.0630;<br>230.0826;<br>213.0548;<br>185.0512; 157.0647	311.1690;<br>288.0778;<br>269.0169;<br>250.0480;<br>228.0634;<br>210.0554;<br>195.0620;<br>159.0435;<br>141.0699;<br>130.0514;<br>113.0236	Pestalamide A	28
22	6.31	$C_{13}H_{19}NO$	[M+H] +	206.1546	-3.21	163.0776;133.0606;<br>105.0701		2-Methyl-N-(2-<br>phenylethyl)butanamide	14, 21
23	6.33	$C_{23}H_{20}O_8$	[M+H] +<br>[M-H] -	425.1232<br>423.1071	-0.25<br>-2.11	366.0970;<br>351.0897;<br>283.0837;<br>274.0501;<br>268.0294;<br>252.0630;<br>230.0826;	396.0144;<br>364.0803;<br>347.0933;<br>332.0695;<br>311.1690;<br>288.0778;<br>229.0506;	Demethylkotanin or<br>Isokotanin B	14, 28

						213.0548; 185.0512; 157.0647	250.0480; 228.0634; 185.0620; 141.0699		
24	6.33	$C_{13}H_{11}NO_3$	[M-H] -	228.0656	4.44		210.0643; 185.0620; 183.0126; 159.0435; 153.0550; 141.0699; 130.0514; 113.0236	Carbonarone A or B	28
29	6.83	$C_{11}H_{18}O_4$	[M-H] -	213.1126	2.95		193.0864; 183.0154; 171.1056; 169.1219; 151.0407; 125.0969	hexylitaconic acid	21, 28
33	7.29	$C_{18}H_{34}O_4$	[M+Na] + [M-H] -	337.2350 313.2372	-1.42 3.92	297.2419; 295.1487; 283.1498; 279.2318; 241.1443	297.1080; 285.2082; 262.1516; 245.1296; 227.0883; 214.1546; 172.1085	Related to 12,13-dihydroxyoctadec-9-enoic acid	21
37	7.57	$C_{24}H_{22}O_8$	[M+H] +	439.1388	-0.13	397.1312; 365.1042; 228.1158; 307.0990; 278.0920		Kotanin	14, 21, 28
40	7.71	$C_{31}H_{24}O_{10}$	[M-H] -	555.1280	3.0		456.0857; 442.0659;	Aurasperone D or derivative	14, 21, 28

							385.0719; 339.1985; 311.1654;		
43	7.98	$C_{18}H_{36}O_4$	$[M+Na]^+$<br>$[M-H]^-$	339.2511<br>315.2531	1.64<br>3.11		297.2421; 219.1349; 197.1553; 141.1307; 127.1128	Related to 9,10-dihydroxystearic acid	14, 21
47	8.13	$C_{16}H_{14}O_5$	$[M+H]^+$	287.0922	-2.80	272.0673; 243.0670; 229.0512; 214.0645; 201.0546; 168.0594; 141.0695; 123.0410		Flavasperone or isomer	14, 21, 28
51	8.27	$C_{32}H_{28}O_{11}$	$[M+H]^+$<br>$[M-H]^-$	589.1703<br>587.1542	0.24<br>2.87	557.1477; 531.1325; 516.1033; 505.1418; 484.0798; 432.0794; 365.1432; 351.1318	555.1265; 503.1340; 488.1139; 473.0853; 456.0813; 404.0924; 389.0670; 311.1654; 183.0126	Fonsecinone B or derivatives	14, 21, 28
54	9.34	$C_{32}H_{26}O_{10}$	$[M+H]^+$	571.1608	-1.98	556.1397; 514.1268; 524.1033; 498.0956; 472.1189; 442.1100; 337.1167; 285.0760; 272.0706		Aurasperone A or derivatives	21, 28

62	11.29	$C_{20}H_{34}O_8$	$[M+H]^+$ $[M+Na]^+$	403.2326 425.2150	0.11	385.2927; 365.1510; 341.2694; 311.2939; 297.2384	Related to Botcinic acid	BP, 28
63	11.87	$C_{18}H_{30}O_2$	$[M+H]^+$	279.2327	-1.23	261.224; 251.1259; 242.2868	Related to Linolenic acid	BP, 21, **28**

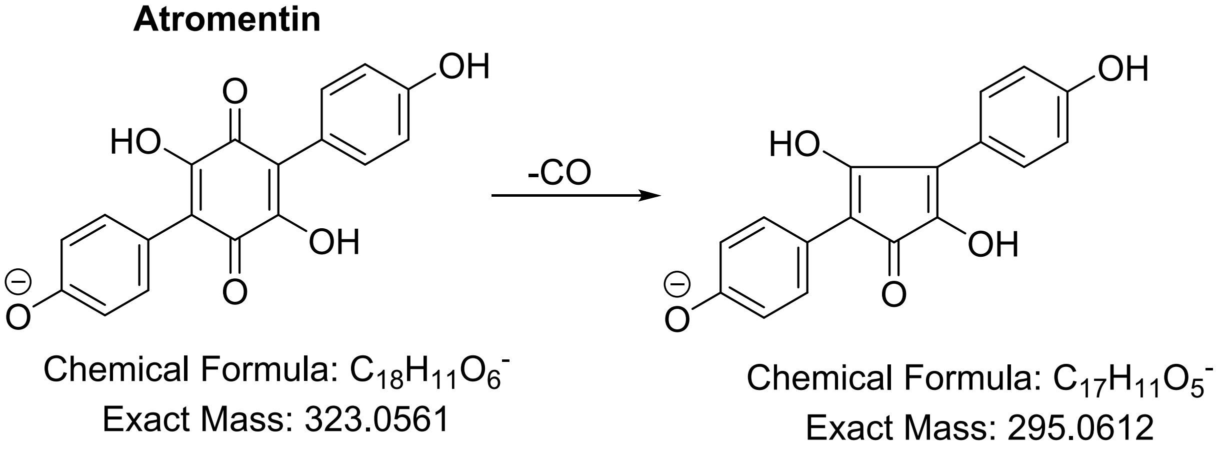

Scheme S1: Fragmentation pattern of atromentin

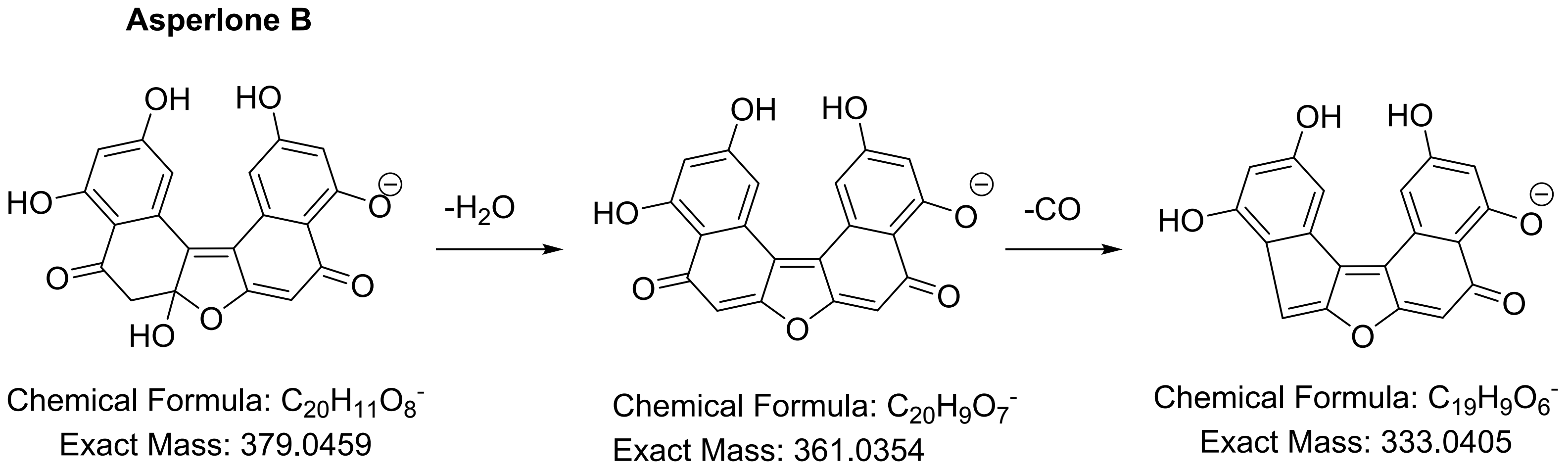

Scheme S2: Fragmentation pattern of Asperlone B

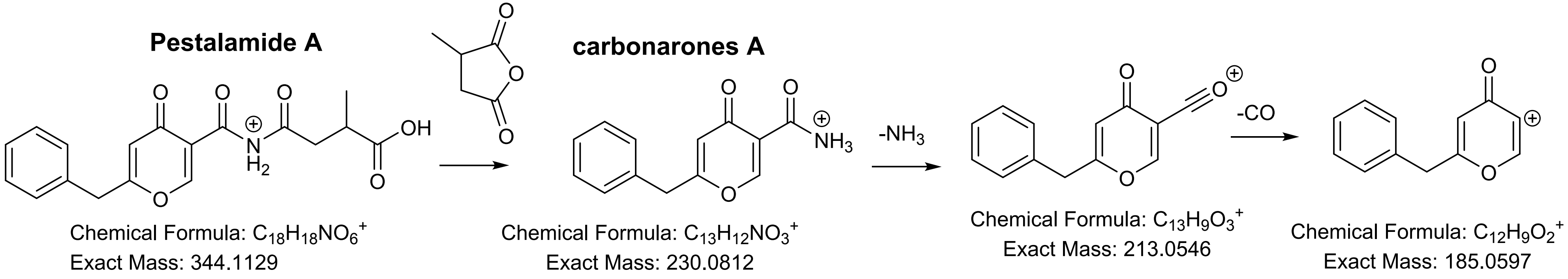

Scheme S3: Fragmentation pattern of pestalamide A

Carbonarone A

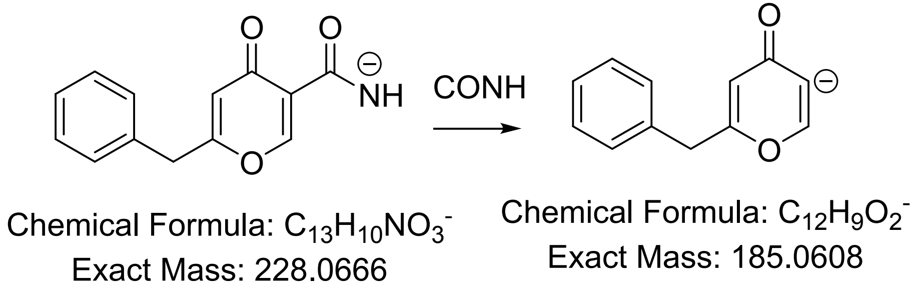

Scheme S4: Fragmentation pattern of Carbonarone A

Hexylitaconic acid

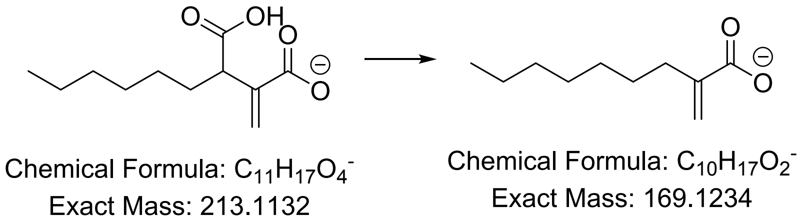

Scheme S5: Fragmentation pattern of Hexylitaconic acid

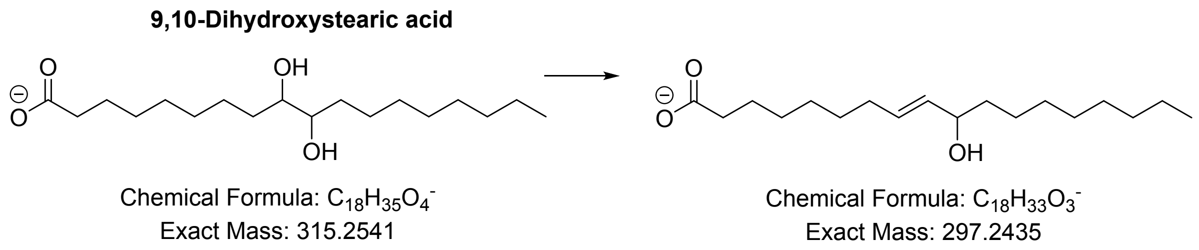

Scheme S6: Fragmentation pattern of 9,10-dihydroxystearic acid

Table S2 : Identification of metabolites using UPLC-ESI-HRMS analysis in positive and negative modes for extracts obtained from the fungus *A. niger* grown on banana peel with starch (BPS), at 14, 21 and 28 days

**nº**	**t_R (min)**	**Molecular formula**	**Adduct type**	**Precursor *m/z***	**Error (ppm)**	**MS/MS fragmentationions**		**Proposed identification**	**Score**
						**ESI+**	**ESI-**		
9	5.11	$C_{13}H_{11}NO_3$	$[M+H]^+$	230.0826	-3.62	213.0548; 212.0713; 207.0671; 187.0772;		**Carbonarone A or B**	28
12	5.06	$C_{12}H_{10}O_2$	$[M+H]^+$	187.0762	-4.54	161.0680; 157.0672; 128.0640; 115.0550; 105.0344; 91.0545		Hexylitaconic acid	21, 28
13	5.21	$C_{12}H_{20}O_7$	$[M-H]^-$	275.1113	2.99		251.0914; 240.0443; 195.1027; 185.1177; 171.1029;	Hexylcitric acid	28

							149.0248; 141.1282; 123.0448		
23	6.33	$C_{23}H_{20}O_8$	$[M+H]^+$ $[M-H]^-$	425.1219 423.1071	-0.25 -2.11	383.1090; 366.0970; 351.0897; 344.1113; 324.1006; 252.0630; 230.0826; 213.0548; 230.1533; 185.0612; 161.0608; 157.0647		Demethylkotanin or Isokotanin B	**21, 28**
29	6.83	$C_{11}H_{18}O_4$	$[M+Na]^+$ $[M-H]^-$	237.1102 213.1133	-0.33 2.49	197.1175; 177.0926; 169.1238; 151.1119; 149.0957; 123.1181; 118.0653	195.1027; 185.1177; 169.1245; 151.1137; 141.1282	Tensyuic acid A or F	28
38	7.63	$C_{24}H_{22}O_8$	$[M+H]^+$	439.1406	-0.81	397.1271; 365.1042; 309.1336; 288.2914; 191.1449		Kotanin	28
47	8.13	$C_{16}H_{14}O_5$	$[M+H]^+$	287.0918	-2.80	272.0673; 243.0670; 229.0512; 214.0645;		Flavasperone or isomer	21, 28

						201.0575; 196.0513			
49	8.13	$C_{31}H_{24}O_{10}$	[M+H]$^+$	557.1477	-1.58	542.1234; 510.1021; 502.2206; 484.0843 343.0087; 325.0434; 287.0918 272.0673; 243.0670; 229.0512; 214.0645; 201.0575; 196.0513		Aurasperone D or derivative	21, **28**
52	8.87	$C_{32}H_{26}O_{10}$	[M+H]$^+$	571.1602	-0.046	556.1397; 514.1268; 498.0956; 472.1144; 457.0939; 318.2666; 243.0670; 195.1022; 170.1915		Aurasperone A or derivatives	**21,** 28
79	3.68	$C_{11}H_{16}O_6$	[M-H]$^-$	243.0859	2.92		229.1063; 223.0645; 205.0831; 185.1177; 161.0947; 139.1122; 125.0969	(-)-9-hydroxyhexylitaconic acid	28

80	3.76	$C_{11}H_{18}O_5$	[M+Na]+<br>[M-H]-	253.1052<br>229.1063	-2.41<br>2.30	213.1145;<br>207.0701;<br>195.1022;<br>177.0926	223.0645;<br>205.0831;<br>185.1177;<br>161.0947;<br>139.1122;	Aspergilactone A	28
82	4.43	$C_{16}H_{25}NO_4$	$[M-H]^-$	294.1691	4.34		237.1133;<br>213.1133;<br>211.0980;<br>203.0916;<br>169.1219	NI	28
83	4.56	$C_{16}H_{23}NO_4$	$[M+H]^+$	294.1708	-2.44	237.1154;<br>151.1119;<br>133.1025;<br>123.1159;<br>105.0701			28
86	4.86<br>4.95	$C_{14}H_{22}O_7$	$[M+Na]^+$<br>[M-H]-	325.1264<br>301.1298	-2.07<br>3.89		283.0275;<br>267.0563;<br>257.1379;<br>185.1177;<br>167.1074;<br>133.0289	Roridinic acid	28
94	5.54	$C_{16}H_{24}O_8$	$[M+Na]^+$<br>$[M-H]^-$	367.1366<br>343.1384	-0.76<br>3.32	323.0563;<br>273.0797;<br>251.0579;<br>214.0884;<br>195.1050;<br>177.0926;<br>149.0957;<br>131.0867;<br>131.0867	323.0557;<br>295.0580;<br>283.1171;<br>267.0563;<br>251.0914;<br>229.1063;<br>207.1020;<br>205.0831;<br>195.1027;<br>185.1177;	Macrosphelide I	28

							161.0947; 139.1122		
109	6.95	$C_{17}H_{24}O_7$	$[M+H]^+$	341.1616	-3.25	281.0760; 269.0478; 253.0766; 235.0643; 224.0482; 209.0870; 191.0743; 165.0230; 123.1181		Aspergilloid E	28
117	8.48	$C_{22}H_{34}O_8$	$[M+H]^+$<br>$[M-H]^-$	427.2324<br>425.2161	0.57<br>3.50	409.2231; 391.2138; 360.2379; 287.0952; 269.0712; 251.0611; 213.1115; 195.1022; 177.0926; 170.1915; 149.057; 131.0867; 121.1009	403.2082; 393.3466; 311.1690; 267.0563; 251.0914; 229.1063; 205.0831; 195.1027; 185.1177; 183.0126; 169.1219; 151.1137; 123.1174	NI	28
